# Supplementary material for: Real-world effects of medications for chronic obstructive pulmonary disease: protocol for a UK population-based non-interventional cohort study with validation against randomised trial results
Source: BMJ Open. 2018 Mar 25;8(3):e019475. doi: 10.1136/bmjopen-2017-019475 (PMC5875594; doi:10.1136/bmjopen-2017-019475)
Supplement: Supplementary file 1 [file bmjopen-2017-019475supp001.pdf]

Overview of algorithms to be used for detecting COPD, COPD exacerbations and pneumonia

Condition	Paper (author, year)	Algorithm description[1]	Validity[2]	Other notes
COPD	Quint et al, 2014[10]	- CPRD[3] diagnostic (Read) code for COPD	PPV[4]: 87% (78 – 92)	1. Comparison with gold standard of respiratory physician review of information obtained by questionnaire from GPs<br>2. 8 algorithms presented in total, PPVs ranging from 12 to 89
COPD exacerbation	Rothnie et al, 2016[12]	- CPRD diagnostic (Read) code for LRTI or Acute Exacerbation COPD (AECOPD) **OR**<br>- A prescription of a COPD-specific antibiotic combined with OCS for 5-14 days **OR**<br>- A record (Read code) of two or more respiratory symptoms of AECOPD with a prescription of COPD-specific antibiotics and/or OCS on the same day	PPV: 86% (83 – 88)<br>Sensitivity: 63% (55 – 70)	1. Comparison with gold standard of respiratory physician review of information obtained by questionnaire from GPs<br>2. 15 algorithms presented in total, PPVs ranging from 61% – 100%, sensitivities ranging from 1.6% – 63%
Pneumonia	Millet et al, 2013[11]	- CPRD diagnostic (Read) codes and HES[5] diagnostic (ICD-10) codes for pneumonia (identified as a subset of an initial search for LRTI codes)<br>- Records in both database within the 28 days considered the same illness-episode	No validation performed	

Note 1: Main algorithm presented in article and to be applied initially in COPD medications real-world effects study (details on other algorithms presented in paper provided in the "Other notes" column where appropriate). **Note 2**: Validity=measure of validity presented in article:result obtained (95% CI). **Note 3**: CPRD=UK Clinical Practice Research Datalink **Note 4**: PPV=positive predictive value **Note 5:** HES=Hospital Episode Statistics

LAMA codes

Product code	BNF header	Drug substance	Drug product
61176	compound bronchodilator preparations	vilanterol trifenatate/umeclidinium bromide	anoro ellipta 55micrograms/dose / 22micrograms/dose dry powder inhaler (glaxosmithkline uk ltd)
61490	compound bronchodilator preparations		umeclidinium bromide 65micrograms/dose / vilanterol 22micrograms/dose dry powder inhaler
35014	antimuscarinic bronchodilators	tiotropium bromide monohydrate	tiotropium bromide 18microgram inhalation powder capsules with device
6474	antimuscarinics	glycopyrronium bromide	robinul 1mg tablet (idis world medicines)
50577	antimuscarinic bronchodilators	tiotropium bromide	spiriva 18microgram inhalation powder capsules with handihaler (de pharmaceuticals)
35011	antimuscarinic bronchodilators	tiotropium bromide monohydrate	tiotropium bromide 18microgram inhalation powder capsules
49227	antimuscarinic bronchodilators		aclidinium bromide 375micrograms/dose dry powder inhaler
50103	antimuscarinic bronchodilators		spiriva 18microgram inhalation powder capsules with handihaler (waymade healthcare plc)
7908	antimuscarinics	glycopyrronium bromide	robinul 2mg tablet (wyeth pharmaceuticals)
59638	antimuscarinic bronchodilators		spiriva 18microgram inhalation powder capsules with handihaler (sigma pharmaceuticals plc)
51967	antimuscarinic bronchodilators	tiotropium bromide	spiriva 18microgram inhalation powder capsules (mawdsley-brooks & company ltd)
53982	antimuscarinic bronchodilators		seebri breezhaler 44microgram inhalation powder capsules with device (novartis pharmaceuticals uk ltd)
6050	antimuscarinic bronchodilators	tiotropium bromide	spiriva 18 microgram capsule (boehringer ingelheim ltd)
7597	other ulcer healing drugs	glycopyrronium bromide	glycopyrronium bromide 2mg tablets
49228	antimuscarinic bronchodilators		eklira 322micrograms/dose genuair (almirall ltd)
59173	other ulcer healing drugs	glycopyrronium bromide	glycopyrronium bromide 200micrograms/5ml oral suspension

Product code	BNF header	Drug substance	Drug product
36864	antimuscarinic bronchodilators	tiotropium bromide	tiotropium bromide 2.5micrograms/dose solution for inhalation cartridge with device cfc free
55911	other ulcer healing drugs	glycopyrronium bromide	glycopyrronium bromide 500micrograms/5ml oral solution
34995	antimuscarinic bronchodilators	tiotropium bromide	spiriva 18microgram inhalation powder capsules with handihaler (boehringer ingelheim ltd)
35000	antimuscarinic bronchodilators	tiotropium bromide	spiriva 18microgram inhalation powder capsules (boehringer ingelheim ltd)
29138	other ulcer healing drugs	glycopyrronium bromide	glycopyrronium bromide 1mg/5ml oral solution
47269	other ulcer healing drugs	glycopyrronium bromide	glycopyrronium bromide 1mg/5ml oral suspension
54151	other ulcer healing drugs	glycopyrronium bromide	glycopyrronium bromide 600micrograms/5ml oral suspension
55795	other ulcer healing drugs	glycopyrronium bromide	glycopyrronium bromide 500micrograms/5ml oral suspension
38377	other ulcer healing drugs	glycopyrronium bromide	glycopyrronium bromide 2mg/5ml oral solution
7218	other ulcer healing drugs	glycopyrronium bromide	glycopyrronium bromide 1mg tablets
36869	antimuscarinic bronchodilators	tiotropium bromide	spiriva respimat 2.5micrograms/dose solution for inhalation cartridge with device (boehringer ingelheim ltd)
62109	antimuscarinic bronchodilators		umeclidinium bromide 65micrograms/dose dry powder inhaler
50292	antimuscarinic bronchodilators		spiriva 18microgram inhalation powder capsules (sigma pharmaceuticals plc)
55794	other ulcer healing drugs	glycopyrronium bromide	glycopyrronium bromide 5mg/5ml oral suspension
50047	other ulcer healing drugs	glycopyrronium bromide	glycopyrronium bromide 5mg/5ml oral solution
56262	other ulcer healing drugs	glycopyrronium bromide	glycopyrronium bromide 200micrograms/5ml oral solution
61879	antimuscarinic bronchodilators		incruse ellipta 55micrograms/dose dry powder inhaler (glaxosmithkline uk ltd)
53761	antimuscarinic bronchodilators		glycopyrronium bromide 55microgram inhalation powder capsules with device
746	antimuscarinic bronchodilators	tiotropium bromide	tiotropium 18 microgram capsule
38538	other ulcer healing drugs	glycopyrronium bromide	glycopyrronium bromide 2mg/5ml oral suspension
46214	antimuscarinics	glycopyrronium bromide	glycopyrronium bromide 5mg/5ml oral solution

Product code	BNF header	Drug substance	Drug product
61582	antimuscarinic bronchodilators	tiotropium bromide	spiriva respimat 2.5micrograms/dose solution for inhalation cartridge with device (waymade healthcare plc)

LABA codes

Product code	BNF header	Drug substance	Drug product
45610	selective beta 2 agonists	indacaterol maleate	indacaterol 300microgram inhalation powder capsules with device
7270	selective beta 2 agonists	salmeterol xinafoate	salmeterol 25micrograms/dose inhaler cfc free
10968	selective beta 2 agonists	formoterol fumarate dihydrate	foradil 12microgram inhalation powder capsules with device (novartis pharmaceuticals uk ltd)
549	unknown	salmeterol xinafoate	serevent 25micrograms/dose inhaler (glaxosmithkline uk ltd)
7133	selective beta 2 agonists	formoterol fumarate dihydrate	formoterol 12micrograms/dose dry powder inhaler
54742	selective beta 2 agonists	salmeterol xinafoate	salmeterol 25micrograms/dose inhaler cfc free (a a h pharmaceuticals ltd)
719	selective beta 2 agonists	salmeterol xinafoate	salmeterol 50micrograms/dose dry powder inhaler
57694	selective beta 2 agonists	salmeterol xinafoate	vertine 25micrograms/dose inhaler cfc free (teva uk ltd)
26829	selective beta 2 agonists	tulobuterol	brelomax 2mg tablet (abbott laboratories ltd)
19799	selective beta 2 agonists	tulobuterol	tulobuterol 2mg
56482	selective beta 2 agonists	formoterol fumarate dihydrate	oxis 12 turbohaler (waymade healthcare plc)
25784	selective beta 2 agonists	formoterol fumarate dihydrate	atimos modulite 12micrograms/dose inhaler (chiesi ltd)
47638	selective beta 2 agonists	salmeterol xinafoate	neovent 25micrograms/dose inhaler cfc free (kent pharmaceuticals ltd)
10672	peripheral vasodilators and related drugs	moxisylyte hydrochloride	opilon 40mg tablet (concord pharmaceuticals ltd)
50051	selective beta 2 agonists	salmeterol xinafoate	serevent 25micrograms/dose evohaler (waymade healthcare plc)
35725	selective beta 2 agonists	formoterol fumarate dihydrate	formoterol easyhaler 12micrograms/dose dry powder inhaler (orion pharma (uk) ltd)

Product code	BNF header	Drug substance	Drug product
2224	selective beta 2 agonists	salmeterol xinafoate	serevent 50micrograms/dose accuhaler (glaxosmithkline uk ltd)
43893	selective beta 2 agonists	indacaterol maleate	onbrez breezhaler 150microgram inhalation powder capsules with device (novartis pharmaceuticals uk ltd)
6526	selective beta 2 agonists	formoterol fumarate dihydrate	formoterol 12microgram inhalation powder capsules with device
43738	selective beta 2 agonists	indacaterol maleate	indacaterol 150microgram inhalation powder capsules with device
35825	selective beta 2 agonists	salmeterol xinafoate	serevent 50microgram disks (glaxosmithkline uk ltd)
43764	peripheral vasodilators and related drugs	moxisylyte hydrochloride	opilon 40mg tablets (archimedes pharma uk ltd)
56478	selective beta 2 agonists	salmeterol xinafoate	serevent 50micrograms/dose accuhaler (de pharmaceuticals)
42103	selective beta 2 agonists	tulobuterol	tulobuterol 1mg/5ml sugar free syrup
57544	selective beta 2 agonists	salmeterol xinafoate	serevent 50micrograms/dose accuhaler (waymade healthcare plc)
3297	selective beta 2 agonists	salmeterol xinafoate	salmeterol 50micrograms disc
57558	selective beta 2 agonists	formoterol fumarate dihydrate	oxis 6 turbohaler (lexon (uk) ltd)
9711	selective beta 2 agonists	formoterol fumarate dihydrate	formoterol 6micrograms/dose dry powder inhaler
35542	selective beta 2 agonists	salmeterol xinafoate	salmeterol 50microgram inhalation powder blisters with device
465	unknown	salmeterol xinafoate	salmeterol 25micrograms/dose inhaler
44064	selective beta 2 agonists	indacaterol maleate	onbrez breezhaler 300microgram inhalation powder capsules with device (novartis pharmaceuticals uk ltd)
35165	selective beta 2 agonists	salmeterol xinafoate	serevent 50microgram disks with diskhaler (glaxosmithkline uk ltd)
14306	selective beta 2 agonists	formoterol fumarate dihydrate	formoterol 12micrograms/dose inhaler cfc free
1974	selective beta 2 agonists	formoterol fumarate dihydrate	oxis 12 turbohaler (astrazeneca uk ltd)
7268	selective beta 2 agonists	salmeterol xinafoate	serevent 25micrograms/dose evohaler (glaxosmithkline uk ltd)
8365	peripheral vasodilators and related drugs	moxisylyte hydrochloride	moxisylyte 40mg tablets
35503	selective beta 2 agonists	salmeterol xinafoate	salmeterol 50microgram inhalation powder blisters
1975	selective beta 2 agonists	formoterol fumarate dihydrate	oxis 6 turbohaler (astrazeneca uk ltd)
22663	selective beta 2 agonists	tulobuterol	respacal 2mg tablet (ucb pharma ltd)

Product code	BNF header	Drug substance	Drug product
910	selective beta 2 agonists	salmeterol xinafoate	serevent diskhaler 50microgram inhalation powder (glaxo wellcome uk ltd)

ICS codes

Product code	BNF header	Drug substance	Drug product
54399	corticosteroids (for respiratory conditions)	beclometasone dipropionate	qvar 100 autohaler (sigma pharmaceuticals plc)
959	unknown	budesonide	budesonide 50micrograms/dose inhaler
2951	corticosteroids (for respiratory conditions)	fluticasone propionate	fluticasone 250microgram/actuation pressurised inhalation
50129	corticosteroids (for respiratory conditions)	beclometasone dipropionate	qvar 100micrograms/dose easi-breathe inhaler (de pharmaceuticals)
2229	corticosteroids (for respiratory conditions)	beclometasone dipropionate	becodisks 100microgram disc (allen & hanburys ltd)
3989	corticosteroids (for respiratory conditions)	fluticasone propionate	flixotide 100microgram disc (allen & hanburys ltd)
5551	corticosteroids (for respiratory conditions)	fluticasone propionate	flixotide 0.5mg/2ml nebules (glaxosmithkline uk ltd)
34794	corticosteroids (for respiratory conditions)	beclometasone dipropionate	beclometasone 200micrograms/dose inhaler (a a h pharmaceuticals ltd)
41269	corticosteroids (for respiratory conditions)	beclometasone dipropionate	beclometasone 400 cyclocaps (teva uk ltd)
29325	corticosteroids (for respiratory conditions)	beclometasone dipropionate	beclometasone 250micrograms/dose inhaler (generics (uk) ltd)
4499	corticosteroids (for respiratory conditions)	beclometasone dipropionate	aerobec 250microgram/actuation pressurised inhalation (meda pharmaceuticals ltd)
57589	corticosteroids (for respiratory conditions)	beclometasone dipropionate	becloforte 250micrograms/dose inhaler (dowelhurst ltd)
32874	corticosteroids (for respiratory conditions)	beclometasone dipropionate	beclometasone 50microgram/actuation inhalation powder (actavis uk ltd)
2159	corticosteroids (for respiratory conditions)	beclometasone dipropionate	aerobec 50 autohaler (meda pharmaceuticals ltd)
14590	corticosteroids (for respiratory conditions)	beclometasone dipropionate	asmabec 250microgram/actuation spacehaler (celltech pharma europe ltd)
1725	corticosteroids (for respiratory conditions)	beclometasone dipropionate	beclazone 50 easi-breathe inhaler (teva uk ltd)
908	corticosteroids (for respiratory conditions)	budesonide	pulmicort 400 turbohaler (astrazeneca uk ltd)
35288	corticosteroids (for respiratory conditions)	beclometasone dipropionate	beclometasone 400microgram inhalation powder blisters
49367	corticosteroids (for respiratory conditions)	beclometasone dipropionate	clenil modulite 50micrograms/dose inhaler (mawdsley-brooks & company ltd)
35107	corticosteroids (for respiratory conditions)	beclometasone dipropionate	beclometasone 400microgram inhalation powder blisters with device

Product code	BNF header	Drug substance	Drug product
1676	corticosteroids (for respiratory conditions)	fluticasone propionate	flixotide 125microgram/actuation inhalation powder (allen & hanburys ltd)
49772	corticosteroids (for respiratory conditions)	fluticasone propionate	fluticasone 250micrograms/dose evohaler (sigma pharmaceuticals plc)
48088	unknown	budesonide	budenofalk 9mg gastro-resistant granules sachets (dr. falk pharma uk ltd)
33258	corticosteroids (for respiratory conditions)	beclometasone dipropionate	beclometasone 250micrograms/dose inhaler (a a h pharmaceuticals ltd)
13037	corticosteroids (for respiratory conditions)	beclometasone dipropionate	pulvinal beclometasone dipropionate 200micrograms/dose dry powder inhaler (chiesi ltd)
19031	corticosteroids (for respiratory conditions)	beclometasone dipropionate	bdp 100microgram/actuation spacehaler (celltech pharma europe ltd)
16158	corticosteroids (for respiratory conditions)	beclometasone dipropionate	clenil modulite 50micrograms/dose inhaler (chiesi ltd)
2124	unknown		pulmicort refil 200 mcg inh
4759	corticosteroids (for respiratory conditions)	beclometasone dipropionate	beclometasone 100microgram inhalation powder capsules
3753	unknown		flixotide diskhaler-community pack 250 mcg
49711	corticosteroids (for respiratory conditions)	budesonide	pulmicort 200micrograms/dose inhaler (astrazeneca uk ltd)
15326	corticosteroids (for respiratory conditions)	beclometasone dipropionate	beclometasone 100micrograms/dose inhaler cfc free
2335	corticosteroids (for respiratory conditions)	beclometasone dipropionate	qvar 100 inhaler (teva uk ltd)
5580	corticosteroids (for respiratory conditions)	fluticasone propionate	flixotide accuhaler 50 50microgram/inhalation inhalation powder (allen & hanburys ltd)
51681	corticosteroids (for respiratory conditions)	beclometasone dipropionate	qvar 100 inhaler (sigma pharmaceuticals plc)
8433	corticosteroids (for respiratory conditions)	budesonide	budesonide 100micrograms/actuation inhaler
9577	corticosteroids (for respiratory conditions)	beclometasone dipropionate	asmabec 50 clickhaler (focus pharmaceuticals ltd)
2723	corticosteroids (for respiratory conditions)	fluticasone propionate	fluticasone 25micrograms/dose inhaler
23675	unknown		pulmicort l.s. refil
35638	corticosteroids (for respiratory conditions)	fluticasone propionate	fluticasone propionate 100microgram inhalation powder blisters with device
9571	corticosteroids (for respiratory conditions)	beclometasone dipropionate	beclometasone 250micrograms/actuation vortex inhaler
51234	corticosteroids (for respiratory conditions)	beclometasone dipropionate	qvar 100 inhaler (waymade healthcare plc)

Product code	BNF header	Drug substance	Drug product
60946	corticosteroids (in chronic bowel disorders)	budesonide	entocort cr 3mg capsules (waymade healthcare plc)
5223	corticosteroids (for respiratory conditions)	fluticasone propionate	fluticasone 50micrograms/dose inhaler cfc free
53057	corticosteroids (for respiratory conditions)	fluticasone propionate	flixotide 50micrograms/dose evohaler (lexon (uk) ltd)
13290	corticosteroids (for respiratory conditions)	beclometasone dipropionate	clenil modulite 100micrograms/dose inhaler (chiesi ltd)
42928	corticosteroids (for respiratory conditions)	fluticasone propionate	flixotide 100micrograms/dose accuhaler (glaxosmithkline uk ltd)
14736	corticosteroids (for respiratory conditions)	beclometasone dipropionate	pulvinal beclometasone dipropionate 400micrograms/dose dry powder inhaler (chiesi ltd)
52806	corticosteroids (for respiratory conditions)	beclometasone dipropionate	qvar 100 autohaler (lexon (uk) ltd)
57525	corticosteroids (for respiratory conditions)	fluticasone propionate	flixotide 250micrograms/dose accuhaler (stephar (u.k.) ltd)
33849	corticosteroids (for respiratory conditions)	beclometasone dipropionate	beclometasone 100microgram/actuation inhalation powder (neo laboratories ltd)
52732	corticosteroids (for respiratory conditions)	budesonide	pulmicort 0.5mg respules (necessity supplies ltd)
35611	corticosteroids (for respiratory conditions)	fluticasone propionate	flixotide 250microgram disks (glaxosmithkline uk ltd)
8111	corticosteroids (for respiratory conditions)	beclometasone dipropionate	becloforte vm 250microgram/actuation vm pack (allen & hanburys ltd)
39879	unknown	budesonide	budesonide 200micrograms/dose inhaler cfc free
1242	corticosteroids (for respiratory conditions)	beclometasone dipropionate	beclometasone 250micrograms/dose inhaler
26665	unknown		pulmicort complete
37447	corticosteroids (for respiratory conditions)	fluticasone propionate	fluticasone propionate 50microgram inhalation powder blisters
5718	corticosteroids (for respiratory conditions)	fluticasone propionate	flixotide 125micrograms/dose evohaler (glaxosmithkline uk ltd)
7653	corticosteroids (for respiratory conditions)	beclometasone dipropionate	beclometasone 400microgram inhalation powder capsules
28640	corticosteroids (for respiratory conditions)	beclometasone dipropionate	beclometasone 100microgram/actuation inhalation powder (actavis uk ltd)
4365	corticosteroids (for respiratory conditions)	beclometasone dipropionate	beclometasone 100micrograms disc
34739	corticosteroids (for respiratory conditions)	beclometasone dipropionate	beclometasone 50micrograms/dose inhaler (teva uk ltd)
8635	corticosteroids (for respiratory conditions)	fluticasone propionate	flixotide 50microgram disc (allen & hanburys ltd)

Product code	BNF header	Drug substance	Drug product
57579	corticosteroids (for respiratory conditions)	fluticasone propionate	flixotide 50micrograms/dose accuhaler (de pharmaceuticals)
4413	corticosteroids (for respiratory conditions)	beclometasone dipropionate	qvar 100 autohaler (teva uk ltd)
37203	corticosteroids (in chronic bowel disorders)	beclometasone dipropionate	beclometasone 5mg gastro-resistant modified-release tablets
4132	corticosteroids (for respiratory conditions)	fluticasone propionate	fluticasone 125microgram/actuation pressurised inhalation
3018	corticosteroids (for respiratory conditions)	beclometasone dipropionate	beclometasone 50micrograms/dose inhaler
9233	corticosteroids (for respiratory conditions)	beclometasone dipropionate	beclometasone 200microgram inhalation powder capsules
2160	corticosteroids (for respiratory conditions)	beclometasone dipropionate	beclometasone 50micrograms/dose breath actuated inhaler
2148	corticosteroids (for respiratory conditions)	beclometasone dipropionate	beclometasone 400microgram disc
34919	corticosteroids (for respiratory conditions)	beclometasone dipropionate	beclometasone 50micrograms/dose inhaler (a a h pharmaceuticals ltd)
2092	corticosteroids (for respiratory conditions)	budesonide	budesonide 200micrograms/dose dry powder inhaler
21005	corticosteroids (for respiratory conditions)	beclometasone dipropionate	beclometasone 250micrograms/dose inhaler cfc free
10090	corticosteroids (for respiratory conditions)	beclometasone dipropionate	beclometasone 50micrograms/actuation extrafine particle cfc free inhaler
1551	corticosteroids (for respiratory conditions)	beclometasone dipropionate	beclazone 250 inhaler (teva uk ltd)
39067	corticosteroids (in chronic bowel disorders)	beclometasone dipropionate	clipper 5mg gastro-resistant modified-release tablets (chiesi ltd)
51415	corticosteroids (for respiratory conditions)	beclometasone dipropionate	qvar 50 inhaler (mawdsley-brooks & company ltd)
35106	corticosteroids (for respiratory conditions)	beclometasone dipropionate	becodisks 100microgram with diskhaler (glaxosmithkline uk ltd)
35299	corticosteroids (for respiratory conditions)	beclometasone dipropionate	becodisks 400microgram (glaxosmithkline uk ltd)
54207	corticosteroids (for respiratory conditions)	beclometasone dipropionate	qvar 50 inhaler (de pharmaceuticals)
3927	corticosteroids (for respiratory conditions)	beclometasone dipropionate	filair 100 inhaler (meda pharmaceuticals ltd)
17670	corticosteroids (for respiratory conditions)	budesonide	easyhaler budesonide 100micrograms/dose dry powder inhaler (orion pharma (uk) ltd)
883	corticosteroids (for respiratory conditions)	beclometasone dipropionate	becodisks 200microgram disc (allen & hanburys ltd)
8450	unknown		flixotide diskhaler-community pack 50 mcg
3289	corticosteroids (for respiratory conditions)	fluticasone propionate	flixotide 25micrograms/dose inhaler (glaxosmithkline uk ltd)

Product code	BNF header	Drug substance	Drug product
53480	corticosteroids (for respiratory conditions)	beclometasone dipropionate	qvar 100 autohaler (stephar (u.k.) ltd)
39102	unknown	budesonide	budesonide 100micrograms/dose inhaler cfc free
20763	unknown		becloforte
35374	corticosteroids (for respiratory conditions)	fluticasone propionate	flixotide 500microgram disks (glaxosmithkline uk ltd)
39200	corticosteroids (for respiratory conditions)	beclometasone dipropionate	aerobec forte 250 autohaler (meda pharmaceuticals ltd)
9599	corticosteroids (for respiratory conditions)	beclometasone dipropionate	beclazone 50microgram/actuation inhalation powder (actavis uk ltd)
5804	corticosteroids (for respiratory conditions)	beclometasone dipropionate	beclometasone 250micrograms/dose dry powder inhaler
47943	corticosteroids (for respiratory conditions)	beclometasone dipropionate	beclazone easi-breathe (roi) 100microgram/actuation pressurised inhalation (ivax pharmaceuticals ireland)
5683	corticosteroids (for respiratory conditions)	fluticasone propionate	flixotide 250micrograms/dose evohaler (glaxosmithkline uk ltd)
48340	corticosteroids (for respiratory conditions)	beclometasone dipropionate	clenil modulite 100micrograms/dose inhaler (mawdsley-brooks & company ltd)
1885	corticosteroids (for respiratory conditions)	beclometasone dipropionate	beclazone 200 inhaler (teva uk ltd)
911	corticosteroids (for respiratory conditions)	fluticasone propionate	flixotide accuhaler 250 250microgram/inhalation inhalation powder (allen & hanburys ltd)
6095	corticosteroids (in chronic bowel disorders)	budesonide	budesonide 3mg gastro-resistant capsules
28073	corticosteroids (for respiratory conditions)	beclometasone dipropionate	beclometasone 250microgram/actuation pressurised inhalation (approved prescription services ltd)
4131	corticosteroids (for respiratory conditions)	fluticasone propionate	fluticasone 100microgram disc
43074	corticosteroids (for respiratory conditions)	fluticasone propionate	flixotide 500micrograms/dose accuhaler (glaxosmithkline uk ltd)
35905	corticosteroids (for respiratory conditions)	fluticasone propionate	fluticasone propionate 250microgram inhalation powder blisters
27583	unknown		pulmicort
17654	corticosteroids (for respiratory conditions)	beclometasone dipropionate	easyhaler beclometasone 200micrograms/dose dry powder inhaler (orion pharma (uk) ltd)
4545	corticosteroids (for respiratory conditions)	budesonide	pulmicort ls 50microgram refill canister (astrazeneca uk ltd)
1412	corticosteroids (for respiratory conditions)	fluticasone propionate	flixotide 250microgram/actuation inhalation powder (allen & hanburys ltd)

Product code	BNF header	Drug substance	Drug product
3363	corticosteroids (for respiratory conditions)	beclometasone dipropionate	becloforte 400microgram disks with diskhaler (glaxosmithkline uk ltd)
36462	corticosteroids (for respiratory conditions)	fluticasone propionate	fluticasone propionate 500microgram inhalation powder blisters
56471	corticosteroids (for respiratory conditions)	beclometasone dipropionate	becodisks 200microgram (mawdsley-brooks & company ltd)
14294	corticosteroids (for respiratory conditions)	beclometasone dipropionate	qvar 50micrograms/dose easi-breathe inhaler (teva uk ltd)
3570	corticosteroids (for respiratory conditions)	budesonide	budesonide 200micrograms/actuation refill canister
9164	corticosteroids (for respiratory conditions)	fluticasone propionate	fluticasone propionate 50micrograms/dose dry powder inhaler
27188	corticosteroids (for respiratory conditions)	budesonide	easyhaler budesonide 200micrograms/dose dry powder inhaler (orion pharma (uk) ltd)
39099	unknown	budesonide	pulmicort 100micrograms/dose inhaler cfc free (astrazeneca uk ltd)
4926	corticosteroids (for respiratory conditions)	fluticasone propionate	flixotide accuhaler 100 100microgram/inhalation inhalation powder (allen & hanburys ltd)
1642	corticosteroids (for respiratory conditions)	budesonide	budesonide 400micrograms/dose dry powder inhaler
34859	corticosteroids (for respiratory conditions)	beclometasone dipropionate	beclometasone 250microgram/actuation inhalation powder (neo laboratories ltd)
16054	corticosteroids (for respiratory conditions)	budesonide	budesonide 200micrograms/actuation breath actuated powder inhaler
960	corticosteroids (for respiratory conditions)	budesonide	pulmicort 100 turbohaler (astrazeneca uk ltd)
51480	corticosteroids (for respiratory conditions)	beclometasone dipropionate	qvar 100 autohaler (de pharmaceuticals)
7891	corticosteroids (for respiratory conditions)	fluticasone propionate	fluticasone 500microgram disc
56462	corticosteroids (for respiratory conditions)	beclometasone dipropionate	becodisks 400microgram (waymade healthcare plc)
7638	corticosteroids (for respiratory conditions)	fluticasone propionate	fluticasone 250microgram disc
15706	corticosteroids (for respiratory conditions)	beclometasone dipropionate	beclometasone 100 micrograms/actuation vortex inhaler
27915	unknown		fluticasone prop disk refill
2893	corticosteroids (for respiratory conditions)	beclometasone dipropionate	beclometasone 200micrograms disc
5885	corticosteroids (for respiratory conditions)	fluticasone propionate	fluticasone propionate 100micrograms/dose dry powder inhaler

Product code	BNF header	Drug substance	Drug product
3898	corticosteroids (in chronic bowel disorders)	budesonide	budesonide 3mg gastro-resistant modified-release capsules
3546	corticosteroids (for respiratory conditions)	beclometasone dipropionate	qvar 50 inhaler (teva uk ltd)
30210	corticosteroids (for respiratory conditions)	beclometasone dipropionate	beclometasone 250micrograms/dose inhaler (teva uk ltd)
1552	corticosteroids (for respiratory conditions)	beclometasone dipropionate	becloforte easi-breathe 250microgram/actuation pressurised inhalation (allen & hanburys ltd)
5822	corticosteroids (for respiratory conditions)	fluticasone propionate	fluticasone 250micrograms/dose inhaler cfc free
35430	corticosteroids (for respiratory conditions)	beclometasone dipropionate	becodisks 200microgram with diskhaler (glaxosmithkline uk ltd)
42994	corticosteroids (for respiratory conditions)	fluticasone propionate	flixotide 250micrograms/dose accuhaler (glaxosmithkline uk ltd)
36290	corticosteroids (for respiratory conditions)	fluticasone propionate	flixotide 50microgram disks with diskhaler (glaxosmithkline uk ltd)
24898	corticosteroids (for respiratory conditions)	beclometasone dipropionate	spacehaler bdp 100microgram/actuation spacehaler (celltech pharma europe ltd)
35700	corticosteroids (for respiratory conditions)	fluticasone propionate	fluticasone propionate 500microgram inhalation powder blisters with device
3188	unknown		pulmicort complete 50 mcg inh
7788	corticosteroids (for respiratory conditions)	budesonide	budesonide 100micrograms/dose dry powder inhaler
35293	corticosteroids (for respiratory conditions)	beclometasone dipropionate	beclometasone 200microgram inhalation powder blisters with device
8251	unknown		pulmicort refil 50 mg inh
1259	corticosteroids (for respiratory conditions)	beclometasone dipropionate	beclometasone 200micrograms/dose inhaler
16525	corticosteroids (in chronic bowel disorders)	budesonide	budenofalk 3mg gastro-resistant capsules (dr. falk pharma uk ltd)
1680	corticosteroids (for respiratory conditions)	budesonide	pulmicort ls 50micrograms/dose inhaler (astrazeneca uk ltd)
10254	corticosteroids (for respiratory conditions)	mometasone furoate	mometasone 400micrograms/dose dry powder inhaler
20812	unknown		pulmicort refill
14524	corticosteroids (for respiratory conditions)	beclometasone dipropionate	bdp 250microgram/actuation spacehaler (celltech pharma europe ltd)
2992	corticosteroids (for respiratory conditions)	beclometasone dipropionate	beclazone 50 inhaler (teva uk ltd)
7602	corticosteroids (for respiratory conditions)	fluticasone propionate	fluticasone 50microgram disc

Product code	BNF header	Drug substance	Drug product
16305	corticosteroids (for respiratory conditions)	fluticasone propionate	flixotide 2mg/2ml nebules (glaxosmithkline uk ltd)
14757	corticosteroids (for respiratory conditions)	beclometasone dipropionate	pulvinal beclometasone dipropionate 100micrograms/dose dry powder inhaler (chiesi ltd)
909	unknown	budesonide	budesonide 200micrograms/dose inhaler
1959	corticosteroids (for respiratory conditions)	budesonide	pulmicort 0.5mg respules (astrazeneca uk ltd)
1861	corticosteroids (for respiratory conditions)	beclometasone dipropionate	aerobec 100 autohaler (meda pharmaceuticals ltd)
16018	corticosteroids (for respiratory conditions)	mometasone furoate	mometasone 200micrograms/dose dry powder inhaler
35631	corticosteroids (for respiratory conditions)	budesonide	budelin novolizer 200micrograms/dose inhalation powder (meda pharmaceuticals ltd)
60937	corticosteroids (for respiratory conditions)	budesonide	pulmicort 200 turbohaler (dowelhurst ltd)
50037	corticosteroids (for respiratory conditions)	budesonide	pulmicort 0.5mg respules (waymade healthcare plc)
19389	corticosteroids (for respiratory conditions)	beclometasone dipropionate	asmabec 50microgram/actuation spacehaler (celltech pharma europe ltd)
2282	corticosteroids (for respiratory conditions)	fluticasone propionate	fluticasone propionate 500micrograms/dose dry powder inhaler
18848	corticosteroids (for respiratory conditions)	beclometasone dipropionate	qvar 100micrograms/dose easi-breathe inhaler (teva uk ltd)
2440	corticosteroids (for respiratory conditions)	fluticasone propionate	flixotide accuhaler 500 500microgram/inhalation inhalation powder (allen & hanburys ltd)
23741	corticosteroids (for respiratory conditions)	budesonide	novolizer budesonide 200microgram/actuation pressurised inhalation (meda pharmaceuticals ltd)
3988	unknown		flixotide diskhaler-community pack 100 mcg
35724	corticosteroids (for respiratory conditions)	budesonide	budelin novolizer 200micrograms/dose inhalation powder refill (meda pharmaceuticals ltd)
1380	corticosteroids (in chronic bowel disorders)	budesonide	entocort cr 3mg capsules (astrazeneca uk ltd)
56144	corticosteroids (in chronic bowel disorders)	budesonide	budenofalk 9mg gastro-resistant granules sachets (dr. falk pharma uk ltd)
56475	corticosteroids (for respiratory conditions)	fluticasone propionate	flixotide 50micrograms/dose accuhaler (sigma pharmaceuticals plc)
40057	corticosteroids (for respiratory conditions)	budesonide	pulmicort 200micrograms/dose inhaler cfc free (astrazeneca uk ltd)
1236	corticosteroids (for respiratory conditions)	beclometasone dipropionate	becloforte 250micrograms/dose inhaler (glaxosmithkline uk ltd)

Product code	BNF header	Drug substance	Drug product
11497	corticosteroids (for respiratory conditions)	beclometasone dipropionate	beclometasone 400micrograms/dose dry powder inhaler
35408	corticosteroids (for respiratory conditions)	beclometasone dipropionate	becodisks 100microgram (glaxosmithkline uk ltd)
34315	corticosteroids (for respiratory conditions)	beclometasone dipropionate	beclometasone 250microgram/actuation inhalation powder (actavis uk ltd)
956	corticosteroids (for respiratory conditions)	budesonide	pulmicort 200 turbohaler (astrazeneca uk ltd)
18394	corticosteroids (for respiratory conditions)	beclometasone dipropionate	bdp 50microgram/actuation spacehaler (celltech pharma europe ltd)
14321	corticosteroids (for respiratory conditions)	beclometasone dipropionate	beclometasone 200micrograms/dose inhaler cfc free
11732	corticosteroids (for respiratory conditions)	beclometasone dipropionate	beclometasone 50micrograms/dose breath actuated inhaler cfc free
3993	corticosteroids (for respiratory conditions)	beclometasone dipropionate	filair forte 250micrograms/dose inhaler (meda pharmaceuticals ltd)
35225	corticosteroids (for respiratory conditions)	fluticasone propionate	flixotide 100microgram disks with diskhaler (glaxosmithkline uk ltd)
5521	corticosteroids (for respiratory conditions)	beclometasone dipropionate	beclometasone 200micrograms/dose dry powder inhaler
4601	corticosteroids (for respiratory conditions)	beclometasone dipropionate	asmabec 100 clickhaler (focus pharmaceuticals ltd)
16148	corticosteroids (for respiratory conditions)	beclometasone dipropionate	clenil modulite 250micrograms/dose inhaler (chiesi ltd)
2892	corticosteroids (for respiratory conditions)	beclometasone dipropionate	becloforte 400microgram disks (glaxosmithkline uk ltd)
35071	corticosteroids (for respiratory conditions)	beclometasone dipropionate	becodisks 200microgram (glaxosmithkline uk ltd)
35510	corticosteroids (for respiratory conditions)	budesonide	budesonide 200micrograms/dose dry powder inhalation cartridge with device
14567	corticosteroids (for respiratory conditions)	beclometasone dipropionate	asmabec 250 clickhaler (focus pharmaceuticals ltd)
56498	corticosteroids (for respiratory conditions)	budesonide	pulmicort 200 turbohaler (waymade healthcare plc)
24660	unknown		betamethasone valerate
51997	corticosteroids (in chronic bowel disorders)	budesonide	budesonide 9mg gastro-resistant granules sachets
1734	corticosteroids (for respiratory conditions)	beclometasone dipropionate	beclometasone 100micrograms/dose breath actuated inhaler
36090	corticosteroids (for respiratory conditions)	fluticasone propionate	flixotide 100microgram disks (glaxosmithkline uk ltd)
5975	corticosteroids (for respiratory conditions)	fluticasone propionate	fluticasone 125micrograms/dose inhaler cfc free
19401	corticosteroids (for respiratory conditions)	beclometasone dipropionate	beclometasone 250micrograms/actuation inhaler and compact spacer

Product code	BNF header	Drug substance	Drug product
895	corticosteroids (for respiratory conditions)	beclometasone dipropionate	beclazone 100 easi-breathe inhaler (teva uk ltd)
51815	corticosteroids (for respiratory conditions)	fluticasone propionate	flixotide 250micrograms/dose evohaler (waymade healthcare plc)
36021	corticosteroids (for respiratory conditions)	fluticasone propionate	fluticasone propionate 50microgram inhalation powder blisters with device
11198	corticosteroids (for respiratory conditions)	beclometasone dipropionate	beclometasons 50 micrograms/actuation vortex inhaler
50287	corticosteroids (for respiratory conditions)	beclometasone dipropionate	qvar 100 inhaler (de pharmaceuticals)
34428	corticosteroids (for respiratory conditions)	beclometasone dipropionate	beclometasone 50microgram/actuation inhalation powder (neo laboratories ltd)
26063	corticosteroids (for respiratory conditions)	beclometasone dipropionate	beclometasone 100micrograms/dose inhaler (teva uk ltd)
35602	corticosteroids (for respiratory conditions)	budesonide	budesonide 200micrograms/dose dry powder inhalation cartridge
30238	corticosteroids (for respiratory conditions)	beclometasone dipropionate	beclometasone 50microgram/actuation pressurised inhalation (approved prescription services ltd)
14700	corticosteroids (for respiratory conditions)	budesonide	budesonide 400micrograms/actuation inhaler
38	corticosteroids (for respiratory conditions)	beclometasone dipropionate	beclometasone 100micrograms/dose inhaler
13815	corticosteroids (for respiratory conditions)	beclometasone dipropionate	beclazone 100microgram/actuation inhalation powder (actavis uk ltd)
947	corticosteroids (for respiratory conditions)	budesonide	budesonide 50micrograms/actuation refill canister
10321	unknown	budesonide	budesonide 400microgram inhalation powder capsules
1426	corticosteroids (for respiratory conditions)	fluticasone propionate	flixotide 500microgram disc (allen & hanburys ltd)
3150	corticosteroids (for respiratory conditions)	beclometasone dipropionate	beclometasone 100micrograms/actuation extrafine particle cfc free inhaler
35113	corticosteroids (for respiratory conditions)	beclometasone dipropionate	beclometasone 200microgram inhalation powder blisters
1424	corticosteroids (for respiratory conditions)	fluticasone propionate	flixotide 250microgram disc (allen & hanburys ltd)
4803	corticosteroids (for respiratory conditions)	beclometasone dipropionate	beclazone 250microgram/actuation inhalation powder (actavis uk ltd)
56493	corticosteroids (for respiratory conditions)	beclometasone dipropionate	qvar 50micrograms/dose easi-breathe inhaler (sigma pharmaceuticals plc)
61664	corticosteroids (for respiratory conditions)	beclometasone dipropionate	clenil modulite 250micrograms/dose inhaler (waymade healthcare plc)

Product code	BNF header	Drug substance	Drug product
35772	corticosteroids (for respiratory conditions)	fluticasone propionate	fluticasone propionate 100microgram inhalation powder blisters
3119	corticosteroids (for respiratory conditions)	beclometasone dipropionate	becloforte integra 250microgram/actuation inhaler with compact spacer (glaxo laboratories ltd)
5522	corticosteroids (for respiratory conditions)	beclometasone dipropionate	beclometasone 100micrograms/dose dry powder inhaler
25204	corticosteroids (for respiratory conditions)	beclometasone dipropionate	beclometasone 100micrograms/dose inhaler (a a h pharmaceuticals ltd)
7724	corticosteroids (for respiratory conditions)	betamethasone valerate	betamethasone valerate 100micrograms/actuation inhaler
3743	corticosteroids (for respiratory conditions)	beclometasone dipropionate	filair 50 inhaler (meda pharmaceuticals ltd)
1956	corticosteroids (for respiratory conditions)	budesonide	pulmicort 1mg respules (astrazeneca uk ltd)
56499	corticosteroids (for respiratory conditions)	fluticasone propionate	flixotide 500micrograms/dose accuhaler (waymade healthcare plc)
2125	corticosteroids (for respiratory conditions)	budesonide	pulmicort 200microgram refill canister (astrazeneca uk ltd)
36401	corticosteroids (for respiratory conditions)	fluticasone propionate	fluticasone propionate 250microgram inhalation powder blisters with device
4688	corticosteroids (for respiratory conditions)	fluticasone propionate	fluticasone 50microgram/actuation pressurised inhalation
3065	corticosteroids (for respiratory conditions)	betamethasone valerate	bextasol inhalation powder (allen & hanburys ltd)
35118	corticosteroids (for respiratory conditions)	beclometasone dipropionate	becodisks 400microgram with diskhaler (glaxosmithkline uk ltd)
1518	corticosteroids (for respiratory conditions)	fluticasone propionate	flixotide 50microgram/actuation inhalation powder (allen & hanburys ltd)
5309	corticosteroids (for respiratory conditions)	fluticasone propionate	flixotide 50micrograms/dose evohaler (glaxosmithkline uk ltd)
47225	unknown	budesonide	budesonide 9mg gastro-resistant granules sachets
30649	corticosteroids (for respiratory conditions)	budesonide	easyhaler budesonide 400micrograms/dose dry powder inhaler (orion pharma (uk) ltd)
35580	corticosteroids (for respiratory conditions)	beclometasone dipropionate	beclometasone 100microgram inhalation powder blisters with device
41412	corticosteroids (for respiratory conditions)	beclometasone dipropionate	beclometasone 400micrograms/actuation inhaler
20825	corticosteroids (for respiratory conditions)	beclometasone dipropionate	spacehaler bdp 250microgram/actuation spacehaler (celltech pharma europe ltd)

Product code	BNF header	Drug substance	Drug product
35461	corticosteroids (for respiratory conditions)	fluticasone propionate	flixotide 250microgram disks with diskhaler (glaxosmithkline uk ltd)
42985	corticosteroids (for respiratory conditions)	fluticasone propionate	flixotide 50micrograms/dose accuhaler (glaxosmithkline uk ltd)
9921	corticosteroids (for respiratory conditions)	beclometasone dipropionate	beclometasone 100micrograms/dose breath actuated inhaler cfc free
56474	corticosteroids (for respiratory conditions)	fluticasone propionate	flixotide 125micrograms/dose evohaler (de pharmaceuticals)
35986	corticosteroids (for respiratory conditions)	fluticasone propionate	flixotide 50microgram disks (glaxosmithkline uk ltd)
11149	glucocorticoid therapy	betamethasone	betnelan 500microgram tablets (focus pharmaceuticals ltd)
1243	corticosteroids (for respiratory conditions)	beclometasone dipropionate	beclazone 250 easi-breathe inhaler (teva uk ltd)
31774	corticosteroids (for respiratory conditions)	beclometasone dipropionate	beclometasone 50micrograms/dose inhaler (generics (uk) ltd)
56477	corticosteroids (for respiratory conditions)	fluticasone propionate	flixotide 100micrograms/dose accuhaler (waymade healthcare plc)
2600	corticosteroids (for respiratory conditions)	beclometasone dipropionate	beclometasone 250micrograms/dose breath actuated inhaler
16151	corticosteroids (for respiratory conditions)	beclometasone dipropionate	clenil modulite 200micrograms/dose inhaler (chiesi ltd)
1100	corticosteroids (for respiratory conditions)	beclometasone dipropionate	beclazone 100 inhaler (teva uk ltd)
1951	corticosteroids (for respiratory conditions)	beclometasone dipropionate	becodisks 400microgram disc (allen & hanburys ltd)
7948	corticosteroids (for respiratory conditions)	fluticasone propionate	fluticasone propionate 250micrograms/dose dry powder inhaler
21482	corticosteroids (for respiratory conditions)	beclometasone dipropionate	beclometasone 100micrograms/dose inhaler (generics (uk) ltd)
9477	corticosteroids (for respiratory conditions)	beclometasone dipropionate	asmabec 100microgram/actuation spacehaler (celltech pharma europe ltd)
27679	corticosteroids (for respiratory conditions)	beclometasone dipropionate	beclometasone 100microgram/actuation pressurised inhalation (approved prescription services ltd)
35652	corticosteroids (for respiratory conditions)	beclometasone dipropionate	beclometasone 100microgram inhalation powder blisters
46157	corticosteroids (for respiratory conditions)	beclometasone dipropionate	beclometasone 200 cyclocaps (teva uk ltd)
56484	corticosteroids (for respiratory conditions)	fluticasone propionate	flixotide 250micrograms/dose accuhaler (waymade healthcare plc)
3442	unknown		pulmicort complete 200 mcg inh

Product code	BNF header	Drug substance	Drug product
28761	corticosteroids (for respiratory conditions)	beclometasone dipropionate	spacehaler bdp 50microgram/actuation spacehaler (celltech pharma europe ltd)
5992	corticosteroids (for respiratory conditions)	beclometasone dipropionate	beclometasone 50micrograms/dose dry powder inhaler
454	corticosteroids (for respiratory conditions)	budesonide	pulmicort 200microgram inhaler (astrazeneca uk ltd)
3220	corticosteroids (for respiratory conditions)	beclometasone dipropionate	qvar 50 autohaler (teva uk ltd)
18537	unknown	budesonide	budesonide 200microgram inhalation powder capsules
57555	corticosteroids (for respiratory conditions)	fluticasone propionate	flixotide 125micrograms/dose evohaler (dowelhurst ltd)
48709	corticosteroids (for respiratory conditions)	beclometasone dipropionate	qvar 100micrograms/dose easi-breathe inhaler (sigma pharmaceuticals plc)
16584	corticosteroids (for respiratory conditions)	beclometasone dipropionate	beclometasone 50micrograms/dose inhaler cfc free
35392	corticosteroids (for respiratory conditions)	fluticasone propionate	flixotide 500microgram disks with diskhaler (glaxosmithkline uk ltd)

FP_SAL codes

Product code	BNF header	Drug substance	Drug product
638	Selective Beta 2 Agonists/Corticosteroids (for Respiratory Conditions)	Fluticasone propionate/Salmeterol xinafoate	Seretide 250 Accuhaler (GlaxoSmithKline UK Ltd)
665	Selective Beta 2 Agonists/Corticosteroids (for Respiratory Conditions)	Salmeterol xinafoate/Fluticasone propionate	Seretide 100 Accuhaler (GlaxoSmithKline UK Ltd)
3666	Selective Beta 2 Agonists/Corticosteroids (for Respiratory Conditions)	Salmeterol xinafoate/Fluticasone propionate	Seretide 500 Accuhaler (GlaxoSmithKline UK Ltd)
5143	Selective Beta 2 Agonists/Corticosteroids (for Respiratory Conditions)	Fluticasone propionate/Salmeterol xinafoate	Seretide 50 Evohaler (GlaxoSmithKline UK Ltd)
5161	Selective Beta 2 Agonists/Corticosteroids (for Respiratory Conditions)	Fluticasone propionate/Salmeterol xinafoate	Seretide 125 Evohaler (GlaxoSmithKline UK Ltd)
5172	Selective Beta 2 Agonists/Corticosteroids (for Respiratory Conditions)	Fluticasone propionate/Salmeterol xinafoate	Seretide 250 Evohaler (GlaxoSmithKline UK Ltd)
5558	Selective Beta 2 Agonists/Corticosteroids (for Respiratory Conditions)	Salmeterol Xinafoate/Fluticasone Propionate	Salmeterol 50micrograms with fluticasone 500micrograms CFC free inhaler
5864	Selective Beta 2 Agonists/Corticosteroids (for Respiratory Conditions)	Salmeterol Xinafoate/Fluticasone Propionate	Salmeterol 25micrograms with fluticasone 250micrograms CFC free inhaler
5942	Selective Beta 2 Agonists/Corticosteroids (for Respiratory Conditions)	Salmeterol Xinafoate/Fluticasone Propionate	Salmeterol 50micrograms with fluticasone 250micrograms CFC free inhaler
6569	Selective Beta 2 Agonists/Corticosteroids (for Respiratory Conditions)	Salmeterol Xinafoate/Fluticasone Propionate	Salmeterol 25micrograms with fluticasone 125micrograms CFC free inhaler
6616	Selective Beta 2 Agonists/Corticosteroids (for Respiratory Conditions)	Salmeterol Xinafoate/Fluticasone Propionate	Salmeterol 25micrograms with fluticasone 50micrograms CFC free inhaler
6938	Selective Beta 2 Agonists/Corticosteroids (for Respiratory Conditions)	Salmeterol Xinafoate/Fluticasone Propionate	Salmeterol 50micrograms with fluticasone 100micrograms dry powder inhaler
11410	Selective Beta 2 Agonists/Corticosteroids (for Respiratory Conditions)	Salmeterol xinafoate/Fluticasone propionate	Fluticasone propionate 500micrograms/dose / Salmeterol 50micrograms/dose dry powder inhaler
11588	Selective Beta 2 Agonists/Corticosteroids (for Respiratory Conditions)	Fluticasone propionate/Salmeterol xinafoate	Fluticasone 125micrograms/dose / Salmeterol 25micrograms/dose inhaler CFC free
11618	Selective Beta 2 Agonists/Corticosteroids (for Respiratory Conditions)	Fluticasone propionate/Salmeterol xinafoate	Fluticasone 250micrograms/dose / Salmeterol 25micrograms/dose inhaler CFC free
12994	Selective Beta 2 Agonists/Corticosteroids (for Respiratory Conditions)	Fluticasone propionate/Salmeterol xinafoate	Fluticasone 50micrograms/dose / Salmeterol 25micrograms/dose inhaler CFC free

Product code	BNF header	Drug substance	Drug product
13040	Selective Beta 2 Agonists/Corticosteroids (for Respiratory Conditions)	Fluticasone propionate/Salmeterol xinafoate	Fluticasone propionate 250micrograms/dose / Salmeterol 50micrograms/dose dry powder inhaler
13273	Selective Beta 2 Agonists/Corticosteroids (for Respiratory Conditions)	Salmeterol xinafoate/Fluticasone propionate	Fluticasone propionate 100micrograms/dose / Salmeterol 50micrograms/dose dry powder inhaler
48739	Selective Beta 2 Agonists/Corticosteroids (for Respiratory Conditions)	Fluticasone propionate/Salmeterol xinafoate	Seretide 250 Evohaler (DE Pharmaceuticals)
49000	Selective Beta 2 Agonists/Corticosteroids (for Respiratory Conditions)	Fluticasone propionate/Salmeterol xinafoate	Seretide 250 Evohaler (Waymade Healthcare Plc)
50560	Selective Beta 2 Agonists/Corticosteroids (for Respiratory Conditions)	Fluticasone propionate/Salmeterol xinafoate	Seretide 250 Accuhaler (Sigma Pharmaceuticals Plc)
50689	Selective Beta 2 Agonists/Corticosteroids (for Respiratory Conditions)	Fluticasone propionate/Formoterol fumarate dihydrate	Flutiform 50micrograms/dose / 5micrograms/dose inhaler (Napp Pharmaceuticals Ltd)
50886	Selective Beta 2 Agonists/Corticosteroids (for Respiratory Conditions)	Fluticasone propionate/Salmeterol xinafoate	Seretide 250 Evohaler (Stephar (U.K.) Ltd)
51027	Selective Beta 2 Agonists/Corticosteroids (for Respiratory Conditions)	Fluticasone propionate/Salmeterol xinafoate	Seretide 125 Evohaler (DE Pharmaceuticals)
51151	Selective Beta 2 Agonists/Corticosteroids (for Respiratory Conditions)	Fluticasone propionate/Salmeterol xinafoate	Seretide 125 Evohaler (Lexon (UK) Ltd)
51270	Selective Beta 2 Agonists/Corticosteroids (for Respiratory Conditions)	Fluticasone propionate/Formoterol fumarate dihydrate	Fluticasone 50micrograms/dose / Formoterol 5micrograms/dose inhaler CFC free
51394	Selective Beta 2 Agonists/Corticosteroids (for Respiratory Conditions)	Salmeterol xinafoate/Fluticasone propionate	Seretide 500 Accuhaler (Waymade Healthcare Plc)
51593	Selective Beta 2 Agonists/Corticosteroids (for Respiratory Conditions)	Salmeterol xinafoate/Fluticasone propionate	Seretide 500 Accuhaler (DE Pharmaceuticals)
51861	Selective Beta 2 Agonists/Corticosteroids (for Respiratory Conditions)	Salmeterol xinafoate/Fluticasone propionate	Seretide 500 Accuhaler (Mawdsley-Brooks & Company Ltd)
51909	Selective Beta 2 Agonists/Corticosteroids (for Respiratory Conditions)	Fluticasone propionate/Salmeterol xinafoate	Seretide 250 Evohaler (Necessity Supplies Ltd)
53230	Selective Beta 2 Agonists/Corticosteroids (for Respiratory Conditions)	Fluticasone propionate/Salmeterol xinafoate	Seretide 250 Accuhaler (DE Pharmaceuticals)
53283	Selective Beta 2 Agonists/Corticosteroids (for Respiratory Conditions)	Salmeterol xinafoate/Fluticasone propionate	Seretide 100 Accuhaler (Waymade Healthcare Plc)
55411	Corticosteroids Used In Nasal Allergy/Antihistamines In Nasal Allergy	Fluticasone propionate/Azelastine hydrochloride	Fluticasone propionate 50micrograms/dose / Azelastine 137micrograms/dose nasal spray
55435	Corticosteroids Used In Nasal Allergy/Antihistamines In Nasal Allergy	Fluticasone propionate/Azelastine hydrochloride	Dymista 137micrograms/dose / 50micrograms/dose nasal spray (Meda Pharmaceuticals Ltd)

Product code	BNF header	Drug substance	Drug product
55677	Selective Beta 2 Agonists/Corticosteroids (for Respiratory Conditions)	Salmeterol xinafoate/Fluticasone propionate	Seretide 500 Accuhaler (Lexon (UK) Ltd)
61280	Selective Beta 2 Agonists/Corticosteroids (for Respiratory Conditions)	Fluticasone propionate/Salmeterol xinafoate	Seretide 250 Accuhaler (Waymade Healthcare Plc)
62126	Selective Beta 2 Agonists/Corticosteroids (for Respiratory Conditions)	Salmeterol xinafoate/Fluticasone propionate	Seretide 100 Accuhaler (DE Pharmaceuticals)
63252	Selective Beta 2 Agonists/Corticosteroids (for Respiratory Conditions)	Fluticasone propionate/Salmeterol xinafoate	Seretide 250 Evohaler (Lexon (UK) Ltd)
63945	Selective Beta 2 Agonists/Corticosteroids (for Respiratory Conditions)	Fluticasone propionate/Salmeterol xinafoate	Seretide 250 Accuhaler (Lexon (UK) Ltd)
64372	Selective Beta 2 Agonists/Corticosteroids (for Respiratory Conditions)	Fluticasone propionate/Salmeterol xinafoate	Sirdupla 25micrograms/dose / 125micrograms/dose inhaler (Mylan Ltd)
64373	Selective Beta 2 Agonists/Corticosteroids (for Respiratory Conditions)	Fluticasone propionate/Salmeterol xinafoate	Sirdupla 25micrograms/dose / 250micrograms/dose inhaler (Mylan Ltd)
65117	Selective Beta 2 Agonists/Corticosteroids (for Respiratory Conditions)	Fluticasone propionate/Salmeterol xinafoate	Seretide 125 Evohaler (Mawdsley-Brooks & Company Ltd)
