# Supplementary material for: Real-world effects of medications for chronic obstructive pulmonary disease: protocol for a UK population-based non-interventional cohort study with validation against randomised trial results
Source: BMJ Open. 2018 Mar 25;8(3):e019475. doi: 10.1136/bmjopen-2017-019475 (PMC5875594; doi:10.1136/bmjopen-2017-019475)
Supplement: Author's manuscript [file bmjopen-2017-019475.draft_revisions.pdf]

BMJ Open

BMJ Open is committed to open peer review. As part of this commitment we make the peer review history of every article we publish publicly available.

When an article is published we post the peer reviewers' comments and the authors' responses online. We also post the versions of the paper that were used during peer review. These are the versions that the peer review comments apply to.

The versions of the paper that follow are the versions that were submitted during the peer review process. They are not the versions of record or the final published versions. They should not be cited or distributed as the published version of this manuscript.

BMJ Open is an open access journal and the full, final, typeset and author-corrected version of record of the manuscript is available on our site with no access controls, subscription charges or pay-per-view fees (http://bmjopen.bmj.com).

If you have any questions on BMJ Open's open peer review process please email info.bmjopen@bmj.com

BMJ Open

Real world effects of medications for chronic obstructive pulmonary disease: protocol for a UK population-based observational cohort study with validation against randomised trial results

Journal:	*BMJ Open*
Manuscript ID	bmjopen-2017-019475
Article Type:	Protocol
Date Submitted by the Author:	05-Sep-2017
Complete List of Authors:	Wing, Kevin; London School of Hygiene and Tropical Medicine,<br>Williamson, Elizabeth; London School of Hygiene and Tropical Medicine,<br>Carpenter, James; London School of Hygiene and Tropical Medicine<br>Wise, Lesley; London School of Hygiene and Tropical Medicine<br>Schneeweiss, Sebastian; Brigham and Womens' Hospital, Harvard Medical School<br>Smeeth, Liam; London School of Hygiene and Tropical Medicine<br>Quint, Jennifer; Imperial College London<br>Douglas, Ian; London School of Hygiene and Tropical Medicine
Keywords:	Chronic airways disease < THORACIC MEDICINE, Epidemiology < THORACIC MEDICINE, CLINICAL PHARMACOLOGY, EPIDEMIOLOGY, PRIMARY CARE, PUBLIC HEALTH

SCHOLARONE™
Manuscripts

TITLE PAGE

Title: Real world effects of medications for chronic obstructive pulmonary disease: protocol for a UK population-based observational cohort study with validation against randomised trial results

Authors:

Kevin Wing[1*]

Elizabeth Williamson[2]

James Carpenter[2]

Lesley Wise[1]

Sebastian Schneeweiss[3,4]

Liam Smeeth[1]

Jennifer Quint[5]

Ian Douglas[1]

[1]Department of Non-communicable Disease Epidemiology, Faculty of Epidemiology and Population Health, London School of Hygiene and Tropical Medicine, London, UK

[2]Department of Medical Statistics, Faculty of Epidemiology and Population Health, London School of Hygiene and Tropical Medicine, London, UK

[3]Department of Epidemiology, Harvard Medical School.

[4]Division of Pharmacoepidemiology and Pharmacoeconomics, Department of Medicine, Brigham & Women's Hospital

[5]National Heart and Lung Institute, Imperial College London, London, United Kingdom

***Submitting and corresponding author:** Kevin Wing (kevin.wing@lshtm.ac.uk), London School of Hygiene and Tropical Medicine, Keppel Street, London WC1E 7HT. Tel: +44 20 7636 8636, Fax: +44 20 7436 5389

For peer review only

Funding statement

This work was supported by NIHR grant number 15/80/28

Competing interests statement

Dr Wing and Dr Williamson declare no competing interests.

Professor Carpenter is funded 60% by a grant from the MRC, via a secondment to the MRC Clinical Trials Unit. The remaining funding is a combination of HEFCE and small contributions from various NIHR, ESRC, MRC and EU funds. He undertakes methodological consultancy work for Novartis and GlaxoSmithKline, and has given missing data courses for GlaxoSmithKline, Bayer and Boehringer. Professor Smeeth reports grants from Wellcome, MRC, NIHR, BHF, Diabetes UK, ESRC and the EU; grants and personal fees for advisory work from GSK, and personal fees for advisory work from AstraZeneca. He is a Trustee of the British Heart Foundation.

Dr Wise is an independent consultant to the pharmaceutical industry and is employed to provide advice by a number of different companies none of which is involved in this therapeutic area.

Professor Schneeweiss is a consultant to WHISCON, LLC and to Aetion, Inc., a software manufacturer of which he also owns equity. He is principal investigator of investigator-initiated grants to the Brigham and Women's Hospital from Bayer, Genentech and Boehringer Ingelheim unrelated to the topic of this study. He does not receive personal fees from biopharmaceutical companies.

Professor Smeeth reports grants from Wellcome, MRC, NIHR, BHF, Diabetes UK, ESRC and the EU; grants and personal fees for advisory work from GSK, and personal fees for advisory work from AstraZeneca. He is a Trustee of the British Heart Foundation.

Dr Quint's research group has received funding from MRC, Wellcome, BLF, GSK, BI, AZ and Insmed for other projects, none of which relate to this work.

Dr Douglas is funded by an unrestricted grant from, has consulted for and holds stock in GlaxoSmithKline.

Word count (not including abstract, tables, figures, or footnotes): 4386

Statement about prior postings and presentations: This work has not been published elsewhere.

For peer review only

Abstract

Introduction

Chronic obstructive pulmonary disease (COPD) is a progressive disease affecting 3 million people in the UK, in which patients exhibit airflow obstruction that is not fully reversible. COPD treatment guidelines are largely informed by randomised controlled trial (RCT) results, but it is unclear if these findings apply to large patient populations not studied in trials. Observational studies could be used to study patient groups excluded from trials, but the use of these studies to estimate treatment effectiveness is in its infancy. In this study we will use individual trial data to validate observational methods for assessing COPD treatment effectiveness, before applying these methods to analysing treatment effectiveness within people excluded from, or under-represented in COPD trials.

Methods and analysis

Using individual patient data from the landmark COPD TORCH trial, we will assemble a cohort in the UK Clinical Practice Research Datalink with similar characteristics to TORCH participants and test whether observational data can generate comparable results to trials, using cohort methodology with propensity score techniques to adjust for potential confounding. We will then use the methodological template we have developed to determine risks and benefits of COPD treatments in people excluded from TORCH. Outcomes are pneumonia, COPD exacerbation, mortality and time to treatment change. Groups to be studied include the elderly (>80 years), people with substantial comorbidity, people with and without underlying cardiovascular disease and people with mild COPD.

Ethics and dissemination

Ethical approval has been granted by the LSHTM Medicines Ethics Committee (Ref: 11997). The study is under review by the Independent Scientific Advisory Committee (ISAC) of the UK Medicines and Healthcare Products Regulatory Agency (MHRA). An application to use the TORCH trial data made to clinicalstudydatarequest.com has been approved. In addition to scientific publications, dissemination methods will be developed based on discussions with COPD patient groups.

For peer review only

Strengths and limitations of this study

Strengths

- Large cohort study
- Use of validated methods for detecting COPD within the Clinical Practice Research Datatlink
- Use of randomised controlled trial (RCT) individual patient data to assess ability of observational methods to detect COPD treatment effects within an RCT-analagous population

Limitations

- Adherence to medication will need to be assessed based on proxy variables (e.g. time covered by prescription)

For peer review only

Introduction

Background and rationale

Chronic obstructive pulmonary disease (COPD) affects 3 million people in the UK.[1] The most common cause is smoking, and patients exhibit airflow obstruction that is not fully reversible. The disease is progressive, with declining lung function and a worsening of symptoms. Most troublesome are acute exacerbations manifested as a sudden worsening of symptoms e.g. severe coughing, shortness of breath and chest congestion, requiring urgent treatment, and possibly hospitalisation. Whilst smoking cessation remains the most effective intervention, the rate of exacerbation can be reduced by regular medication such as combination long-acting beta-agonists (LABA) and inhaled corticosteroids (ICS) or long-acting muscarinic antagonists (LAMAs).[2,3]

COPD treatment guidelines are largely informed by randomised controlled trial (RCT) results,[4] but it is not clear if these findings apply to large patient populations not studied in trials. Fluticasone propionate + salmeterol (FP/SAL) is a LABA/ICS combination and is one of the most widely used COPD treatments. It was studied in large randomised trials (e.g. the TORCH trial),[2] but the effects of treatment in important patient groups who were not studied are unknown. Some were excluded from trials (e.g. those >80 years and those with substantial comorbidity) while others are under represented (e.g. people with mild COPD),[2,5] meaning conclusions about these groups are difficult to make.

Whilst the conduct of observational studies to investigate possible drug harms is well established, the use of these studies to estimate treatment effectiveness is in its infancy. Issues of treatment channelling and indication bias mean that measuring the intended benefit of a treatment is beset with difficulties. Over the next few years we will see more observational studies of drug effectiveness emerging due to recent legislation requiring pharmaceutical companies to study the real world effects of medications;[6,7] however, rigorous, validated methodology is needed to translate these complex data into reliable evidence.

In this study we will use TORCH[2] individual trial data to validate observational methods for assessing COPD treatment effectiveness, before going on to apply these methods to analysing treatment effectiveness within people excluded from or underrepresented in the TORCH trial. Observational data will be obtained from very large UK population-level databases of electronic health records. The results we generate will aid patients, prescribers and policy makers in deciding the most appropriate treatment for COPD for all types of patients. The approach used can also provide a template for treatment effectiveness research using observational data with inbuilt validation against a randomised trial.

For peer review only

Aims and objectives

The aims of our study are (1) to measure the association between treatments for COPD and COPD exacerbation rate, mortality, pneumonia, and time to treatment change amongst patients not included in randomised clinical trials for COPD treatments and (2) to develop a methodological framework with in built validation against RCT data, for using observational electronic health records (EHR) to answer questions about drug treatment effects (i.e. both benefits and risks).

Specific objectives are to: (1) validate methods for measuring COPD medication effectiveness in EHR data by comparing with trial results; (2) use EHR data to measure COPD medication effectiveness in patients excluded from trials (most importantly those >80 years or with substantial comorbidity) and (3) determine COPD treatment effectiveness in an under-studied disease stage (mild COPD).

Methods and analysis

Study design

We have chosen a cohort study design as it will allow us to measure the effects of prescribing different treatments for COPD on future outcomes in different types of patients. Eligibility criteria for cohort entry will vary between objectives (detailed in the selection of participants section below).

Setting/data sources

Patient data used in this study will be obtained from two different sources: the TORCH randomised trial and the UK Clinical Practices Research Datalink (CPRD) (linked to Hospital Episodes Statistics – HES – data).

TORCH

TORCH was a placebo controlled randomised trial of the combined inhaler fluticasone propionate (FP) + salmeterol (SAL) (FP/SAL) for the treatment of COPD, published in 2007. Patients were randomised to receive FP/SAL, FP alone, SAL alone or placebo and the primary comparison of interest was between FP/SAL and placebo.[2] Key outcomes were expected benefits (rate of COPD exacerbation and mortality) and an expected harm due to the immunosuppressive action of the corticosteroid FP (pneumonia). Whilst findings for the primary endpoint of mortality were null, this was thought to be due to poor statistical power as a result of a lower than anticipated mortality rate. Nonetheless, a lower rate of exacerbations was seen with FP/SAL, and a higher rate of pneumonia was observed. As one of the largest trials in COPD, and with three year follow up, TORCH is a landmark study, providing a validation point for our study. We will obtain individual patient data

For peer review only

from the TORCH study via www.clinicalstudydatarequest.com for use in Objective 1 (see selection of participants section below)

CPRD

The CPRD is a very large database of prospectively collected, anonymised UK population-based electronic health records. CPRD primary care records comprise ~8-10% of the UK population and contain comprehensive information on clinical diagnoses, prescribing, referrals, tests and demographic/lifestyle factors.[6] In order to contribute to the database, general practices and other health centres must meet prespecified standards for research-quality data (i.e. be “up to standard”). Data quality/validity are therefore high and the data are nationally representative.[6,7] Linkage between the primary care records in CPRD and hospital episode statistics (HES) is well established for >60% of practices in the CPRD, providing a data set augmented with detailed secondary care diagnostic and procedural records. Validated algorithms have been established to identify COPD, COPD exacerbations and pneumonia (both hospital and primary care managed) in CPRD/HES linked data.[9–11]

Selection of participants

Participants will be selected from the CPRD. All patients will need to have been registered with an up-to standard practice for at least 12 months. Participant selection criteria will then vary by objective as detailed below.

Objective 1: Validation of observational methods by comparing with trial results

An overview of each of the steps for participant selection for Objective 1 is provided in Figure 1.

Step 1

We will select all patients in the CPRD with COPD who are eligible for HES-linkage and during the period covered by the linkage would have met the following eligibility criteria for inclusion in the TORCH study:

- a diagnosis of COPD
- age 40-80 years
- lung function (FEV1<60% predicted, FEV1/FVC ratio <70%)
- smoking history
- no history of asthma
- no history of lung surgery
- no requirement for long-term oxygen therapy
- no diagnosed alpha-1 antitrypsin deficiency
- no evidence of drug/alcohol abuse
- no exposure to FP/SAL within the previous 4 weeks

Feasibility counts in the CPRD indicate there are ~13,000 patients meeting these criteria (Figure 1).

Step 2

Next we will determine if/when these patients ever received FP/SAL. During any time between attaining TORCH eligibility and a subsequent prescription for FP/SAL, patients will be eligible for inclusion as an unexposed patient in Objective 1. There may be multiple time periods within a person's record where eligibility as an unexposed patient is met (Figure 2). Feasibility work shows there are ~11,000 TORCH eligible patients in CPRD who did not receive FP/SAL at the time they attained TORCH eligibility and therefore have at least one time period that means they are eligible for inclusion as an Objective 1 unexposed participant (Figure 1). Individuals in CPRD who have more than one unexposed eligibility period within their record (Figure 2) will be able to contribute more than once to the pool of unexposed participants (with the covariates and person-time contributed unique to the specific unexposed eligibility period).

Step 3

Having obtained individual level patient data for TORCH participants from clinicalstudydatarequest.com, we will then match each TORCH participant (n=6,112) 1:1 with the closest available unexposed patient record in the CPRD pool of FPSAL untreated patients obtained in Step 2. Matching will be based on the following TORCH baseline characteristics:

- age
- sex
- body mass index
- previous treatment with:
    - inhaled corticosteroids
    - long acting beta-agonists
- history of COPD exacerbations
- history of cardiovascular disease
- lung function

Where an individual from CPRD has multiple unexposed "eligibility periods" that can be matched to a TORCH participant, the CPRD characteristics that will be matched on will be those from the beginning of the specific eligibility period.

Some of the TORCH inclusion criteria will not be fully assessable using CPRD data (e.g. we will be able to assess whether patients are smokers but will not always know their pack year history). Hence the inclusion/exclusion criteria are analogous with TORCH criteria but we acknowledge they are not identical. Identification of criteria will be done based on algorithms already determined and by the identification of clinical codes in the CPRD. For those individuals that have contributed multiple unexposed records to the pool of CPRD unexposed participants (Figure 2), after one of their unexposed records has been matched to a TORCH participant we will remove all of their other unexposed records (meaning that an individual can only appear once in the final TORCH-matched unexposed cohort). We anticipate matching all or the majority of TORCH participants with a CPRD

For peer review only

patient, giving us a pool of TORCH-analogous untreated patients within CPRD, with similar baseline characteristics as TORCH participants at the point of randomisation (n~6,000, Figure 1).

Step 4

Following this, we will select all patients in CPRD meeting the TORCH eligibility criteria specified above, and who also received treatment with FP/SAL (either on the date of eligibility or at a later date). From feasibility work, we anticipate ~12,000 eligible FP/SAL treated patients (Figure 1), some of whom may have multiple time-periods of treated patient eligibility and therefore could contribute more than once to the initial pool of treated patients (Figure 2). In contrast to the unexposed TORCH-eligible cohort, our initial approach will not involve matching participants of the exposed TORCH-eligible cohort with participants from the TORCH trial, as this would negatively impact the ability to calculate propensity scores for receiving FP/SAL in Step 5. Note that there will be overlap between those selected as untreated (in Step 2) and as treated (in Step 4). Approximately 90% of the Step 4 treated patients will also have been eligible as Step 2 untreated patients, as they will have had periods where they were not treated with FP/SAL and met the Step 2 untreated eligibility criteria in addition to separate periods where they were treated with FP/SAL and met the Step 4 treated eligibility criteria (Figure 2). If a person is included in both the untreated Step 2 and treated Step 4 cohorts, they will be contributing different periods of their person-time to each cohort (pre-FP/SAL treatment for Step 2 vs post-FP/SAL treatment for Step 4), and this will be handled in the analysis by assigning different index dates for Step 2 compared to Step 4.

Step 5

We will combine the CPRD groups obtained in Steps 3 and 4 (n~18,000, see note in Step 4 relating to 90% overlap) and using their baseline characteristics will calculate propensity scores for receiving FP/SAL. The propensity score calculation will be based upon a wide range of covariates (see statistical analysis section for full details). Where a participant is contributing more than one treated time period record to the pool of exposed records (as described in Step 4), baseline characteristics will be updated at the beginning of each treatment-eligible period. Multiple eligible treatment periods from a single person are then included in the propensity score model as if they came from different individuals. The variables selected for the score will then become the basis for propensity score modelling in Objectives 2 and 3.

Step 6

Each untreated patient derived in Step 3 (n~6,000) will be matched 1:1 with the FP/SAL treated patient record from Step 4 with the closest propensity score (~12,000) giving us an analysis population for Objective 1 of ~12,000 patients – double the size of TORCH (Figure 1). For those individuals that have contributed multiple exposed records to the pool of CPRD exposed participants (Figure 2), after one of their exposed records has been propensity-score matched to an unexposed participant, we will remove all other exposed records for that individual from the remaining pool of

For peer review only

CPRD exposed participants. This will mean that an individual can only appear once as an exposed participant in the final propensity-score matched cohort. The index date will be the start of follow up.

We will also apply an alternative additional approach for Objective 1, where instead of generating and using propensity scores to obtain a final analysis population at Steps 5 and 6, we will match records from our exposed TORCH-eligible cohort with participants from the TORCH trial to create a TORCH-analogous exposed patient cohort. This will then be combined with the TORCH-analogous unexposed patient cohort to create a final analysis population (with multivariable regression techniques used to account for confounding instead of propensity scores).

Objective 2: Measurement of COPD treatment effects in patients excluded from trials

We will include patients with a valid COPD diagnosis in the CPRD[8] meeting these additional criteria: (1) age >80 years OR (2) history of lung surgery OR (3) history of long term oxygen therapy OR (4) evidence of drug/alcohol abuse OR (5) substantial comorbidity. In relation to substantial comorbidity, TORCH required people to be excluded from the trial if they had serious uncontrolled disease with a likelihood of causing death within 3 years. It is likely this criterion affected participant selection and led to a lower overall rate of death than originally anticipated, although we recognise this criterion is subjective. During Objective 1 we will be able to select groups of people who were generally not included despite being eligible, most likely because of this subjective exclusion criterion. We anticipate this will be people with substantial comorbidity e.g. serious vascular disease. Status for such diseases is readily identified in both the CPRD data and in the TORCH baseline data. We will only be able to specify this criterion in detail after we have completed Objective 1.

Objective 3: Determination of treatment effects in an under-studied disease stage

We will include patients with a valid COPD diagnosis in the CPRD[8] meeting these additional criteria: (1) >60% predicted FEV1 (or >50% plus MRC breathlessness scale 1 or 2, or >50% plus COPD Assessment Test (CAT) score <10) AND a maximum of 1 exacerbation in the year post COPD diagnosis AND no evidence of asthma.

For peer review only

Figure 1: Flowchart illustrating the planned selection of CPRD participants for Objective 1 of the COPD real-world medicines effects study

[FIGURE 1 HERE]

For peer review only

Note in relation to Step 5 (n~18,000) compared to Step 1 (n~13,000): Approximately 90% of the treated patients will also have been eligible as untreated patients, as they did not receive FP/SAL on their TORCH-eligibility date. This means that they will have at least one period of time during which they are untreated-eligible) but then did subsequently go-on to receive FP/SAL (meaning they have at least one period of time during which they are treated-eligible). If a person is included as both a treated and untreated participant, they will be contributing different periods of their person-time to each cohort (pre-FP/SAL treatment for the untreated versus post-FP/SAL treatment for treated), and this is handled in the analysis by assigning different index dates.

Figure 2: Example timeline for a person in CPRD who is eligible for participation in Objective 1, illustrating multiple periods of unexposed/exposed eligibility.

[FIGURE 2 HERE]

For peer review only

1. 1st date of study eligibility: person meets TORCH eligibility as detailed in Objective 1, Step 1 on this date i.e. has a diagnosis of COPD, is between 0-80 years, FEV1<60% predicted and FEV1/FVC ratio <70%, smoking history, no asthma history, no lung surgery history, no long-term O_2 therapy, no alpha-1 antitrypsin deficiency, no drug/alcohol abuse, no exposure to FP/SAL within the previous 4 weeks.

2. Eligible to enter study as an untreated participant: patient can be selected as an untreated participant on any date within this period (as detailed in Step 2).

3. Eligible to enter study as a treated participant: FP/SAL treatment starts, patient is able to be selected as a treated study participant on the date that FP/SAL treatment starts (as detailed in Step 4).

4. Eligible to enter study as an untreated pariticipant: patient stops treatment, but is not immediately eligible for selection again as an untreated study participant. After 4 weeks of no FP/SAL treatment however, they meet the TORCH eligibility ciriteria, and may be selected at any date during the remaining one untreated week as an untreated patient. This is the second untreated period that this person can contribute to the total pool of untreated period records that will be available for matching to the TORCH participants (as detailed in Steps 2 and 3).

5. Eligible to enter study as a treated participant: FP/SAL treatment (re)starts, patient can be selected as a treated study participant on the date that FP/SAL treatment (re)starts. This is the second treated period that this person can contribute to the total pool of treated period records that will be available for propensity score matching to the untreated participants (as detailed in Steps 4 to 6).

For peer review only

Exposures, outcomes and co-variates

Exposures

For all objectives, exposures will be determined using CPRD prescribing records and code lists for COPD treatments (codelists provided in supplementary materials).

For Objective 1, incident use of FP/SAL (tradename Seretide) is the primary exposure of interest and will be compared with no treatment. We will limit included patients to those receiving Seretide 500/50, the dose used in TORCH. This information is recorded for all prescriptions of Seretide and this dose is the only currently approved dose for COPD in the UK (though we recognise some prescribing may not follow the licensed indication).

For Objectives 2 and 3 we will again use recorded prescribing information to determine the dose received. We will be reliant mostly on the strength of each individual drug which is recorded automatically against each product and does not require GPs to enter this data, ensuring completeness. We will then be able to stratify analyses based on the dose prescribed.

As a secondary analysis in Objective 1 other treatments for COPD will also be compared with no treatment in this objective. Namely:

a) long-acting beta agonist (LABA)
b) long-acting muscarinic antagonist (LAMA)
c) LABA + LAMA
d) LABA + inhaled corticosteroid (ICS) other than FP/SAL
f) LABA + LAMA + ICS

For Objectives 2 and 3, exposures are as follows:

a) No treatment
b) LABA
c) LAMA
d) LABA + LAMA
e) LABA + ICS
f) LABA + LAMA + ICS

Outcomes

Outcomes to be measured are as follows:

1. COPD exacerbation*:* to be defined using a CPRD-Hospital Episodes Statistics (HES) algorithm developed previously by authors of this study protocol[10]
2. All cause mortality: as recorded in ONS mortality statistics (data that is linked to CPRD data)
3. Pneumonia: as defined using a CPRD-HES algorithm published previously by authors of this study protocol[9]
4. Time to COPD treatment change: determined by prescribing records indicating the start of a new, additional COPD treatment

For peer review only

Covariates

Covariates to be considered for inclusion in the propensity score include the following (all obtained from CPRD data):

- Lung function (FEV1, FEV1/FVC)
- Age
- Gender
- Body mass index
- Alcohol consumption
- Vascular disease (broken into individual components e.g. hypertension, heart failure, atherosclerotic disease)
- Use of prescribed aspirin and statins
- Prior treatment with other COPD medication
- Type 2 diabetes
- History of cancer
- Renal disease
- Healthcare utilisation intensity (number of prior visits, hospitalisations, number of distinct medications used, number of procedures)

Sample size

Assuming a baseline conservative exacerbation rate of 0.5 per patient per year,[10] we would only require a sample of 408 patients per treatment group to detect a reduction in annual exacerbation rate to 0.4 per year, with 80% power and 5% significance. The estimated sample size is ~12,000 for each objective which will provide ample power for the main outcomes of interest, but also allow stratification by patient characteristics to determine stratified results, and will also be ample for the secondary analyses where we will use 99% confidence intervals. For example, to detect a reduction from 0.5 to 0.4 exacerbations per year with 80% power and 1% significance we would need ~600 people in each treatment group. We are also confident that we will have sufficient numbers to allow well powered analyses for Objectives 2 and 3. For example a feasibility count looking at the number of people over the age of 80 eligible for inclusion in Objective 2 estimated that there would be >2,000 people in each exposure group.

Statistical analysis

Propensity score for addressing confounding

The propensity score will be constructed using the principle that predictors of the exposure and outcome, or outcome only (mortality) should be included. We will consider a wide range of factors for inclusion, such as: age, sex, body mass index, alcohol consumption, and a wide range of comorbidities (e.g. type 2 diabetes, coronary heart disease, cerebrovascular disease, peripheral vascular disease, heart failure, hypertension, renal disease, cancer). We will further adjust for healthcare utilisation intensity (number of prior visits, hospitalisations, number of distinct

For peer review only

medications used, number of procedures, etc.) as these are generic correlates of disease state and the likelihood of recording completeness. We have substantial prior experience of building propensity models, and our study team includes the lead of an MRC funded project to determine optimal propensity score methods for use with missing data (EW).

For Objective 1, every untreated patient will be PS matched 1:1 with an FP/SAL treated patient that has an FP/SAL treated period wih the closest propensity score, resulting in a TORCH analogous cohort of FP/SAL treated and untreated CPRD patients (n~12,000). For the additional alternative approach to Objective 1 relying on matching of both unexposed and exposed patients to the TORCH trial patients (described in Selection of participants – Objective 1 – Step 6), we will use multivariable regression techniques to address confounding, considering a similar wide range of covariates for adjustment.

The propensity score model obtained in Objective 1 will be the basis for propensity score modelling in Objectives 2 and 3, but additional variables will also be considered given the different nature of the patient populations being studied in these Objectives.

Methods of analysis

Comparisons will be made according to FP/SAL status for rate of COPD exacerbation, pneumonia and mortality over 3 years. For exacerbations, a negative binomial model will be used, accounting for variability between patients in the number and frequency of exacerbations, with the number of exacerbations as the outcome and the log of treated time as an offset variable. Time to mortality and first pneumonia will be analysed using Cox proportional hazards regression. This mirrors TORCH endpoints of major benefit and harm. We anticipate the propensity matching process will allow us to assemble treated and untreated groups that are very similar with respect to baseline characteristics except FP/SAL treatment status. However, this will be tested by assessing standardised differences for each baseline variable. If substantial differences are noted for important variables, it may be necessary to further adjust the statistical models.[11]

Validation of results against TORCH

We will validate our findings against TORCH by determining whether results of the CPRD analysis are compatible with the TORCH rate ratio for exacerbations (0.75; 95% CI 0.69-0.81). This outcome has been selected as it is an outcome of key significance for people with COPD[4] and the result in TORCH shows a clear benefit with 95% confidence intervals below 1. We have set two criteria that must be met for us to conclude results are consistent. First the effect size must be clinically comparable with TORCH findings; the rate ratio for exacerbations in CPRD must be between 0.65 and 0.9. This range is deliberately not symmetrical around the TORCH estimate of 0.75 as we would anticipate the treatment effect in routine clinical care may be weaker than that seen in the optimised setting of a

randomised trial. We recognise this rule could be met with a poorly powered, inconclusive result, so a second criterion is that the 95% confidence interval for the rate ratio must exclude 1.

Handling measurement of adherence to medication

Adherence to issued prescribing in general practice is likely to vary according to the treatment issued e.g. short course antibiotic treatment is notoriously not well adhered to, whereas long term life-saving treatment such as antiretroviral medication is more likely to be taken as prescribed. Whilst we do not have figures for adherence for COPD medication in UK general practice, we are able to estimate the proportion of time covered by prescribing as a proxy for adherence and will account for this in our analyses. Moreover, our intention is to estimate the effect of prescribing at the population level, and to some extent, the clinical effects we will measure are in part due to pharmacological effects, and in part the way the treatment is taken which includes adherence. Also of note, prescribing for COPD in the UK is predominantly through GPs and so we will not be missing prescribing information from other potential sources of treatment.

The data analysis for adherence will necessarily be a significant element of the work to be done for this study. However, we have reviewed the records for a random sample of 30 people with COPD starting treatment with FP/SAL to look at adherence patterns over the course of a year. Of the 30 patients, 20 (67%) were still receiving Seretide (FP/SAL) one year after starting treatment. Of the 20 who received Seretide for a full year, 15 (75%) received sufficient prescriptions to suggest at least 50% adherence over the year, and 8 (40%) had sufficient prescriptions to suggest 80% adherence or higher. As expected, this suggests two things: Firstly adherence is likely to be poorer in routine clinical care than in the trial population; in TORCH 80% of participants were estimated to have adherence at 80% or higher. Secondly there is a wide range of adherence in routine care. This will allow us to estimate both the population level effects of treatment as actually used in routine care, but also to estimate the treatment effect in patients with more similar levels of adherence to TORCH participants. Whilst we acknowledge that prescribing can only be a proxy for used medication, we believe it is not an unreasonable assumption that the amount of medication prescribed is correlated with the amount consumed. In the event that Objective 1 detects a null or poorer treatment effect than anticipated (rate ratio > 0.9), we will conduct a sensitivity analysis restricted to people estimated to be covered by FP/SAL treatment for 80% of their follow up.

Missing data

CPRD data are shown to be almost complete for drug prescribing and mortality (partly through ONS linkage). Smoking history tends to be very well recorded for people with COPD and missingness is likely to be minimal.[10] Information on important comorbidity is also well recorded in CPRD. We will conduct both complete record analyses and use multiple imputation where appropriate assumptions

For peer review only

hold, applying findings from methodological work led by one of the study team (EW) into the use of multiple imputation in propensity score modelling.[14]

Ethics and dissemination

Approval by ethics and scientific comittees

Ethical approval for this study has been obtained from the London School of Hygiene & Tropical Medicine Ethics Committee (Ref: 11997).

An application for scientific approval related to use of the CPRD data has been made to the Independent Scientific Advisory Committee (ISAC) of the Medicines and Healthcare Products Regulatory Agency. CPRD data are already approved via a National Research Ethics Committee for purely observational research of this type.

An application for use of the TORCH trial individual patient data was made to the clinicalstudydatarequest.com site, which is checked by the Wellcome Trust and relevant sponsors to make sure information is complete and that the sponsor's requirements for informed consent have been met. The application is then sent to an independent review panel that consider the scientific rationale, objectives, publication plan, conflicts of interest and qualifications and experience of the research team before making a decision on providing access to the data. We recently obtained approval of all aspects of this application.

Dissemination plans

Dissemination of findings will be via a combination of channels. The work will be published in high ranking peer reviewed journals and we anticipate 3 publications to arise directly from the planned work. Findings will also be presented at relevant scientific conferences such as the British Thoracic Society Conference and the European Respiratory Society International Congress. We will also engage with patients already identified from a clinic run by one of the authors of this protocol (JQ) and from Breathe Easy Groups and with relevant charities such as the British Lung Foundation to determine the most relevant ways to disseminate results directly to patients in an accessible manner, and to help our understanding of the likely impact of results to specific groups of patients. We will communicate directly with NICE to ensure they are kept informed of results that are of direct relevance to the guidance they have issued on COPD, and with the Medicines and Healthcare Products Regulatory Agency if it appears that findings may impact the risk/benefit profile of COPD treatments.

For peer review only

References

1. Comission H. Cleaning the air: a national study of chronic pulmonary disease. 2006.

2. Calverley PMA, Anderson JA, Celli B, Ferguson GT, Jenkins C, Jones PW, et al. Salmeterol and Fluticasone Propionate and Survival in Chronic Obstructive Pulmonary Disease. N Engl J Med. 2007 Feb 22;356(8):775–89.

3. Tashkin DP, Celli B, Senn S, Burkhart D, Kesten S, Menjoge S, et al. A 4-Year Trial of Tiotropium in Chronic Obstructive Pulmonary Disease. N Engl J Med. 2008 Oct 9;359(15):1543–54.

4. National Institute for Health and Care Excellence (NICE). Chronic obstructive pulmonary disease in over 16s: diagnosis and management | Guidance and guidelines | NICE. 2010;

5. Ferguson GT, Anzueto A, Fei R, Emmett A, Knobil K, Kalberg C, et al. Maintenance pharmacotherapy of mild and moderate COPD: what is the evidence? Respir Med. 2011 Sep;105(9):1268–74.

6. EU. REGULATION (EU) No 1235/2010 OF THE EUROPEAN PARLIAMENT AND OF THE COUNCIL. 2010.

7. Spring S. Guidance for Industry Postmarketing Studies and Clinical Trials — Implementation of Section 505(o)(3) of the Federal Food, Drug, and Cosmetic Act.

8. Herrett E, Gallagher AM, Bhaskaran K, Forbes H, Mathur R, Staa T van, et al. Data Resource Profile: Clinical Practice Research Datalink (CPRD). Int J Epidemiol. 2015 Jun;44(3):827–36.

9. Herrett E, Thomas SL, Schoonen WM, Smeeth L, Hall AJ. Validation and validity of diagnoses in the General Practice Research Database: a systematic review. Br J Clin Pharmacol. 2010 Jan;69(1):4–14.

10. Quint JK, Müllerova H, DiSantostefano RL, Forbes H, Eaton S, Hurst JR, et al. Validation of chronic obstructive pulmonary disease recording in the Clinical Practice Research Datalink (CPRD-GOLD). BMJ Open. 2014;4(7):e005540.

11. Millett ERC, Quint JK, Smeeth L, Daniel RM, Thomas SL. Incidence of Community-Acquired Lower Respiratory Tract Infections and Pneumonia among Older Adults in the United Kingdom: A Population-Based Study. Heimesaat MM, editor. PLoS One. 2013 Sep 11;8(9):e75131.

12. Rothnie KJ, Müllerová H, Hurst JR, Smeeth L, Davis K, Thomas SL, et al. Validation of the Recording of Acute Exacerbations of COPD in UK Primary Care Electronic Healthcare Records. Chotirmall SH, editor. PLoS One. 2016 Mar 9;11(3):e0151357.

For peer review only

13. Austin PC. An Introduction to Propensity Score Methods for Reducing the Effects of Confounding in Observational Studies. Multivariate Behav Res. 2011 May;46(3):399–424.

14. Leyrat C, Seaman SR, White IR, Douglas I, Smeeth L, Kim J, et al. Propensity score analysis with partially observed covariates: How should multiple imputation be used? Stat Methods Med Res. 2017 Jun 2;96228021771303.

Authors contributions

KW, EW, JC, LW, SS, LS, JQ, ID contributed to study question and design. KW wrote the first draft of the protocol manuscript (based upon original grant/scientific approval applications to NIHR and ISAC that KW, EW, JC, LW, SS, LS, JQ and ID all contributed to). KW, EW, JC, LW, SS, LS, JQ, ID contributed to further drafts and approved the final version.

Acknowledgements

No information for this section.

Data sharing statement

There are currently no unpublished data from this study, as it is a protocol.

All of the data sources described can be accessed by making formal applications to the owners of the data (i.e. CPRD/HES data for the routinely collected observational data and clinicalstudyrequest.com for the trial data used for validation of observational methods).

For peer review only

FIGURE 1

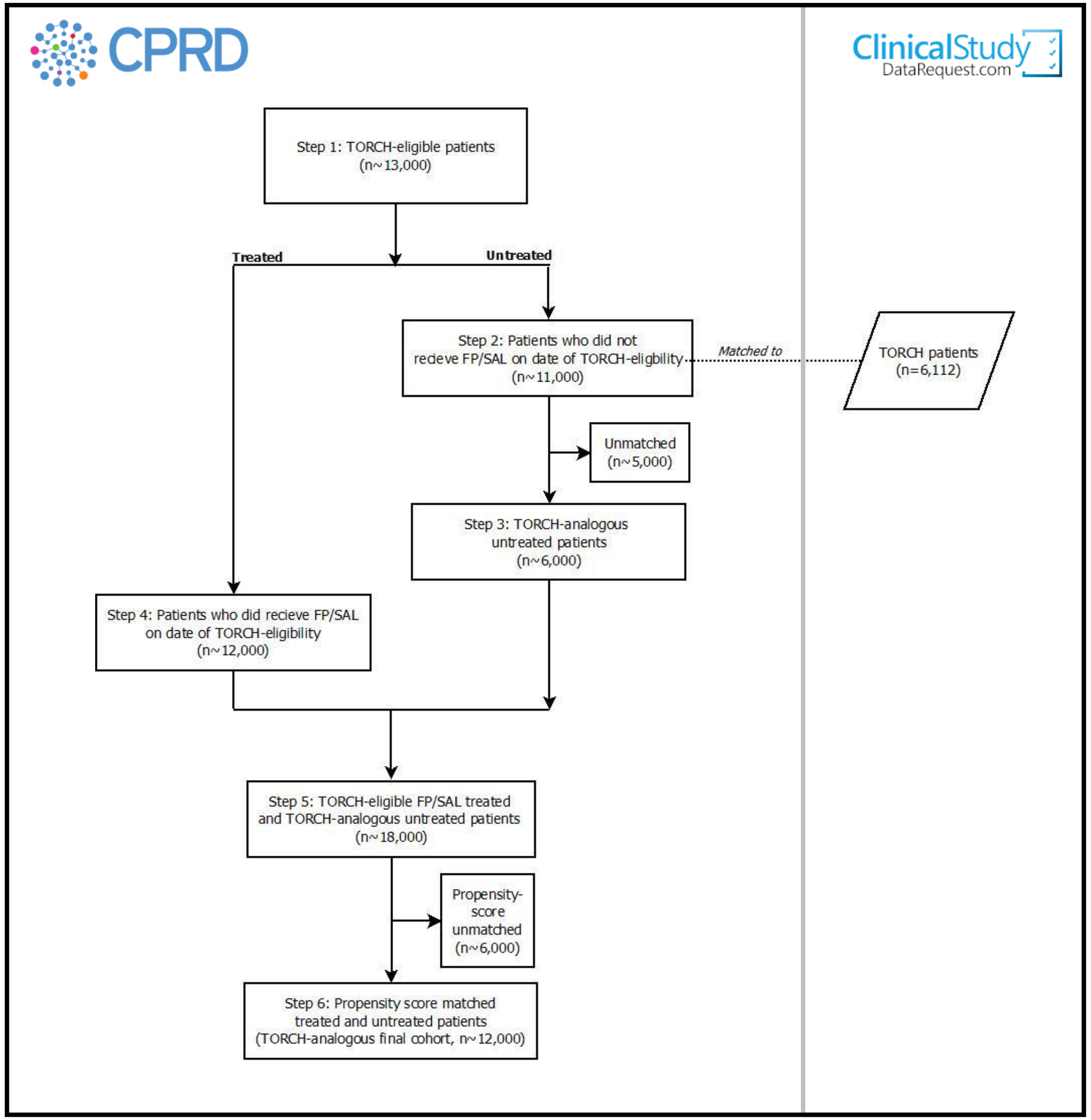
CPRD
ClinicalStudy DataRequest.com
Step 1: TORCH-eligible patients (n~13,000)
Treated
Untreated
Step 2: Patients who did not recieve FP/SAL on date of TORCH-eligbility (n~11,000)
Matched to
TORCH patients (n=6,112)
Unmatched (n~5,000)
Step 3: TORCH-analogous untreated patients (n~6,000)
Step 4: Patients who did recieve FP/SAL on date of TORCH-eligibility (n~12,000)
Step 5: TORCH-eligible FP/SAL treated and TORCH-analogous untreated patients (n~18,000)
Propensity-score unmatched (n~6,000)
Step 6: Propensity score matched treated and untreated patients (TORCH-analogous final cohort, n~12,000)

FIGURE 2

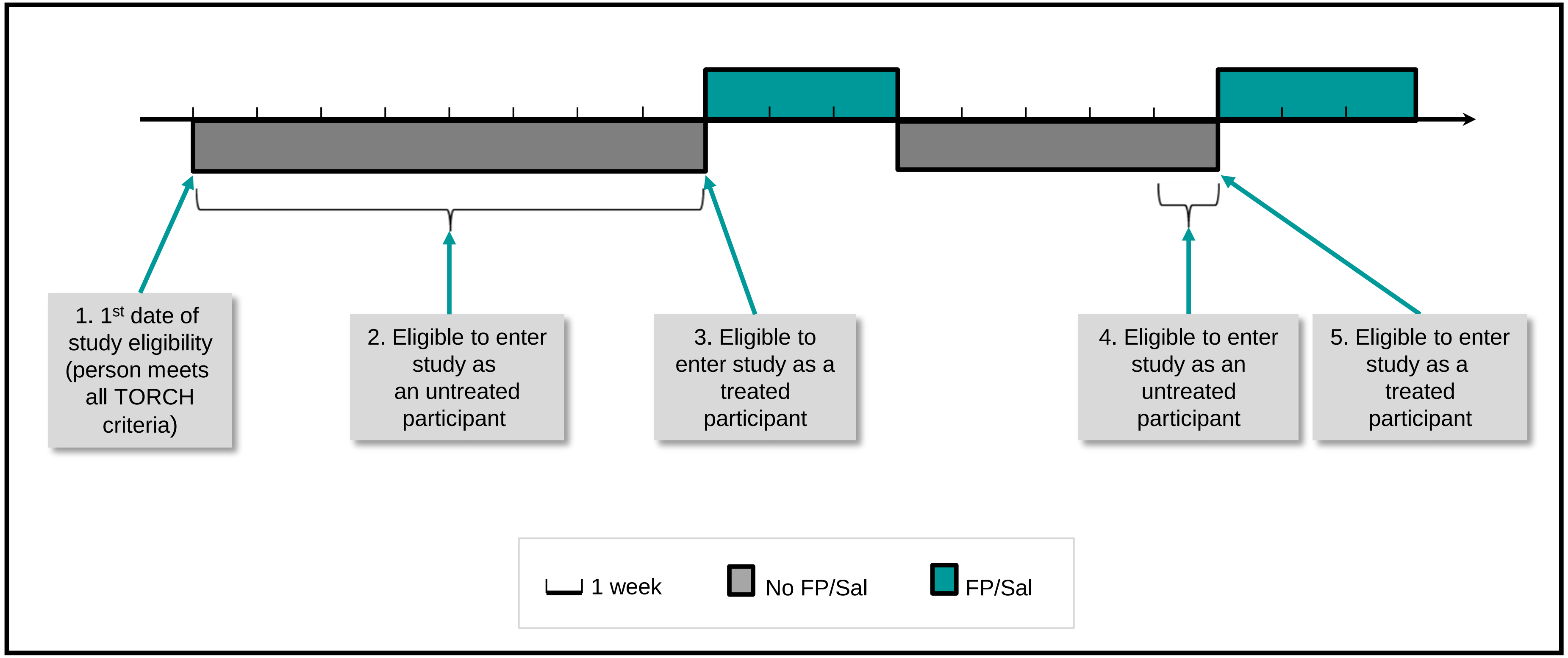
1. 1st date of study eligibility (person meets all TORCH criteria)
2. Eligible to enter study as an untreated participant
3. Eligible to enter study as a treated participant
4. Eligible to enter study as an untreated participant
5. Eligible to enter study as a treated participant
1 week
No FP/Sal
FP/Sal

LAMA codes

Product code	BNF header	Drug substance	Drug product
61176	compound bronchodilator preparations	vilanterol trifenatate/umeclidinium bromide	anoro ellipta 55micrograms/dose / 22micrograms/dose dry powder inhaler (glaxosmithkline uk ltd)
61490	compound bronchodilator preparations		umeclidinium bromide 65micrograms/dose / vilanterol 22micrograms/dose dry powder inhaler
35014	antimuscarinic bronchodilators	tiotropium bromide monohydrate	tiotropium bromide 18microgram inhalation powder capsules with device
6474	antimuscarinics	glycopyrronium bromide	robinul 1mg tablet (idis world medicines)
50577	antimuscarinic bronchodilators	tiotropium bromide	spiriva 18microgram inhalation powder capsules with handihaler (de pharmaceuticals)
35011	antimuscarinic bronchodilators	tiotropium bromide monohydrate	tiotropium bromide 18microgram inhalation powder capsules
49227	antimuscarinic bronchodilators		aclidinium bromide 375micrograms/dose dry powder inhaler
50103	antimuscarinic bronchodilators		spiriva 18microgram inhalation powder capsules with handihaler (waymade healthcare plc)
7908	antimuscarinics	glycopyrronium bromide	robinul 2mg tablet (wyeth pharmaceuticals)
59638	antimuscarinic bronchodilators		spiriva 18microgram inhalation powder capsules with handihaler (sigma pharmaceuticals plc)
51967	antimuscarinic bronchodilators	tiotropium bromide	spiriva 18microgram inhalation powder capsules (mawdsley-brooks & company ltd)
53982	antimuscarinic bronchodilators		seebri breezhaler 44microgram inhalation powder capsules with device (novartis pharmaceuticals uk ltd)
6050	antimuscarinic bronchodilators	tiotropium bromide	spiriva 18 microgram capsule (boehringer ingelheim ltd)
7597	other ulcer healing drugs	glycopyrronium bromide	glycopyrronium bromide 2mg tablets
49228	antimuscarinic bronchodilators		eklira 322micrograms/dose genuair (almirall ltd)
59173	other ulcer healing drugs	glycopyrronium bromide	glycopyrronium bromide 200micrograms/5ml oral suspension
36864	antimuscarinic bronchodilators	tiotropium bromide	tiotropium bromide 2.5micrograms/dose solution for inhalation cartridge with device cfc free
55911	other ulcer healing drugs	glycopyrronium bromide	glycopyrronium bromide 500micrograms/5ml oral solution

For peer review only

Product code	BNF header	Drug substance	Drug product
34995	antimuscarinic bronchodilators	tiotropium bromide	spiriva 18microgram inhalation powder capsules with handihaler (boehringer ingelheim ltd)
35000	antimuscarinic bronchodilators	tiotropium bromide	spiriva 18microgram inhalation powder capsules (boehringer ingelheim ltd)
29138	other ulcer healing drugs	glycopyrronium bromide	glycopyrronium bromide 1mg/5ml oral solution
47269	other ulcer healing drugs	glycopyrronium bromide	glycopyrronium bromide 1mg/5ml oral suspension
54151	other ulcer healing drugs	glycopyrronium bromide	glycopyrronium bromide 600micrograms/5ml oral suspension
55795	other ulcer healing drugs	glycopyrronium bromide	glycopyrronium bromide 500micrograms/5ml oral suspension
38377	other ulcer healing drugs	glycopyrronium bromide	glycopyrronium bromide 2mg/5ml oral solution
7218	other ulcer healing drugs	glycopyrronium bromide	glycopyrronium bromide 1mg tablets
36869	antimuscarinic bronchodilators	tiotropium bromide	spiriva respimat 2.5micrograms/dose solution for inhalation cartridge with device (boehringer ingelheim ltd)
62109	antimuscarinic bronchodilators		umeclidinium bromide 65micrograms/dose dry powder inhaler
50292	antimuscarinic bronchodilators		spiriva 18microgram inhalation powder capsules (sigma pharmaceuticals plc)
55794	other ulcer healing drugs	glycopyrronium bromide	glycopyrronium bromide 5mg/5ml oral suspension
50047	other ulcer healing drugs	glycopyrronium bromide	glycopyrronium bromide 5mg/5ml oral solution
56262	other ulcer healing drugs	glycopyrronium bromide	glycopyrronium bromide 200micrograms/5ml oral solution
61879	antimuscarinic bronchodilators		incruse ellipta 55micrograms/dose dry powder inhaler (glaxosmithkline uk ltd)
53761	antimuscarinic bronchodilators		glycopyrronium bromide 55microgram inhalation powder capsules with device
746	antimuscarinic bronchodilators	tiotropium bromide	tiotropium 18 microgram capsule
38538	other ulcer healing drugs	glycopyrronium bromide	glycopyrronium bromide 2mg/5ml oral suspension
46214	antimuscarinics	glycopyrronium bromide	glycopyrronium bromide 5mg/5ml oral solution
61582	antimuscarinic bronchodilators	tiotropium bromide	spiriva respimat 2.5micrograms/dose solution for inhalation cartridge with device (waymade healthcare plc)

For peer review only

LABA codes

Product code	BNF header	Drug substance	Drug product
45610	selective beta 2 agonists	indacaterol maleate	indacaterol 300microgram inhalation powder capsules with device
7270	selective beta 2 agonists	salmeterol xinafoate	salmeterol 25micrograms/dose inhaler cfc free
10968	selective beta 2 agonists	formoterol fumarate dihydrate	foradil 12microgram inhalation powder capsules with device (novartis pharmaceuticals uk ltd)
549	unknown	salmeterol xinafoate	serevent 25micrograms/dose inhaler (glaxosmithkline uk ltd)
7133	selective beta 2 agonists	formoterol fumarate dihydrate	formoterol 12micrograms/dose dry powder inhaler
54742	selective beta 2 agonists	salmeterol xinafoate	salmeterol 25micrograms/dose inhaler cfc free (a a h pharmaceuticals ltd)
719	selective beta 2 agonists	salmeterol xinafoate	salmeterol 50micrograms/dose dry powder inhaler
57694	selective beta 2 agonists	salmeterol xinafoate	vertine 25micrograms/dose inhaler cfc free (teva uk ltd)
26829	selective beta 2 agonists	tulobuterol	brelomax 2mg tablet (abbott laboratories ltd)
19799	selective beta 2 agonists	tulobuterol	tulobuterol 2mg
56482	selective beta 2 agonists	formoterol fumarate dihydrate	oxis 12 turbohaler (waymade healthcare plc)
25784	selective beta 2 agonists	formoterol fumarate dihydrate	atimos modulite 12micrograms/dose inhaler (chiesi ltd)
47638	selective beta 2 agonists	salmeterol xinafoate	neovent 25micrograms/dose inhaler cfc free (kent pharmaceuticals ltd)
10672	peripheral vasodilators and related drugs	moxisylyte hydrochloride	opilon 40mg tablet (concord pharmaceuticals ltd)
50051	selective beta 2 agonists	salmeterol xinafoate	serevent 25micrograms/dose evohaler (waymade healthcare plc)
35725	selective beta 2 agonists	formoterol fumarate dihydrate	formoterol easyhaler 12micrograms/dose dry powder inhaler (orion pharma (uk) ltd)
2224	selective beta 2 agonists	salmeterol xinafoate	serevent 50micrograms/dose accuhaler (glaxosmithkline uk ltd)
43893	selective beta 2 agonists	indacaterol maleate	onbrez breezhaler 150microgram inhalation powder capsules with device (novartis pharmaceuticals uk ltd)
6526	selective beta 2 agonists	formoterol fumarate dihydrate	formoterol 12microgram inhalation powder capsules with device
43738	selective beta 2 agonists	indacaterol maleate	indacaterol 150microgram inhalation powder capsules with device

For peer review only

Product code	BNF header	Drug substance	Drug product
35825	selective beta 2 agonists	salmeterol xinafoate	serevent 50microgram disks (glaxosmithkline uk ltd)
43764	peripheral vasodilators and related drugs	moxisylyte hydrochloride	opilon 40mg tablets (archimedes pharma uk ltd)
56478	selective beta 2 agonists	salmeterol xinafoate	serevent 50micrograms/dose accuhaler (de pharmaceuticals)
42103	selective beta 2 agonists	tulobuterol	tulobuterol 1mg/5ml sugar free syrup
57544	selective beta 2 agonists	salmeterol xinafoate	serevent 50micrograms/dose accuhaler (waymade healthcare plc)
3297	selective beta 2 agonists	salmeterol xinafoate	salmeterol 50micrograms disc
57558	selective beta 2 agonists	formoterol fumarate dihydrate	oxis 6 turbohaler (lexon (uk) ltd)
9711	selective beta 2 agonists	formoterol fumarate dihydrate	formoterol 6micrograms/dose dry powder inhaler
35542	selective beta 2 agonists	salmeterol xinafoate	salmeterol 50microgram inhalation powder blisters with device
465	unknown	salmeterol xinafoate	salmeterol 25micrograms/dose inhaler
44064	selective beta 2 agonists	indacaterol maleate	onbrez breezhaler 300microgram inhalation powder capsules with device (novartis pharmaceuticals uk ltd)
35165	selective beta 2 agonists	salmeterol xinafoate	serevent 50microgram disks with diskhaler (glaxosmithkline uk ltd)
14306	selective beta 2 agonists	formoterol fumarate dihydrate	formoterol 12micrograms/dose inhaler cfc free
1974	selective beta 2 agonists	formoterol fumarate dihydrate	oxis 12 turbohaler (astrazeneca uk ltd)
7268	selective beta 2 agonists	salmeterol xinafoate	serevent 25micrograms/dose evohaler (glaxosmithkline uk ltd)
8365	peripheral vasodilators and related drugs	moxisylyte hydrochloride	moxisylyte 40mg tablets
35503	selective beta 2 agonists	salmeterol xinafoate	salmeterol 50microgram inhalation powder blisters
1975	selective beta 2 agonists	formoterol fumarate dihydrate	oxis 6 turbohaler (astrazeneca uk ltd)
22663	selective beta 2 agonists	tulobuterol	respacal 2mg tablet (ucb pharma ltd)
910	selective beta 2 agonists	salmeterol xinafoate	serevent diskhaler 50microgram inhalation powder (glaxo wellcome uk ltd)

For peer review only

ICS codes

Product code	BNF header	Drug substance	Drug product
54399	corticosteroids (for respiratory conditions)	beclometasone dipropionate	qvar 100 autohaler (sigma pharmaceuticals plc)
959	unknown	budesonide	budesonide 50micrograms/dose inhaler
2951	corticosteroids (for respiratory conditions)	fluticasone propionate	fluticasone 250microgram/actuation pressurised inhalation
50129	corticosteroids (for respiratory conditions)	beclometasone dipropionate	qvar 100micrograms/dose easi-breathe inhaler (de pharmaceuticals)
2229	corticosteroids (for respiratory conditions)	beclometasone dipropionate	becodisks 100microgram disc (allen & hanburys ltd)
3989	corticosteroids (for respiratory conditions)	fluticasone propionate	flixotide 100microgram disc (allen & hanburys ltd)
5551	corticosteroids (for respiratory conditions)	fluticasone propionate	flixotide 0.5mg/2ml nebules (glaxosmithkline uk ltd)
34794	corticosteroids (for respiratory conditions)	beclometasone dipropionate	beclometasone 200micrograms/dose inhaler (a a h pharmaceuticals ltd)
41269	corticosteroids (for respiratory conditions)	beclometasone dipropionate	beclometasone 400 cyclocaps (teva uk ltd)
29325	corticosteroids (for respiratory conditions)	beclometasone dipropionate	beclometasone 250micrograms/dose inhaler (generics (uk) ltd)
4499	corticosteroids (for respiratory conditions)	beclometasone dipropionate	aerobec 250microgram/actuation pressurised inhalation (meda pharmaceuticals ltd)
57589	corticosteroids (for respiratory conditions)	beclometasone dipropionate	becloforte 250micrograms/dose inhaler (dowelhurst ltd)
32874	corticosteroids (for respiratory conditions)	beclometasone dipropionate	beclometasone 50microgram/actuation inhalation powder (actavis uk ltd)
2159	corticosteroids (for respiratory conditions)	beclometasone dipropionate	aerobec 50 autohaler (meda pharmaceuticals ltd)
14590	corticosteroids (for respiratory conditions)	beclometasone dipropionate	asmabec 250microgram/actuation spacehaler (celltech pharma europe ltd)
1725	corticosteroids (for respiratory conditions)	beclometasone dipropionate	beclazone 50 easi-breathe inhaler (teva uk ltd)
908	corticosteroids (for respiratory conditions)	budesonide	pulmicort 400 turbohaler (astrazeneca uk ltd)
35288	corticosteroids (for respiratory conditions)	beclometasone dipropionate	beclometasone 400microgram inhalation powder blisters
49367	corticosteroids (for respiratory conditions)	beclometasone dipropionate	clenil modulite 50micrograms/dose inhaler (mawdsley-brooks & company ltd)
35107	corticosteroids (for respiratory conditions)	beclometasone dipropionate	beclometasone 400microgram inhalation powder blisters with device
1676	corticosteroids (for respiratory conditions)	fluticasone propionate	flixotide 125microgram/actuation inhalation powder

For peer review only

Product code	BNF header	Drug substance	Drug product
			(allen & hanburys ltd)
49772	corticosteroids (for respiratory conditions)	fluticasone propionate	fluticasone 250micrograms/dose evohaler (sigma pharmaceuticals plc)
48088	unknown	budesonide	budenofalk 9mg gastro-resistant granules sachets (dr. falk pharma uk ltd)
33258	corticosteroids (for respiratory conditions)	beclometasone dipropionate	beclometasone 250micrograms/dose inhaler (a a h pharmaceuticals ltd)
13037	corticosteroids (for respiratory conditions)	beclometasone dipropionate	pulvinal beclometasone dipropionate 200micrograms/dose dry powder inhaler (chiesi ltd)
19031	corticosteroids (for respiratory conditions)	beclometasone dipropionate	bdp 100microgram/actuation spacehaler (celltech pharma europe ltd)
16158	corticosteroids (for respiratory conditions)	beclometasone dipropionate	clenil modulite 50micrograms/dose inhaler (chiesi ltd)
2124	unknown		pulmicort refil 200 mcg inh
4759	corticosteroids (for respiratory conditions)	beclometasone dipropionate	beclometasone 100microgram inhalation powder capsules
3753	unknown		flixotide diskhaler-community pack 250 mcg
49711	corticosteroids (for respiratory conditions)	budesonide	pulmicort 200micrograms/dose inhaler (astrazeneca uk ltd)
15326	corticosteroids (for respiratory conditions)	beclometasone dipropionate	beclometasone 100micrograms/dose inhaler cfc free
2335	corticosteroids (for respiratory conditions)	beclometasone dipropionate	qvar 100 inhaler (teva uk ltd)
5580	corticosteroids (for respiratory conditions)	fluticasone propionate	flixotide accuhaler 50 50microgram/inhalation inhalation powder (allen & hanburys ltd)
51681	corticosteroids (for respiratory conditions)	beclometasone dipropionate	qvar 100 inhaler (sigma pharmaceuticals plc)
8433	corticosteroids (for respiratory conditions)	budesonide	budesonide 100micrograms/actuation inhaler
9577	corticosteroids (for respiratory conditions)	beclometasone dipropionate	asmabec 50 clickhaler (focus pharmaceuticals ltd)
2723	corticosteroids (for respiratory conditions)	fluticasone propionate	fluticasone 25micrograms/dose inhaler
23675	unknown		pulmicort l.s. refil
35638	corticosteroids (for respiratory conditions)	fluticasone propionate	fluticasone propionate 100microgram inhalation powder blisters with device
9571	corticosteroids (for respiratory conditions)	beclometasone dipropionate	beclometasone 250micrograms/actuation vortex inhaler
51234	corticosteroids (for respiratory conditions)	beclometasone dipropionate	qvar 100 inhaler (waymade healthcare plc)
60946	corticosteroids (in chronic bowel disorders)	budesonide	entocort cr 3mg capsules (waymade healthcare plc)

For peer review only

Product code	BNF header	Drug substance	Drug product
5223	corticosteroids (for respiratory conditions)	fluticasone propionate	fluticasone 50micrograms/dose inhaler cfc free
53057	corticosteroids (for respiratory conditions)	fluticasone propionate	flixotide 50micrograms/dose evohaler (lexon (uk) ltd)
13290	corticosteroids (for respiratory conditions)	beclometasone dipropionate	clenil modulite 100micrograms/dose inhaler (chiesi ltd)
42928	corticosteroids (for respiratory conditions)	fluticasone propionate	flixotide 100micrograms/dose accuhaler (glaxosmithkline uk ltd)
14736	corticosteroids (for respiratory conditions)	beclometasone dipropionate	pulvinal beclometasone dipropionate 400micrograms/dose dry powder inhaler (chiesi ltd)
52806	corticosteroids (for respiratory conditions)	beclometasone dipropionate	qvar 100 autohaler (lexon (uk) ltd)
57525	corticosteroids (for respiratory conditions)	fluticasone propionate	flixotide 250micrograms/dose accuhaler (stephar (u.k.) ltd)
33849	corticosteroids (for respiratory conditions)	beclometasone dipropionate	beclometasone 100microgram/actuation inhalation powder (neo laboratories ltd)
52732	corticosteroids (for respiratory conditions)	budesonide	pulmicort 0.5mg respules (necessity supplies ltd)
35611	corticosteroids (for respiratory conditions)	fluticasone propionate	flixotide 250microgram disks (glaxosmithkline uk ltd)
8111	corticosteroids (for respiratory conditions)	beclometasone dipropionate	becloforte vm 250microgram/actuation vm pack (allen & hanburys ltd)
39879	unknown	budesonide	budesonide 200micrograms/dose inhaler cfc free
1242	corticosteroids (for respiratory conditions)	beclometasone dipropionate	beclometasone 250micrograms/dose inhaler
26665	unknown		pulmicort complete
37447	corticosteroids (for respiratory conditions)	fluticasone propionate	fluticasone propionate 50microgram inhalation powder blisters
5718	corticosteroids (for respiratory conditions)	fluticasone propionate	flixotide 125micrograms/dose evohaler (glaxosmithkline uk ltd)
7653	corticosteroids (for respiratory conditions)	beclometasone dipropionate	beclometasone 400microgram inhalation powder capsules
28640	corticosteroids (for respiratory conditions)	beclometasone dipropionate	beclometasone 100microgram/actuation inhalation powder (actavis uk ltd)
4365	corticosteroids (for respiratory conditions)	beclometasone dipropionate	beclometasone 100micrograms disc
34739	corticosteroids (for respiratory conditions)	beclometasone dipropionate	beclometasone 50micrograms/dose inhaler (teva uk ltd)
8635	corticosteroids (for respiratory conditions)	fluticasone propionate	flixotide 50microgram disc (allen & hanburys ltd)
57579	corticosteroids (for respiratory conditions)	fluticasone propionate	flixotide 50micrograms/dose accuhaler (de pharmaceuticals)
4413	corticosteroids (for respiratory conditions)	beclometasone dipropionate	qvar 100 autohaler (teva uk ltd)

For peer review only

Product code	BNF header	Drug substance	Drug product
37203	corticosteroids (in chronic bowel disorders)	beclometasone dipropionate	beclometasone 5mg gastro-resistant modified-release tablets
4132	corticosteroids (for respiratory conditions)	fluticasone propionate	fluticasone 125microgram/actuation pressurised inhalation
3018	corticosteroids (for respiratory conditions)	beclometasone dipropionate	beclometasone 50micrograms/dose inhaler
9233	corticosteroids (for respiratory conditions)	beclometasone dipropionate	beclometasone 200microgram inhalation powder capsules
2160	corticosteroids (for respiratory conditions)	beclometasone dipropionate	beclometasone 50micrograms/dose breath actuated inhaler
2148	corticosteroids (for respiratory conditions)	beclometasone dipropionate	beclometasone 400microgram disc
34919	corticosteroids (for respiratory conditions)	beclometasone dipropionate	beclometasone 50micrograms/dose inhaler (a a h pharmaceuticals ltd)
2092	corticosteroids (for respiratory conditions)	budesonide	budesonide 200micrograms/dose dry powder inhaler
21005	corticosteroids (for respiratory conditions)	beclometasone dipropionate	beclometasone 250micrograms/dose inhaler cfc free
10090	corticosteroids (for respiratory conditions)	beclometasone dipropionate	beclometasone 50micrograms/actuation extrafine particle cfc free inhaler
1551	corticosteroids (for respiratory conditions)	beclometasone dipropionate	beclazone 250 inhaler (teva uk ltd)
39067	corticosteroids (in chronic bowel disorders)	beclometasone dipropionate	clipper 5mg gastro-resistant modified-release tablets (chiesi ltd)
51415	corticosteroids (for respiratory conditions)	beclometasone dipropionate	qvar 50 inhaler (mawdsley-brooks & company ltd)
35106	corticosteroids (for respiratory conditions)	beclometasone dipropionate	becodisks 100microgram with diskhaler (glaxosmithkline uk ltd)
35299	corticosteroids (for respiratory conditions)	beclometasone dipropionate	becodisks 400microgram (glaxosmithkline uk ltd)
54207	corticosteroids (for respiratory conditions)	beclometasone dipropionate	qvar 50 inhaler (de pharmaceuticals)
3927	corticosteroids (for respiratory conditions)	beclometasone dipropionate	filair 100 inhaler (meda pharmaceuticals ltd)
17670	corticosteroids (for respiratory conditions)	budesonide	easyhaler budesonide 100micrograms/dose dry powder inhaler (orion pharma (uk) ltd)
883	corticosteroids (for respiratory conditions)	beclometasone dipropionate	becodisks 200microgram disc (allen & hanburys ltd)
8450	unknown		flixotide diskhaler-community pack 50 mcg
3289	corticosteroids (for respiratory conditions)	fluticasone propionate	flixotide 25micrograms/dose inhaler (glaxosmithkline uk ltd)
53480	corticosteroids (for respiratory conditions)	beclometasone dipropionate	qvar 100 autohaler (stephar (u.k.) ltd)
39102	unknown	budesonide	budesonide 100micrograms/dose inhaler cfc free
20763	unknown		becloforte

For peer review only

Product code	BNF header	Drug substance	Drug product
35374	corticosteroids (for respiratory conditions)	fluticasone propionate	flixotide 500microgram disks (glaxosmithkline uk ltd)
39200	corticosteroids (for respiratory conditions)	beclometasone dipropionate	aerobec forte 250 autohaler (meda pharmaceuticals ltd)
9599	corticosteroids (for respiratory conditions)	beclometasone dipropionate	beclazone 50microgram/actuation inhalation powder (actavis uk ltd)
5804	corticosteroids (for respiratory conditions)	beclometasone dipropionate	beclometasone 250micrograms/dose dry powder inhaler
47943	corticosteroids (for respiratory conditions)	beclometasone dipropionate	beclazone easi-breathe (roi) 100microgram/actuation pressurised inhalation (ivax pharmaceuticals ireland)
5683	corticosteroids (for respiratory conditions)	fluticasone propionate	flixotide 250micrograms/dose evohaler (glaxosmithkline uk ltd)
48340	corticosteroids (for respiratory conditions)	beclometasone dipropionate	clenil modulite 100micrograms/dose inhaler (mawdsley-brooks & company ltd)
1885	corticosteroids (for respiratory conditions)	beclometasone dipropionate	beclazone 200 inhaler (teva uk ltd)
911	corticosteroids (for respiratory conditions)	fluticasone propionate	flixotide accuhaler 250 250microgram/inhalation inhalation powder (allen & hanburys ltd)
6095	corticosteroids (in chronic bowel disorders)	budesonide	budesonide 3mg gastro-resistant capsules
28073	corticosteroids (for respiratory conditions)	beclometasone dipropionate	beclometasone 250microgram/actuation pressurised inhalation (approved prescription services ltd)
4131	corticosteroids (for respiratory conditions)	fluticasone propionate	fluticasone 100microgram disc
43074	corticosteroids (for respiratory conditions)	fluticasone propionate	flixotide 500micrograms/dose accuhaler (glaxosmithkline uk ltd)
35905	corticosteroids (for respiratory conditions)	fluticasone propionate	fluticasone propionate 250microgram inhalation powder blisters
27583	unknown		pulmicort
17654	corticosteroids (for respiratory conditions)	beclometasone dipropionate	easyhaler beclometasone 200micrograms/dose dry powder inhaler (orion pharma (uk) ltd)
4545	corticosteroids (for respiratory conditions)	budesonide	pulmicort ls 50microgram refill canister (astrazeneca uk ltd)
1412	corticosteroids (for respiratory conditions)	fluticasone propionate	flixotide 250microgram/actuation inhalation powder (allen & hanburys ltd)
3363	corticosteroids (for respiratory conditions)	beclometasone dipropionate	becloforte 400microgram disks with diskhaler (glaxosmithkline uk ltd)
36462	corticosteroids (for respiratory conditions)	fluticasone propionate	fluticasone propionate 500microgram inhalation

For peer review only

Product code	BNF header	Drug substance	Drug product
			powder blisters
56471	corticosteroids (for respiratory conditions)	beclometasone dipropionate	becodisks 200microgram (mawdsley-brooks & company ltd)
14294	corticosteroids (for respiratory conditions)	beclometasone dipropionate	qvar 50micrograms/dose easi-breathe inhaler (teva uk ltd)
3570	corticosteroids (for respiratory conditions)	budesonide	budesonide 200micrograms/actuation refill canister
9164	corticosteroids (for respiratory conditions)	fluticasone propionate	fluticasone propionate 50micrograms/dose dry powder inhaler
27188	corticosteroids (for respiratory conditions)	budesonide	easyhaler budesonide 200micrograms/dose dry powder inhaler (orion pharma (uk) ltd)
39099	unknown	budesonide	pulmicort 100micrograms/dose inhaler cfc free (astrazeneca uk ltd)
4926	corticosteroids (for respiratory conditions)	fluticasone propionate	flixotide accuhaler 100 100microgram/inhalation inhalation powder (allen & hanburys ltd)
1642	corticosteroids (for respiratory conditions)	budesonide	budesonide 400micrograms/dose dry powder inhaler
34859	corticosteroids (for respiratory conditions)	beclometasone dipropionate	beclometasone 250microgram/actuation inhalation powder (neo laboratories ltd)
16054	corticosteroids (for respiratory conditions)	budesonide	budesonide 200micrograms/actuation breath actuated powder inhaler
960	corticosteroids (for respiratory conditions)	budesonide	pulmicort 100 turbohaler (astrazeneca uk ltd)
51480	corticosteroids (for respiratory conditions)	beclometasone dipropionate	qvar 100 autohaler (de pharmaceuticals)
7891	corticosteroids (for respiratory conditions)	fluticasone propionate	fluticasone 500microgram disc
56462	corticosteroids (for respiratory conditions)	beclometasone dipropionate	becodisks 400microgram (waymade healthcare plc)
7638	corticosteroids (for respiratory conditions)	fluticasone propionate	fluticasone 250microgram disc
15706	corticosteroids (for respiratory conditions)	beclometasone dipropionate	beclometasone 100 micrograms/actuation vortex inhaler
27915	unknown		fluticasone prop disk refill
2893	corticosteroids (for respiratory conditions)	beclometasone dipropionate	beclometasone 200micrograms disc
5885	corticosteroids (for respiratory conditions)	fluticasone propionate	fluticasone propionate 100micrograms/dose dry powder inhaler
3898	corticosteroids (in chronic bowel disorders)	budesonide	budesonide 3mg gastro-resistant modified-release capsules
3546	corticosteroids (for respiratory conditions)	beclometasone dipropionate	qvar 50 inhaler (teva uk ltd)
30210	corticosteroids (for respiratory conditions)	beclometasone dipropionate	beclometasone 250micrograms/dose inhaler (teva uk

For peer review only

Product code	BNF header	Drug substance	Drug product
			ltd)
1552	corticosteroids (for respiratory conditions)	beclometasone dipropionate	becloforte easi-breathe 250microgram/actuation pressurised inhalation (allen & hanburys ltd)
5822	corticosteroids (for respiratory conditions)	fluticasone propionate	fluticasone 250micrograms/dose inhaler cfc free
35430	corticosteroids (for respiratory conditions)	beclometasone dipropionate	becodisks 200microgram with diskhaler (glaxosmithkline uk ltd)
42994	corticosteroids (for respiratory conditions)	fluticasone propionate	flixotide 250micrograms/dose accuhaler (glaxosmithkline uk ltd)
36290	corticosteroids (for respiratory conditions)	fluticasone propionate	flixotide 50microgram disks with diskhaler (glaxosmithkline uk ltd)
24898	corticosteroids (for respiratory conditions)	beclometasone dipropionate	spacehaler bdp 100microgram/actuation spacehaler (celltech pharma europe ltd)
35700	corticosteroids (for respiratory conditions)	fluticasone propionate	fluticasone propionate 500microgram inhalation powder blisters with device
3188	unknown		pulmicort complete 50 mcg inh
7788	corticosteroids (for respiratory conditions)	budesonide	budesonide 100micrograms/dose dry powder inhaler
35293	corticosteroids (for respiratory conditions)	beclometasone dipropionate	beclometasone 200microgram inhalation powder blisters with device
8251	unknown		pulmicort refil 50 mg inh
1259	corticosteroids (for respiratory conditions)	beclometasone dipropionate	beclometasone 200micrograms/dose inhaler
16525	corticosteroids (in chronic bowel disorders)	budesonide	budenofalk 3mg gastro-resistant capsules (dr. falk pharma uk ltd)
1680	corticosteroids (for respiratory conditions)	budesonide	pulmicort ls 50micrograms/dose inhaler (astrazeneca uk ltd)
10254	corticosteroids (for respiratory conditions)	mometasone furoate	mometasone 400micrograms/dose dry powder inhaler
20812	unknown		pulmicort refill
14524	corticosteroids (for respiratory conditions)	beclometasone dipropionate	bdp 250microgram/actuation spacehaler (celltech pharma europe ltd)
2992	corticosteroids (for respiratory conditions)	beclometasone dipropionate	beclazone 50 inhaler (teva uk ltd)
7602	corticosteroids (for respiratory conditions)	fluticasone propionate	fluticasone 50microgram disc
16305	corticosteroids (for respiratory conditions)	fluticasone propionate	flixotide 2mg/2ml nebules (glaxosmithkline uk ltd)
14757	corticosteroids (for respiratory conditions)	beclometasone dipropionate	pulvinal beclometasone dipropionate 100micrograms/dose dry powder inhaler (chiesi ltd)
909	unknown	budesonide	budesonide 200micrograms/dose inhaler

For peer review only

Product code	BNF header	Drug substance	Drug product
1959	corticosteroids (for respiratory conditions)	budesonide	pulmicort 0.5mg respules (astrazeneca uk ltd)
1861	corticosteroids (for respiratory conditions)	beclometasone dipropionate	aerobec 100 autohaler (meda pharmaceuticals ltd)
16018	corticosteroids (for respiratory conditions)	mometasone furoate	mometasone 200micrograms/dose dry powder inhaler
35631	corticosteroids (for respiratory conditions)	budesonide	budelin novolizer 200micrograms/dose inhalation powder (meda pharmaceuticals ltd)
60937	corticosteroids (for respiratory conditions)	budesonide	pulmicort 200 turbohaler (dowelhurst ltd)
50037	corticosteroids (for respiratory conditions)	budesonide	pulmicort 0.5mg respules (waymade healthcare plc)
19389	corticosteroids (for respiratory conditions)	beclometasone dipropionate	asmabec 50microgram/actuation spacehaler (celltech pharma europe ltd)
2282	corticosteroids (for respiratory conditions)	fluticasone propionate	fluticasone propionate 500micrograms/dose dry powder inhaler
18848	corticosteroids (for respiratory conditions)	beclometasone dipropionate	qvar 100micrograms/dose easi-breathe inhaler (teva uk ltd)
2440	corticosteroids (for respiratory conditions)	fluticasone propionate	flixotide accuhaler 500 500microgram/inhalation inhalation powder (allen & hanburys ltd)
23741	corticosteroids (for respiratory conditions)	budesonide	novolizer budesonide 200microgram/actuation pressurised inhalation (meda pharmaceuticals ltd)
3988	unknown		flixotide diskhaler-community pack 100 mcg
35724	corticosteroids (for respiratory conditions)	budesonide	budelin novolizer 200micrograms/dose inhalation powder refill (meda pharmaceuticals ltd)
1380	corticosteroids (in chronic bowel disorders)	budesonide	entocort cr 3mg capsules (astrazeneca uk ltd)
56144	corticosteroids (in chronic bowel disorders)	budesonide	budenofalk 9mg gastro-resistant granules sachets (dr. falk pharma uk ltd)
56475	corticosteroids (for respiratory conditions)	fluticasone propionate	flixotide 50micrograms/dose accuhaler (sigma pharmaceuticals plc)
40057	corticosteroids (for respiratory conditions)	budesonide	pulmicort 200micrograms/dose inhaler cfc free (astrazeneca uk ltd)
1236	corticosteroids (for respiratory conditions)	beclometasone dipropionate	becloforte 250micrograms/dose inhaler (glaxosmithkline uk ltd)
11497	corticosteroids (for respiratory conditions)	beclometasone dipropionate	beclometasone 400micrograms/dose dry powder inhaler
35408	corticosteroids (for respiratory conditions)	beclometasone dipropionate	becodisks 100microgram (glaxosmithkline uk ltd)
34315	corticosteroids (for respiratory conditions)	beclometasone dipropionate	beclometasone 250microgram/actuation inhalation powder (actavis uk ltd)

For peer review only

Product code	BNF header	Drug substance	Drug product
956	corticosteroids (for respiratory conditions)	budesonide	pulmicort 200 turbohaler (astrazeneca uk ltd)
18394	corticosteroids (for respiratory conditions)	beclometasone dipropionate	bdp 50microgram/actuation spacehaler (celltech pharma europe ltd)
14321	corticosteroids (for respiratory conditions)	beclometasone dipropionate	beclometasone 200micrograms/dose inhaler cfc free
11732	corticosteroids (for respiratory conditions)	beclometasone dipropionate	beclometasone 50micrograms/dose breath actuated inhaler cfc free
3993	corticosteroids (for respiratory conditions)	beclometasone dipropionate	filair forte 250micrograms/dose inhaler (meda pharmaceuticals ltd)
35225	corticosteroids (for respiratory conditions)	fluticasone propionate	flixotide 100microgram disks with diskhaler (glaxosmithkline uk ltd)
5521	corticosteroids (for respiratory conditions)	beclometasone dipropionate	beclometasone 200micrograms/dose dry powder inhaler
4601	corticosteroids (for respiratory conditions)	beclometasone dipropionate	asmabec 100 clickhaler (focus pharmaceuticals ltd)
16148	corticosteroids (for respiratory conditions)	beclometasone dipropionate	clenil modulite 250micrograms/dose inhaler (chiesi ltd)
2892	corticosteroids (for respiratory conditions)	beclometasone dipropionate	becloforte 400microgram disks (glaxosmithkline uk ltd)
35071	corticosteroids (for respiratory conditions)	beclometasone dipropionate	becodisks 200microgram (glaxosmithkline uk ltd)
35510	corticosteroids (for respiratory conditions)	budesonide	budesonide 200micrograms/dose dry powder inhalation cartridge with device
14567	corticosteroids (for respiratory conditions)	beclometasone dipropionate	asmabec 250 clickhaler (focus pharmaceuticals ltd)
56498	corticosteroids (for respiratory conditions)	budesonide	pulmicort 200 turbohaler (waymade healthcare plc)
24660	unknown		betamethasone valerate
51997	corticosteroids (in chronic bowel disorders)	budesonide	budesonide 9mg gastro-resistant granules sachets
1734	corticosteroids (for respiratory conditions)	beclometasone dipropionate	beclometasone 100micrograms/dose breath actuated inhaler
36090	corticosteroids (for respiratory conditions)	fluticasone propionate	flixotide 100microgram disks (glaxosmithkline uk ltd)
5975	corticosteroids (for respiratory conditions)	fluticasone propionate	fluticasone 125micrograms/dose inhaler cfc free
19401	corticosteroids (for respiratory conditions)	beclometasone dipropionate	beclometasone 250micrograms/actuation inhaler and compact spacer
895	corticosteroids (for respiratory conditions)	beclometasone dipropionate	beclazone 100 easi-breathe inhaler (teva uk ltd)
51815	corticosteroids (for respiratory conditions)	fluticasone propionate	flixotide 250micrograms/dose evohaler (waymade healthcare plc)
36021	corticosteroids (for respiratory conditions)	fluticasone propionate	fluticasone propionate 50microgram inhalation powder blisters with device

For peer review only

Product code	BNF header	Drug substance	Drug product
11198	corticosteroids (for respiratory conditions)	beclometasone dipropionate	beclometasons 50 micrograms/actuation vortex inhaler
50287	corticosteroids (for respiratory conditions)	beclometasone dipropionate	qvar 100 inhaler (de pharmaceuticals)
34428	corticosteroids (for respiratory conditions)	beclometasone dipropionate	beclometasone 50microgram/actuation inhalation powder (neo laboratories ltd)
26063	corticosteroids (for respiratory conditions)	beclometasone dipropionate	beclometasone 100micrograms/dose inhaler (teva uk ltd)
35602	corticosteroids (for respiratory conditions)	budesonide	budesonide 200micrograms/dose dry powder inhalation cartridge
30238	corticosteroids (for respiratory conditions)	beclometasone dipropionate	beclometasone 50microgram/actuation pressurised inhalation (approved prescription services ltd)
14700	corticosteroids (for respiratory conditions)	budesonide	budesonide 400micrograms/actuation inhaler
38	corticosteroids (for respiratory conditions)	beclometasone dipropionate	beclometasone 100micrograms/dose inhaler
13815	corticosteroids (for respiratory conditions)	beclometasone dipropionate	beclazone 100microgram/actuation inhalation powder (actavis uk ltd)
947	corticosteroids (for respiratory conditions)	budesonide	budesonide 50micrograms/actuation refill canister
10321	unknown	budesonide	budesonide 400microgram inhalation powder capsules
1426	corticosteroids (for respiratory conditions)	fluticasone propionate	flixotide 500microgram disc (allen & hanburys ltd)
3150	corticosteroids (for respiratory conditions)	beclometasone dipropionate	beclometasone 100micrograms/actuation extrafine particle cfc free inhaler
35113	corticosteroids (for respiratory conditions)	beclometasone dipropionate	beclometasone 200microgram inhalation powder blisters
1424	corticosteroids (for respiratory conditions)	fluticasone propionate	flixotide 250microgram disc (allen & hanburys ltd)
4803	corticosteroids (for respiratory conditions)	beclometasone dipropionate	beclazone 250microgram/actuation inhalation powder (actavis uk ltd)
56493	corticosteroids (for respiratory conditions)	beclometasone dipropionate	qvar 50micrograms/dose easi-breathe inhaler (sigma pharmaceuticals plc)
61664	corticosteroids (for respiratory conditions)	beclometasone dipropionate	clenil modulite 250micrograms/dose inhaler (waymade healthcare plc)
35772	corticosteroids (for respiratory conditions)	fluticasone propionate	fluticasone propionate 100microgram inhalation powder blisters
3119	corticosteroids (for respiratory conditions)	beclometasone dipropionate	becloforte integra 250microgram/actuation inhaler with compact spacer (glaxo laboratories ltd)
5522	corticosteroids (for respiratory conditions)	beclometasone dipropionate	beclometasone 100micrograms/dose dry powder inhaler

For peer review only

Product code	BNF header	Drug substance	Drug product
25204	corticosteroids (for respiratory conditions)	beclometasone dipropionate	beclometasone 100micrograms/dose inhaler (a a h pharmaceuticals ltd)
7724	corticosteroids (for respiratory conditions)	betamethasone valerate	betamethasone valerate 100micrograms/actuation inhaler
3743	corticosteroids (for respiratory conditions)	beclometasone dipropionate	filair 50 inhaler (meda pharmaceuticals ltd)
1956	corticosteroids (for respiratory conditions)	budesonide	pulmicort 1mg respules (astrazeneca uk ltd)
56499	corticosteroids (for respiratory conditions)	fluticasone propionate	flixotide 500micrograms/dose accuhaler (waymade healthcare plc)
2125	corticosteroids (for respiratory conditions)	budesonide	pulmicort 200microgram refill canister (astrazeneca uk ltd)
36401	corticosteroids (for respiratory conditions)	fluticasone propionate	fluticasone propionate 250microgram inhalation powder blisters with device
4688	corticosteroids (for respiratory conditions)	fluticasone propionate	fluticasone 50microgram/actuation pressurised inhalation
3065	corticosteroids (for respiratory conditions)	betamethasone valerate	bextasol inhalation powder (allen & hanburys ltd)
35118	corticosteroids (for respiratory conditions)	beclometasone dipropionate	becodisks 400microgram with diskhaler (glaxosmithkline uk ltd)
1518	corticosteroids (for respiratory conditions)	fluticasone propionate	flixotide 50microgram/actuation inhalation powder (allen & hanburys ltd)
5309	corticosteroids (for respiratory conditions)	fluticasone propionate	flixotide 50micrograms/dose evohaler (glaxosmithkline uk ltd)
47225	unknown	budesonide	budesonide 9mg gastro-resistant granules sachets
30649	corticosteroids (for respiratory conditions)	budesonide	easyhaler budesonide 400micrograms/dose dry powder inhaler (orion pharma (uk) ltd)
35580	corticosteroids (for respiratory conditions)	beclometasone dipropionate	beclometasone 100microgram inhalation powder blisters with device
41412	corticosteroids (for respiratory conditions)	beclometasone dipropionate	beclometasone 400micrograms/actuation inhaler
20825	corticosteroids (for respiratory conditions)	beclometasone dipropionate	spacehaler bdp 250microgram/actuation spacehaler (celltech pharma europe ltd)
35461	corticosteroids (for respiratory conditions)	fluticasone propionate	flixotide 250microgram disks with diskhaler (glaxosmithkline uk ltd)
42985	corticosteroids (for respiratory conditions)	fluticasone propionate	flixotide 50micrograms/dose accuhaler (glaxosmithkline uk ltd)
9921	corticosteroids (for respiratory conditions)	beclometasone dipropionate	beclometasone 100micrograms/dose breath actuated inhaler cfc free

For peer review only

Product code	BNF header	Drug substance	Drug product
56474	corticosteroids (for respiratory conditions)	fluticasone propionate	flixotide 125micrograms/dose evohaler (de pharmaceuticals)
35986	corticosteroids (for respiratory conditions)	fluticasone propionate	flixotide 50microgram disks (glaxosmithkline uk ltd)
11149	glucocorticoid therapy	betamethasone	betnelan 500microgram tablets (focus pharmaceuticals ltd)
1243	corticosteroids (for respiratory conditions)	beclometasone dipropionate	beclazone 250 easi-breathe inhaler (teva uk ltd)
31774	corticosteroids (for respiratory conditions)	beclometasone dipropionate	beclometasone 50micrograms/dose inhaler (generics (uk) ltd)
56477	corticosteroids (for respiratory conditions)	fluticasone propionate	flixotide 100micrograms/dose accuhaler (waymade healthcare plc)
2600	corticosteroids (for respiratory conditions)	beclometasone dipropionate	beclometasone 250micrograms/dose breath actuated inhaler
16151	corticosteroids (for respiratory conditions)	beclometasone dipropionate	clenil modulite 200micrograms/dose inhaler (chiesi ltd)
1100	corticosteroids (for respiratory conditions)	beclometasone dipropionate	beclazone 100 inhaler (teva uk ltd)
1951	corticosteroids (for respiratory conditions)	beclometasone dipropionate	becodisks 400microgram disc (allen & hanburys ltd)
7948	corticosteroids (for respiratory conditions)	fluticasone propionate	fluticasone propionate 250micrograms/dose dry powder inhaler
21482	corticosteroids (for respiratory conditions)	beclometasone dipropionate	beclometasone 100micrograms/dose inhaler (generics (uk) ltd)
9477	corticosteroids (for respiratory conditions)	beclometasone dipropionate	asmabec 100microgram/actuation spacehaler (celltech pharma europe ltd)
27679	corticosteroids (for respiratory conditions)	beclometasone dipropionate	beclometasone 100microgram/actuation pressurised inhalation (approved prescription services ltd)
35652	corticosteroids (for respiratory conditions)	beclometasone dipropionate	beclometasone 100microgram inhalation powder blisters
46157	corticosteroids (for respiratory conditions)	beclometasone dipropionate	beclometasone 200 cyclocaps (teva uk ltd)
56484	corticosteroids (for respiratory conditions)	fluticasone propionate	flixotide 250micrograms/dose accuhaler (waymade healthcare plc)
3442	unknown		pulmicort complete 200 mcg inh
28761	corticosteroids (for respiratory conditions)	beclometasone dipropionate	spacehaler bdp 50microgram/actuation spacehaler (celltech pharma europe ltd)
5992	corticosteroids (for respiratory conditions)	beclometasone dipropionate	beclometasone 50micrograms/dose dry powder inhaler
454	corticosteroids (for respiratory conditions)	budesonide	pulmicort 200microgram inhaler (astrazeneca uk ltd)

For peer review only

Product code	BNF header	Drug substance	Drug product
3220	corticosteroids (for respiratory conditions)	beclometasone dipropionate	qvar 50 autohaler (teva uk ltd)
18537	unknown	budesonide	budesonide 200microgram inhalation powder capsules
57555	corticosteroids (for respiratory conditions)	fluticasone propionate	flixotide 125micrograms/dose evohaler (dowelhurst ltd)
48709	corticosteroids (for respiratory conditions)	beclometasone dipropionate	qvar 100micrograms/dose easi-breathe inhaler (sigma pharmaceuticals plc)
16584	corticosteroids (for respiratory conditions)	beclometasone dipropionate	beclometasone 50micrograms/dose inhaler cfc free
35392	corticosteroids (for respiratory conditions)	fluticasone propionate	flixotide 500microgram disks with diskhaler (glaxosmithkline uk ltd)

For peer review only

FP_SAL codes

Product code	BNF header	Drug substance	Drug product
638	Selective Beta 2 Agonists/Corticosteroids (for Respiratory Conditions)	Fluticasone propionate/Salmeterol xinafoate	Seretide 250 Accuhaler (GlaxoSmithKline UK Ltd)
665	Selective Beta 2 Agonists/Corticosteroids (for Respiratory Conditions)	Salmeterol xinafoate/Fluticasone propionate	Seretide 100 Accuhaler (GlaxoSmithKline UK Ltd)
3666	Selective Beta 2 Agonists/Corticosteroids (for Respiratory Conditions)	Salmeterol xinafoate/Fluticasone propionate	Seretide 500 Accuhaler (GlaxoSmithKline UK Ltd)
5143	Selective Beta 2 Agonists/Corticosteroids (for Respiratory Conditions)	Fluticasone propionate/Salmeterol xinafoate	Seretide 50 Evohaler (GlaxoSmithKline UK Ltd)
5161	Selective Beta 2 Agonists/Corticosteroids (for Respiratory Conditions)	Fluticasone propionate/Salmeterol xinafoate	Seretide 125 Evohaler (GlaxoSmithKline UK Ltd)
5172	Selective Beta 2 Agonists/Corticosteroids (for Respiratory Conditions)	Fluticasone propionate/Salmeterol xinafoate	Seretide 250 Evohaler (GlaxoSmithKline UK Ltd)
5558	Selective Beta 2 Agonists/Corticosteroids (for Respiratory Conditions)	Salmeterol Xinafoate/Fluticasone Propionate	Salmeterol 50micrograms with fluticasone 500micrograms CFC free inhaler
5864	Selective Beta 2 Agonists/Corticosteroids (for Respiratory Conditions)	Salmeterol Xinafoate/Fluticasone Propionate	Salmeterol 25micrograms with fluticasone 250micrograms CFC free inhaler
5942	Selective Beta 2 Agonists/Corticosteroids (for Respiratory Conditions)	Salmeterol Xinafoate/Fluticasone Propionate	Salmeterol 50micrograms with fluticasone 250micrograms CFC free inhaler
6569	Selective Beta 2 Agonists/Corticosteroids (for Respiratory Conditions)	Salmeterol Xinafoate/Fluticasone Propionate	Salmeterol 25micrograms with fluticasone 125micrograms CFC free inhaler
6616	Selective Beta 2 Agonists/Corticosteroids (for Respiratory Conditions)	Salmeterol Xinafoate/Fluticasone Propionate	Salmeterol 25micrograms with fluticasone 50micrograms CFC free inhaler
6938	Selective Beta 2 Agonists/Corticosteroids (for Respiratory Conditions)	Salmeterol Xinafoate/Fluticasone Propionate	Salmeterol 50micrograms with fluticasone 100micrograms dry powder inhaler
11410	Selective Beta 2 Agonists/Corticosteroids (for Respiratory Conditions)	Salmeterol xinafoate/Fluticasone propionate	Fluticasone propionate 500micrograms/dose / Salmeterol 50micrograms/dose dry powder inhaler
11588	Selective Beta 2 Agonists/Corticosteroids (for Respiratory Conditions)	Fluticasone propionate/Salmeterol xinafoate	Fluticasone 125micrograms/dose / Salmeterol 25micrograms/dose inhaler CFC free
11618	Selective Beta 2 Agonists/Corticosteroids (for Respiratory Conditions)	Fluticasone propionate/Salmeterol xinafoate	Fluticasone 250micrograms/dose / Salmeterol 25micrograms/dose inhaler CFC free
12994	Selective Beta 2 Agonists/Corticosteroids (for Respiratory Conditions)	Fluticasone propionate/Salmeterol xinafoate	Fluticasone 50micrograms/dose / Salmeterol 25micrograms/dose inhaler CFC free
13040	Selective Beta 2 Agonists/Corticosteroids	Fluticasone propionate/Salmeterol xinafoate	Fluticasone propionate 250micrograms/dose /

For peer review only

Product code	BNF header	Drug substance	Drug product
	(for Respiratory Conditions)		Salmeterol 50micrograms/dose dry powder inhaler
13273	Selective Beta 2 Agonists/Corticosteroids (for Respiratory Conditions)	Salmeterol xinafoate/Fluticasone propionate	Fluticasone propionate 100micrograms/dose / Salmeterol 50micrograms/dose dry powder inhaler
48739	Selective Beta 2 Agonists/Corticosteroids (for Respiratory Conditions)	Fluticasone propionate/Salmeterol xinafoate	Seretide 250 Evohaler (DE Pharmaceuticals)
49000	Selective Beta 2 Agonists/Corticosteroids (for Respiratory Conditions)	Fluticasone propionate/Salmeterol xinafoate	Seretide 250 Evohaler (Waymade Healthcare Plc)
50560	Selective Beta 2 Agonists/Corticosteroids (for Respiratory Conditions)	Fluticasone propionate/Salmeterol xinafoate	Seretide 250 Accuhaler (Sigma Pharmaceuticals Plc)
50689	Selective Beta 2 Agonists/Corticosteroids (for Respiratory Conditions)	Fluticasone propionate/Formoterol fumarate dihydrate	Flutiform 50micrograms/dose / 5micrograms/dose inhaler (Napp Pharmaceuticals Ltd)
50886	Selective Beta 2 Agonists/Corticosteroids (for Respiratory Conditions)	Fluticasone propionate/Salmeterol xinafoate	Seretide 250 Evohaler (Stephar (U.K.) Ltd)
51027	Selective Beta 2 Agonists/Corticosteroids (for Respiratory Conditions)	Fluticasone propionate/Salmeterol xinafoate	Seretide 125 Evohaler (DE Pharmaceuticals)
51151	Selective Beta 2 Agonists/Corticosteroids (for Respiratory Conditions)	Fluticasone propionate/Salmeterol xinafoate	Seretide 125 Evohaler (Lexon (UK) Ltd)
51270	Selective Beta 2 Agonists/Corticosteroids (for Respiratory Conditions)	Fluticasone propionate/Formoterol fumarate dihydrate	Fluticasone 50micrograms/dose / Formoterol 5micrograms/dose inhaler CFC free
51394	Selective Beta 2 Agonists/Corticosteroids (for Respiratory Conditions)	Salmeterol xinafoate/Fluticasone propionate	Seretide 500 Accuhaler (Waymade Healthcare Plc)
51593	Selective Beta 2 Agonists/Corticosteroids (for Respiratory Conditions)	Salmeterol xinafoate/Fluticasone propionate	Seretide 500 Accuhaler (DE Pharmaceuticals)
51861	Selective Beta 2 Agonists/Corticosteroids (for Respiratory Conditions)	Salmeterol xinafoate/Fluticasone propionate	Seretide 500 Accuhaler (Mawdsley-Brooks & Company Ltd)
51909	Selective Beta 2 Agonists/Corticosteroids (for Respiratory Conditions)	Fluticasone propionate/Salmeterol xinafoate	Seretide 250 Evohaler (Necessity Supplies Ltd)
53230	Selective Beta 2 Agonists/Corticosteroids (for Respiratory Conditions)	Fluticasone propionate/Salmeterol xinafoate	Seretide 250 Accuhaler (DE Pharmaceuticals)
53283	Selective Beta 2 Agonists/Corticosteroids (for Respiratory Conditions)	Salmeterol xinafoate/Fluticasone propionate	Seretide 100 Accuhaler (Waymade Healthcare Plc)
55411	Corticosteroids Used In Nasal Allergy/Antihistamines In Nasal Allergy	Fluticasone propionate/Azelastine hydrochloride	Fluticasone propionate 50micrograms/dose / Azelastine 137micrograms/dose nasal spray
55435	Corticosteroids Used In Nasal Allergy/Antihistamines In Nasal Allergy	Fluticasone propionate/Azelastine hydrochloride	Dymista 137micrograms/dose / 50micrograms/dose nasal spray (Meda Pharmaceuticals Ltd)

For peer review only

Product code	BNF header	Drug substance	Drug product
55677	Selective Beta 2 Agonists/Corticosteroids (for Respiratory Conditions)	Salmeterol xinafoate/Fluticasone propionate	Seretide 500 Accuhaler (Lexon (UK) Ltd)
61280	Selective Beta 2 Agonists/Corticosteroids (for Respiratory Conditions)	Fluticasone propionate/Salmeterol xinafoate	Seretide 250 Accuhaler (Waymade Healthcare Plc)
62126	Selective Beta 2 Agonists/Corticosteroids (for Respiratory Conditions)	Salmeterol xinafoate/Fluticasone propionate	Seretide 100 Accuhaler (DE Pharmaceuticals)
63252	Selective Beta 2 Agonists/Corticosteroids (for Respiratory Conditions)	Fluticasone propionate/Salmeterol xinafoate	Seretide 250 Evohaler (Lexon (UK) Ltd)
63945	Selective Beta 2 Agonists/Corticosteroids (for Respiratory Conditions)	Fluticasone propionate/Salmeterol xinafoate	Seretide 250 Accuhaler (Lexon (UK) Ltd)
64372	Selective Beta 2 Agonists/Corticosteroids (for Respiratory Conditions)	Fluticasone propionate/Salmeterol xinafoate	Sirdupla 25micrograms/dose / 125micrograms/dose inhaler (Mylan Ltd)
64373	Selective Beta 2 Agonists/Corticosteroids (for Respiratory Conditions)	Fluticasone propionate/Salmeterol xinafoate	Sirdupla 25micrograms/dose / 250micrograms/dose inhaler (Mylan Ltd)
65117	Selective Beta 2 Agonists/Corticosteroids (for Respiratory Conditions)	Fluticasone propionate/Salmeterol xinafoate	Seretide 125 Evohaler (Mawdsley-Brooks & Company Ltd)

For peer review only

For peer review only

STROBE 2007 (v4) Statement—Checklist of items that should be included in reports of *cohort studies*

Section/Topic	Item #	Recommendation	Reported on page #
**Title and abstract**	1	(*a*) Indicate the study's design with a commonly used term in the title or the abstract	1
		(*b*) Provide in the abstract an informative and balanced summary of what was done and what was found	3 (note: is a protocol for an observational study so here and subsequently is reported *what is planned to be done* only)
**Introduction**			
Background/rationale	2	Explain the scientific background and rationale for the investigation being reported	5
Objectives	3	State specific objectives, including any prespecified hypotheses	6
**Methods**			
Study design	4	Present key elements of study design early in the paper	6
Setting	5	Describe the setting, locations, and relevant dates, including periods of recruitment, exposure, follow-up, and data collection	6
Participants	6	(*a*) Give the eligibility criteria, and the sources and methods of selection of participants. Describe methods of follow-up	7 - 10
		(*b*) For matched studies, give matching criteria and number of exposed and unexposed	7 - 10
Variables	7	Clearly define all outcomes, exposures, predictors, potential confounders, and effect modifiers. Give diagnostic criteria, if applicable	13 - 14
Data sources/ measurement	8*	For each variable of interest, give sources of data and details of methods of assessment (measurement). Describe comparability of assessment methods if there is more than one group	6
Bias	9	Describe any efforts to address potential sources of bias	14 - 16
Study size	10	Explain how the study size was arrived at	14
Quantitative variables	11	Explain how quantitative variables were handled in the analyses. If applicable, describe which groupings were chosen and why	11

For peer review only

Statistical methods	12	(*a*) Describe all statistical methods, including those used to control for confounding	14 - 16
		(*b*) Describe any methods used to examine subgroups and interactions	14 - 16
		(*c*) Explain how missing data were addressed	16
		(*d*) If applicable, explain how loss to follow-up was addressed	NA
		(*e*) Describe any sensitivity analyses	14 - 16
**Results**			***NA (is a protocol for an observational study)***
Participants	13*	(a) Report numbers of individuals at each stage of study—eg numbers potentially eligible, examined for eligibility, confirmed eligible, included in the study, completing follow-up, and analysed	
		(b) Give reasons for non-participation at each stage	
		(c) Consider use of a flow diagram	
Descriptive data	14*	(a) Give characteristics of study participants (eg demographic, clinical, social) and information on exposures and potential confounders	
		(b) Indicate number of participants with missing data for each variable of interest	
		(c) Summarise follow-up time (eg, average and total amount)	
Outcome data	15*	Report numbers of outcome events or summary measures over time	
Main results	16	(*a*) Give unadjusted estimates and, if applicable, confounder-adjusted estimates and their precision (eg, 95% confidence interval). Make clear which confounders were adjusted for and why they were included	
		(*b*) Report category boundaries when continuous variables were categorized	
		(*c*) If relevant, consider translating estimates of relative risk into absolute risk for a meaningful time period	
Other analyses	17	Report other analyses done—eg analyses of subgroups and interactions, and sensitivity analyses	
**Discussion**			***NA (is a protocol for an observational study)***
Key results	18	Summarise key results with reference to study objectives	
**Limitations**			***NA (is a protocol for an observational study)***
Interpretation	20	Give a cautious overall interpretation of results considering objectives, limitations, multiplicity of analyses, results from similar studies, and other relevant evidence	

For peer review only

Generalisability	21	Discuss the generalisability (external validity) of the study results	
**Other information**			
Funding	22	Give the source of funding and the role of the funders for the present study and, if applicable, for the original study on which the present article is based	2

*Give information separately for cases and controls in case-control studies and, if applicable, for exposed and unexposed groups in cohort and cross-sectional studies.

Note: An Explanation and Elaboration article discusses each checklist item and gives methodological background and published examples of transparent reporting. The STROBE checklist is best used in conjunction with this article (freely available on the Web sites of PLoS Medicine at http://www.plosmedicine.org/, Annals of Internal Medicine at http://www.annals.org/, and Epidemiology at http://www.epidem.com/). Information on the STROBE Initiative is available at www.strobe-statement.org.

For peer review only

BMJ Open

Real world effects of medications for chronic obstructive pulmonary disease: protocol for a UK population-based non-interventional cohort study with validation against randomised trial results

Journal:	*BMJ Open*
Manuscript ID	bmjopen-2017-019475.R1
Article Type:	Protocol
Date Submitted by the Author:	05-Dec-2017
Complete List of Authors:	Wing, Kevin; London School of Hygiene and Tropical Medicine,<br>Williamson, Elizabeth; London School of Hygiene and Tropical Medicine,<br>Carpenter, James; London School of Hygiene and Tropical Medicine<br>Wise, Lesley; London School of Hygiene and Tropical Medicine<br>Schneeweiss, Sebastian; Brigham and Womens' Hospital, Harvard Medical School<br>Smeeth, Liam; London School of Hygiene and Tropical Medicine<br>Quint, Jennifer; Imperial College London<br>Douglas, Ian; London School of Hygiene and Tropical Medicine
**Primary Subject Heading**:	Epidemiology
Secondary Subject Heading:	Evidence based practice, Research methods, Respiratory medicine, Public health, Pharmacology and therapeutics
Keywords:	Chronic airways disease < THORACIC MEDICINE, Epidemiology < THORACIC MEDICINE, CLINICAL PHARMACOLOGY, EPIDEMIOLOGY, PRIMARY CARE, PUBLIC HEALTH

SCHOLARONE™ Manuscripts

TITLE PAGE

Title: Real world effects of medications for chronic obstructive pulmonary disease: protocol for a UK population-based non-interventional cohort study with validation against randomised trial results

Authors:

Kevin Wing[1*]

Elizabeth Williamson[2]

James Carpenter[2]

Lesley Wise[1]

Sebastian Schneeweiss[3,4]

Liam Smeeth[1]

Jennifer Quint[5]

Ian Douglas[1]

[1]Department of Non-communicable Disease Epidemiology, Faculty of Epidemiology and Population Health, London School of Hygiene and Tropical Medicine, London, UK

[2]Department of Medical Statistics, Faculty of Epidemiology and Population Health, London School of Hygiene and Tropical Medicine, London, UK

[3]Department of Epidemiology, Harvard Medical School.

[4]Division of Pharmacoepidemiology and Pharmacoeconomics, Department of Medicine, Brigham & Women's Hospital

[5]National Heart and Lung Institute, Imperial College London, London, United Kingdom

***Submitting and corresponding author:** Kevin Wing (kevin.wing@lshtm.ac.uk), London School of Hygiene and Tropical Medicine, Keppel Street, London WC1E 7HT. Tel: +44 20 7636 8636, Fax: +44 20 7436 5389

For peer review only

Funding statement

This work was supported by NIHR grant number 15/80/28

Competing interests statement

Dr Wing and Dr Williamson declare no competing interests.

Professor Carpenter is funded 60% by a grant from the MRC, via a secondment to the MRC Clinical Trials Unit. The remaining funding is a combination of HEFCE and small contributions from various NIHR, ESRC, MRC and EU funds. He undertakes methodological consultancy work for Novartis and GlaxoSmithKline, and has given missing data courses for GlaxoSmithKline, Bayer and Boehringer. Professor Smeeth reports grants from Wellcome, MRC, NIHR, BHF, Diabetes UK, ESRC and the EU; grants and personal fees for advisory work from GSK, and personal fees for advisory work from AstraZeneca. He is a Trustee of the British Heart Foundation.

Dr Wise is an independent consultant to the pharmaceutical industry and is employed to provide advice by a number of different companies none of which is involved in this therapeutic area.

Professor Schneeweiss is a consultant to WHISCON, LLC and to Aetion, Inc., a software manufacturer of which he also owns equity. He is principal investigator of investigator-initiated grants to the Brigham and Women's Hospital from Bayer, Genentech and Boehringer Ingelheim unrelated to the topic of this study. He does not receive personal fees from biopharmaceutical companies.

Professor Smeeth reports grants from Wellcome, MRC, NIHR, BHF, Diabetes UK, ESRC and the EU; grants and personal fees for advisory work from GSK, and personal fees for advisory work from AstraZeneca. He is a Trustee of the British Heart Foundation.

Dr Quint's research group has received funding from MRC, Wellcome, BLF, GSK, BI, AZ and Insmed for other projects, none of which relate to this work.

Dr Douglas is funded by an unrestricted grant from, has consulted for and holds stock in GlaxoSmithKline.

Word count (not including abstract, tables, figures, or footnotes): 4386

Statement about prior postings and presentations: This work has not been published elsewhere.

For peer review only

Abstract

Introduction

Chronic obstructive pulmonary disease (COPD) is a progressive disease affecting 3 million people in the UK, in which patients exhibit airflow obstruction that is not fully reversible. COPD treatment guidelines are largely informed by randomised controlled trial (RCT) results, but it is unclear if these findings apply to large patient populations not studied in trials. Non-interventional studies could be used to study patient groups excluded from trials, but the use of these studies to estimate treatment effectiveness is in its infancy. In this study we will use individual trial data to validate non-interventional methods for assessing COPD treatment effectiveness, before applying these methods to analysing treatment effectiveness within people excluded from, or under-represented in COPD trials.

Methods and analysis

Using individual patient data from the landmark COPD TORCH trial and validated methods for detecting COPD and exacerbations in routinely collected primary care data, we will assemble a cohort in the UK Clinical Practice Research Datalink (selecting people from between the dates of 1st January 2004 and 1st January 2017) with similar characteristics to TORCH participants and test whether non-interventional data can generate comparable results to trials, using cohort methodology with propensity score techniques to adjust for potential confounding. We will then use the methodological template we have developed to determine risks and benefits of COPD treatments in people excluded from TORCH. Outcomes are pneumonia, COPD exacerbation, mortality and time to treatment change. Groups to be studied include the elderly (>80 years), people with substantial comorbidity, people with and without underlying cardiovascular disease and people with mild COPD.

Ethics and dissemination

Ethical approval has been granted by the LSHTM Medicines Ethics Committee (Ref: 11997). The study is under review by the Independent Scientific Advisory Committee (ISAC) of the UK Medicines and Healthcare Products Regulatory Agency (MHRA). An application to use the TORCH trial data made to clinicalstudydatarequest.com has been approved. In addition to scientific publications, dissemination methods will be developed based on discussions with COPD patient groups.

For peer review only

Strengths and limitations of this study

Strengths

- Large cohort study
- Use of validated methods for detecting COPD within the Clinical Practice Research Datatlink
- Use of randomised controlled trial (RCT) individual patient data to assess ability of non-interventional methods to detect COPD treatment effects within an RCT-analagous population

Limitations

- Adherence to medication will need to be assessed based on proxy variables (e.g. time covered by prescription)

For peer review only

Introduction

Background and rationale

Chronic obstructive pulmonary disease (COPD) affects 3 million people in the UK.[1] The most common cause is smoking, and patients exhibit airflow obstruction that is not fully reversible. The disease is progressive, with declining lung function and a worsening of symptoms. Most troublesome are acute exacerbations manifested as a sudden worsening of symptoms e.g. severe coughing, shortness of breath and chest congestion, requiring urgent treatment, and possibly hospitalisation. Whilst smoking cessation remains the most effective intervention, the rate of exacerbation can be reduced by regular medication such as combination long-acting beta-agonists (LABA) and inhaled corticosteroids (ICS) or long-acting muscarinic antagonists (LAMAs).[2,3]

COPD treatment guidelines are largely informed by randomised controlled trial (RCT) results,[4] but it is not clear if these findings apply to large patient populations not studied in trials. Fluticasone propionate + salmeterol (FP/SAL) is a LABA/ICS combination and is one of the most widely used COPD treatments. It was studied in large randomised trials (e.g. the TORCH trial),[2] but the effects of treatment in important patient groups who were not studied are unknown. Some were excluded from trials (e.g. those >80 years and those with substantial comorbidity) while others are under represented (e.g. people with mild COPD),[2,5] meaning conclusions about these groups are difficult to make.

Whilst the conduct of non-interventional studies (sometimes also referred to as “observational studies”) to investigate possible drug harms is well established, the use of these studies to estimate treatment effectiveness is in its infancy. Issues of treatment channelling and indication bias mean that measuring the intended benefit of a treatment is beset with difficulties. Over the next few years we will see more non-interventional studies of drug effectiveness emerging due to recent legislation requiring pharmaceutical companies to study the real world effects of medications;[6,7] however, rigorous, validated methodology is needed to translate these complex data into reliable evidence. For example, the availability of anonymised individual patient data from randomised controlled trials provides the potential for “RCT-analogous” cohorts to be selected from non-interventional data sources (by matching patient records from non-interventional data to the RCT patient records on key characteristics). If subsequent analysis of a non-interventional RCT-analogous cohort generates results that are similar to those generated by the reference RCT, one could be confident in the validity of the results, and in the non-interventional methods used to obtain these results.

In this study we will use TORCH[2] individual trial data to validate non-interventional methods for assessing COPD treatment effectiveness, before going on to apply these methods to analysing treatment effectiveness within people excluded from or underrepresented in the TORCH trial. Non-

For peer review only

interventional data will be obtained from the UK Clinical Practice Research Datalink (linked to the Hospital Episodes Statistics database).[8] The results we generate will aid patients, prescribers and policy makers in deciding the most appropriate treatment for COPD for all types of patients. The approach used can also provide a template for treatment effectiveness research using non-interventional data with inbuilt validation against a randomised trial.

Aims and objectives

The aims of our study are (1) to measure the association between treatments for COPD and COPD exacerbation rate, mortality, pneumonia, and time to treatment change amongst patients not included in randomised clinical trials for COPD treatments and (2) to develop a methodological framework with in built validation against RCT data, for using non-interventional electronic health records (EHR) to answer questions about drug treatment effects (i.e. both benefits and risks).

Specific objectives are to: (1) validate methods for measuring COPD medication effectiveness in EHR data by comparing with trial results; (2) use EHR data to measure COPD medication effectiveness in patients excluded from trials (most importantly those >80 years or with substantial comorbidity) and (3) determine COPD treatment effectiveness in an under-studied disease stage (mild COPD).

Methods and analysis

Figure 1 provides a high-level overview of the study, detailing each objective and data source used, and showing how existing RCT data will be used in Objective 1 in order to validate methods for analysing COPD in routinely collected electronic health data that will then be applied to unanswered questions in Objective 2 and 3.

Study design

We have chosen a cohort study design as it will allow us to measure the effects of prescribing different treatments for COPD on future outcomes in different types of patients. Eligibility criteria for cohort entry will vary between objectives (detailed in the selection of participants section below).

Setting/data sources

For peer review only

Patient data used in this study will be obtained from two different sources: the TORCH randomised trial and the UK Clinical Practices Research Datalink (CPRD) (linked to Hospital Episodes Statistics – HES – data).

TORCH

TORCH was a placebo controlled randomised trial of the combined inhaler fluticasone propionate (FP) + salmeterol (SAL) (FP/SAL) for the treatment of COPD, published in 2007. Patients were randomised to receive FP/SAL, FP alone, SAL alone or placebo and the primary comparison of interest was between FP/SAL and placebo.[2] Key outcomes were expected benefits (rate of COPD exacerbation and mortality) and an expected harm due to the immunosuppressive action of the corticosteroid FP (pneumonia). Whilst findings for the primary endpoint of mortality were null, this was thought to be due to poor statistical power as a result of a lower than anticipated mortality rate. Nonetheless, a lower rate of exacerbations was seen with FP/SAL, and a higher rate of pneumonia was observed. As one of the largest trials in COPD, and with three year follow up, TORCH is a landmark study, providing a validation point for our study. We will obtain individual patient data from the TORCH study via www.clinicalstudydatarequest.com for use in Objective 1 (see selection of participants section below)

CPRD

The CPRD is a very large database of prospectively collected, anonymised UK population-based electronic health records. CPRD primary care records comprise ~8-10% of the UK population and contain comprehensive information on clinical diagnoses, prescribing, referrals, tests and demographic/lifestyle factors.[8] In order to contribute to the database, general practices and other health centres must meet prespecified standards for research-quality data (i.e. be "up to standard"). Data quality/validity are therefore high and the data are nationally representative.[8,9] A patient starts contributing follow-up time to the database at the date they join an "up to standard" practice (or the date that their practice starts contributing up to standard data), and stop contributing follow-up time on either their death date, their transfer out date (the date that they leave the database due to reasons other than death) or on the last collection date for their practice. Linkage between the primary care records in CPRD and hospital episode statistics (HES) is well established for >60% of practices in the CPRD, providing a data set augmented with detailed secondary care diagnostic and procedural records. Algorithms have been established to identify COPD, COPD exacerbations and pneumonia (both hospital and primary care managed) in CPRD/HES linked data (including validated algorithms for COPD and exacerbations).[10–12] See supplementary materials for a high-level overview of these algorithms.

For peer review only

Figure 1: Overview of study objectives and sources of data for the COPD real-world medicines effects study (RCT=randomised controlled trial, CPRD= the UK Clinical Practice Research Datalink, FP/SAL=Fluticasone propionate + salmeterol)

[FIGURE 1 HERE]

A. Work performed by others prior to this study
Of the total population of people with COPD, only a subset are included in RCTs of COPD treatments, based on the RCT inclusion/exclusion criteria. The RCT generates results that inform clinical practice, and the anonymised raw data for the study can be made available to other researchers via the Clinical Study Data Request website. For this study, the specific COPD treatment-RCT of interest is the TORCH trial,[2] investigating the effect of FP/SAL on COPD exacerbations.

B. Work to be performed as part of this study

1. Objective 1

A cohort of TORCH (RCT)-analogous patients will be selected from the UK Clinical Practice Research Datalink (CPRD), by matching people with COPD within CPRD to the records of people included in the trial. An analysis of the effect of FP/SAL on COPD exacerbations will then be performed on this TORCH-analagous CPRD cohort. If the results obtained are comparable to those obtained in the TORCH trial itself, this will serve as a validation step, showing that data from the non-inverventional ("real-world") CPRD source can reliably be used to study COPD treatment effects.

2. Objective 2

The validated analysis techniques used for Objective 1 will then be used to study people in CPRD who would not have been eligible for inclusion in an RCT due to their age and the presence of other comorbidities, and for whom the effect of FP/SAL is currently unknown.

3. Objective 3

The validated analysis techniques will then be used to study people with mild COPD only, who have been under-represented in RCTs, and for whom the effect of COPD treatments is unclear.

For peer review only

Selection of participants

Participants will be selected from the CPRD from between the dates of 1st January 2004 and 1st January 2017. All patients will need to have been registered with an up-to standard practice for at least 12 months. Participant selection criteria will then vary by objective as detailed below.

Objective 1: Validation of non-interventional methods by comparing with trial results

An overview of each of the steps for participant selection for Objective 1 is provided in Figure 2.

Step 1

We will select all patients in the CPRD with COPD who are eligible for HES-linkage and during the period covered by the linkage would have met the following TORCH study **inclusion** criteria:

- a diagnosis of COPD
- age 40-80 years
- smoking history
- lung function (FEV_1 <60% predicted, FEV_1/FVC ratio<70%)

An eligible-for-inclusion date will then be assigned as the date that all of the above inclusion criteria were met for the individual. We will then exclude any individual who has any of the following TORCH study **exclusion** criteria prior to their eligible-for-inclusion date:

- a diagnosis of asthma (within the previous 5 years)
- a diagnosis for any (non-COPD) respiratory disorder
- a record of lung surgery
- a diagnosis of alpha-1 antitrypsin deficiency
- evidence of drug or alcohol abuse
- a record of having received long-term oxygen therapy
- diagnoses for conditions likely to interfere with the TORCH trial or cause death within 3 years
- current use of oral corticosteroid therapy (defined as continuous use for greater than 6 weeks, with courses of oral corticosteroidsteroids separated by a period of less than 7 days considered as continuous use)
- any exposure to FP/SAL within the previous 4 weeks

Finally, in-line with the TORCH trial approach, anyone who has an exacerbation requiring oral corticosteroid therapy or hospitalisation during the run-in period (the 2-week period following eligibility) will also be excluded.

Feasibility counts in the CPRD indicate there are ~13,000 patients meeting these criteria (Figure 2). Given the limited information on how asthma exclusions were applied in the TORCH study, we will perform a sensitivity analysis in which the asthma exclusion is a diagnosis within the previous 1 year, rather than 5 years as specified above.

For peer review only

Step 2

Next we will determine if/when these patients ever received FP/SAL. During any time between attaining TORCH eligibility and a subsequent prescription for FP/SAL, patients will be eligible for inclusion as an unexposed patient in Objective 1. There may be multiple time periods within a person's record where eligibility as an unexposed patient is met (Figure 3). Feasibility work shows that between 1st January 2004 and 1st January 2017 there were ~11,000 TORCH eligible patients in CPRD who did not receive FP/SAL at the time they attained TORCH eligibility and therefore have at least one time period that means they are eligible for inclusion as an Objective 1 unexposed participant (Figure 2). Individuals in CPRD who have more than one unexposed eligibility period within their record (Figure 3) will be able to contribute more than once to the pool of unexposed participants (with the covariates and person-time contributed unique to the specific unexposed eligibility period).

Step 3

Having obtained individual level patient data for TORCH participants from clinicalstudydatarequest.com, we will then match each TORCH participant (n=6,112) 1:1 with the closest available unexposed patient record in the CPRD pool of FPSAL untreated patients obtained in Step 2. Matching will be based on the following TORCH baseline characteristics:

- age
- sex
- body mass index
- previous treatment with:
    - inhaled corticosteroids
    - long acting beta-agonists
- history of COPD exacerbations
- history of cardiovascular disease
- lung function

Where an individual from CPRD has multiple unexposed "eligibility periods" that can be matched to a TORCH participant, the CPRD characteristics that will be matched on will be those from the beginning of the specific eligibility period.

Some of the TORCH inclusion criteria will not be fully assessable using CPRD data (e.g. we will be able to assess whether patients are smokers but will not always know their pack year history). Hence the inclusion/exclusion criteria are analogous with TORCH criteria but we acknowledge they are not identical. Identification of criteria will be done based on algorithms already determined and by the identification of clinical codes in the CPRD. For those individuals that have contributed multiple unexposed records to the pool of CPRD unexposed participants (Figure 3), after one of their unexposed records has been matched to a TORCH participant we will remove all of their other unexposed records (meaning that an individual can only appear once in the final TORCH-matched

For peer review only - http://bmjopen.bmj.com/site/about/guidelines.xhtml

unexposed cohort). We anticipate matching all or the majority of TORCH participants with a CPRD patient, giving us a pool of TORCH-analogous untreated patients within CPRD, with similar baseline characteristics as TORCH participants at the point of randomisation (n~6,000, Figure 2).

Step 4

Following this, we will select all patients in CPRD meeting the TORCH eligibility criteria specified above, and who also received treatment with FP/SAL (either on the date of eligibility or at a later date). From feasibility work, we anticipate ~12,000 eligible FP/SAL treated patients (Figure 2), some of whom may have multiple time-periods of treated patient eligibility and therefore could contribute more than once to the initial pool of treated patients (Figure 3). In contrast to the unexposed TORCH-eligible cohort, our initial approach will not involve matching participants of the exposed TORCH-eligible cohort with participants from the TORCH trial, as this would negatively impact the ability to calculate propensity scores for receiving FP/SAL in Step 5. Note that there will be overlap between those selected as untreated (in Step 2) and as treated (in Step 4). Approximately 90% of the Step 4 treated patients will also have been eligible as Step 2 untreated patients, as they will have had periods where they were not treated with FP/SAL and met the Step 2 untreated eligibility criteria in addition to separate periods where they were treated with FP/SAL and met the Step 4 treated eligibility criteria (Figure 3). If a person is included in both the untreated Step 2 and treated Step 4 cohorts, they will be contributing different periods of their person-time to each cohort (pre-FP/SAL treatment for Step 2 vs post-FP/SAL treatment for Step 4), and this will be handled in the analysis by assigning different index dates for Step 2 compared to Step 4.

Step 5

We will combine the CPRD groups obtained in Steps 3 and 4 (n~18,000, see note in Step 4 relating to 90% overlap) and using their baseline characteristics will calculate propensity scores for receiving FP/SAL. The propensity score calculation will be based upon a wide range of covariates (see statistical analysis section for full details). Where a participant is contributing more than one treated time period record to the pool of exposed records (as described in Step 4), baseline characteristics will be updated at the beginning of each treatment-eligible period. Multiple eligible treatment periods from a single person are then included in the propensity score model as if they came from different individuals. The variables selected for the score will then become the basis for propensity score modelling in Objectives 2 and 3.

Step 6

Each untreated patient derived in Step 3 (n~6,000) will be matched 1:1 with the FP/SAL treated patient record from Step 4 with the closest propensity score (~12,000) giving us an analysis population for Objective 1 of ~12,000 patients – double the size of TORCH (Figure 2). For those individuals that have contributed multiple exposed records to the pool of CPRD exposed participants (Figure 3), after one of their exposed records has been propensity-score matched to an unexposed

For peer review only

participant, we will remove all other exposed records for that individual from the remaining pool of CPRD exposed participants. This will mean that an individual can only appear once as an exposed participant in the final propensity-score matched cohort. The index date will be the start of follow up.

We will also apply an alternative additional approach for Objective 1, where instead of generating and using propensity scores to obtain a final analysis population at Steps 5 and 6, we will match records from our exposed TORCH-eligible cohort with participants from the TORCH trial to create a TORCH-analogous exposed patient cohort. This will then be combined with the TORCH-analogous unexposed patient cohort to create a final analysis population (with multivariable regression techniques used to account for confounding instead of propensity scores).

For peer review only

Figure 2: Flowchart illustrating the planned selection of CPRD participants for Objective 1 of the COPD real-world medicines effects study

[FIGURE 2 HERE]

For peer review only

Note in relation to Step 5 (n~18,000) compared to Step 1 (n~13,000): Approximately 90% of the treated patients will also have been eligible as untreated patients, as they did not receive FP/SAL on their TORCH-eligibility date. This means that they will have at least one period of time during which they are untreated-eligible but then did subsequently go-on to receive FP/SAL (meaning they have at least one period of time during which they are treated-eligible). If a person is included as both a treated and untreated participant, they will be contributing different periods of their person-time to each cohort (pre-FP/SAL treatment for the untreated versus post-FP/SAL treatment for treated), and this is handled in the analysis by assigning different index dates

Figure 3: Example timeline for a person in CPRD who is eligible for participation in Objective 1, illustrating multiple periods of unexposed/exposed (FL/SAL untreated/treated) eligibility.

[FIGURE 3 HERE]

1. 1st date of study eligibility: person meets TORCH eligibility as detailed in Objective 1, Step 1 on this date i.e. has a diagnosis of COPD, is between 0-80 years, FEV1<60% predicted and FEV1/FVC ratio <70%, smoking history, no asthma history, no lung surgery history, no long-term O_2 therapy, no alpha-1 antitrypsin deficiency, no drug/alcohol abuse, no exposure to FP/SAL within the previous 4 weeks.
2. Eligible to enter study as an untreated participant: patient can be selected as an untreated participant on any date within this period (as detailed in Step 2).
3. Eligible to enter study as a treated participant: FP/SAL treatment starts, patient is able to be selected as a treated study participant on the date that FP/SAL treatment starts (as detailed in Step 4).
4. Eligible to enter study as an untreated participant: patient stops treatment, but is not immediately eligible for selection again as an untreated study participant. After 4 weeks of no FP/SAL treatment however, they meet the TORCH eligibility ciriteria, and may be selected at any date during the remaining one untreated week as an untreated patient. This is the second untreated period that this person can contribute to the total pool of untreated period records that will be available for matching to the TORCH participants (as detailed in Steps 2 and 3).
5. Eligible to enter study as a treated participant: FP/SAL treatment (re)starts, patient can be selected as a treated study participant on the date that FP/SAL treatment (re)starts. This is the second treated period that this person can contribute to the total pool of treated period records that will be available for propensity score matching to the untreated participants (as detailed in Steps 4 to 6).

Objective 2: Measurement of COPD treatment effects in patients excluded from trials

We will include patients with a valid COPD diagnosis in the CPRD[8] meeting these additional criteria: (1) age >80 years OR (2) history of lung surgery OR (3) history of long term oxygen therapy OR (4) evidence of drug/alcohol abuse OR (4) an asthma diagnosis at any time prior to inclusion OR (5) substantial comorbidity. In relation to substantial comorbidity, TORCH required people to be excluded from the trial if they had serious uncontrolled disease with a likelihood of causing death within 3 years. It is likely this criterion affected participant selection and led to a lower overall rate of death than originally anticipated, although we recognise this criterion is subjective. During Objective 1 we will be able to select groups of people who were generally not included despite being eligible, most likely because of this subjective exclusion criterion. We anticipate this will be people with substantial comorbidity e.g. serious vascular disease. Status for such diseases is readily identified in both the CPRD data and in the TORCH baseline data. We will only be able to specify this criterion in detail after we have completed Objective 1.

Participants for Objective 2 will be selected in a similar fashion to Objective 1, with the additional eligibility criteria mentioned above applied (i.e. a modified Step 1). As for Objective 1, each participant will be allowed to have multiple FP/SAL exposed and unexposed eligibility periods in their record, as described in Figure 3. In contrast to Objective 1, there will be no matching of unexposed

For peer review only - http://bmjopen.bmj.com/site/about/guidelines.xhtml

patients to TORCH patients, as we do not require a TORCH-analagous cohort for this analysis (i.e. no Step 3). All other selection steps will be as applied for Objective 1, including the use of propensity score matching in order to obtain comparable unexposed and exposed groups for analysis.

Objective 3: Determination of treatment effects in an under-studied disease stage

We will include patients with a valid COPD diagnosis in the CPRD[8] meeting these additional criteria: (1) >60% predicted FEV1 (or >50% plus MRC breathlessness scale 1 or 2, or >50% plus COPD Assessment Test (CAT) score <10) AND (2) no exacerbations in the year post COPD diagnosis. We will also perform a sensitivity analysis where we allow the group of people with FEV1 >60% predicted who had a maximum of one exacerbation within 1 year post COPD diagnosis to be included. As for Objective 2, the selection steps will be similar to Objective 1, with modified criteria for step 1 and the removal of the TORCH-matching step (step 3).

Exposures, outcomes and co-variates

Exposures

For all objectives, exposures will be determined using CPRD prescribing records and code lists for COPD treatments (codelists provided in supplementary materials).

For Objective 1, incident use of FP/SAL (tradename Seretide) is the primary exposure of interest and will be compared with no treatment. We will limit included patients to those receiving Seretide 500/50, the dose used in TORCH. This information is recorded for all prescriptions of Seretide and this dose is the only currently approved dose for COPD in the UK (though we recognise some prescribing may not follow the licensed indication).

As a secondary analysis in Objective 1 other treatments for COPD will also be compared with no treatment in this objective. Namely:

a) long-acting beta agonist (LABA)
b) long-acting muscarinic antagonist (LAMA)
c) LABA + LAMA
d) LABA + inhaled corticosteroid (ICS) other than FP/SAL
f) LABA + LAMA + ICS

For Objectives 2 and 3 we will again use recorded prescribing information to determine the dose recieved. We will be reliant mostly on the strength of each individual drug which is recorded automatically against each product and does not require GPs to enter this data, ensuring completeness. We will then be able to stratify analyses based on the dose prescribed. Specific exposures for Objectives 2 and 3 are as follows:

a) No treatment
b) LABA

For peer review only - http://bmjopen.bmj.com/site/about/guidelines.xhtml

c) LAMA
d) LABA + LAMA
e) LABA + ICS
f) LABA + LAMA + ICS

Outcomes

Outcomes to be measured are as follows:

1. COPD exacerbation*:* to be defined using a CPRD-Hospital Episodes Statistics (HES) algorithm developed previously by authors of this study protocol[10]
2. All cause mortality: as recorded in ONS mortality statistics (data that is linked to CPRD data)
3. Pneumonia: as defined using a CPRD-HES algorithm published previously by authors of this study protocol[11]
4. Time to COPD treatment change: determined by prescribing records indicating the start of a new, additional COPD treatment

Covariates

Covariates to be considered for inclusion in the propensity score include the following (all obtained from CPRD data):

- Lung function (FEV1, FEV1/FVC)
- Age
- Gender
- Body mass index
- Alcohol consumption
- Vascular disease (broken into individual components e.g. hypertension, heart failure, atherosclerotic disease)
- Use of prescribed aspirin and statins
- Prior treatment with other COPD medication
- Type 2 diabetes
- History of cancer
- Renal disease
- Healthcare utilisation intensity (number of prior visits, hospitalisations, number of distinct medications used, number of procedures)

Sample size

Objective 1

Assuming a baseline conservative exacerbation rate of 0.5 per patient per year,[10] we would only require a sample of 408 patients per treatment group to detect a reduction in annual exacerbation rate to 0.4 per year, with 80% power and 5% significance. The estimated sample size is ~12,000 which will provide ample power for the main outcomes of interest, but also allow stratification by patient characteristics to determine stratified results, and will also be ample for the secondary analyses where we will use 99% confidence intervals. For example, to detect a reduction from 0.5 to

For peer review only

0.4 exacerbations per year with 80% power and 1% significance we would need ~600 people in each treatment group.

Objectives 2 and 3

We are also confident that we will have sufficient numbers to allow well powered analyses for Objectives 2 and 3. For example a feasibility count looking at the number of people over the age of 80 eligible for inclusion in Objective 2 estimated that there would be >2,000 people in each exposure group.

Statistical analysis

Propensity score for addressing confounding

The propensity score will be constructed using the principle that predictors of the exposure and outcome, or outcome only (mortality) should be included. We will consider a wide range of factors for inclusion, such as: age, sex, body mass index, alcohol consumption, and a wide range of comorbidities (e.g. type 2 diabetes, coronary heart disease, cerebrovascular disease, peripheral vascular disease, heart failure, hypertension, renal disease, cancer). We will further adjust for healthcare utilisation intensity (number of prior visits, hospitalisations, number of distinct medications used, number of procedures, etc.) as these are generic correlates of disease state and the likelihood of recording completeness. We have substantial prior experience of building propensity models,[13–15]and our study team includes the lead of an MRC funded project to determine optimal propensity score methods for use with missing data (EW).[16]

For Objective 1, every untreated patient will be PS matched 1:1 with an FP/SAL treated patient that has an FP/SAL treated period wih the closest propensity score, resulting in a TORCH analogous cohort of FP/SAL treated and untreated CPRD patients (n~12,000).

For the additional alternative approach to Objective 1 relying on matching of both unexposed and exposed patients to the TORCH trial patients (described in Selection of participants – Objective 1 – Step 6), we will use multivariable regression techniques to address confounding, considering a similar wide range of covariates for adjustment.

The propensity score model obtained in Objective 1 will be the basis for propensity score modelling in Objectives 2 and 3, but additional variables will also be considered given the different nature of the patient populations being studied in these Objectives.

Methods of analysis

Comparisons will be made according to FP/SAL status for rate of COPD exacerbation, pneumonia and mortality over 3 years. All analyses will be performed according to the intention-to-treat principle (as was done in the TORCH study), meaning that if a participant enters the study as either an exposed or unexposed participant, they will remain assigned to that exposure category for the entire duration of

For peer review only

their follow-up (irrespective as to whether their true exposure status changes). For exacerbations, a negative binomial model will be used, accounting for variability between patients in the number and frequency of exacerbations, with the number of exacerbations as the outcome and the log of treated time as an offset variable. Time to mortality and first pneumonia will be analysed using Cox proportional hazards regression. This mirrors TORCH endpoints of major benefit and harm. We anticipate the propensity matching process will allow us to assemble treated and untreated groups that are very similar with respect to baseline characteristics except FP/SAL treatment status. However, this will be tested by assessing standardised differences for each baseline variable. If substantial differences are noted for important variables, it may be necessary to further adjust the statistical models. This could also include examining the effect of using greedy vs optimum matching approaches in order to obtain the closest propensity score match, and/or matching at a ratio other than 1:1.[17]

Validation of results against TORCH

We will validate our findings against TORCH as part of Objective 1 by determining whether results of the CPRD analysis are compatible with the TORCH rate ratio for exacerbations (0.75; 95% CI 0.69-0.81). This outcome has been selected as it is an outcome of key significance for people with COPD[4] and the result in TORCH shows a clear benefit with 95% confidence intervals below 1. We have set two criteria that must be met for us to conclude results are consistent. First the effect size must be clinically comparable with TORCH findings; the rate ratio for exacerbations in CPRD must be between 0.65 and 0.9. This range is deliberately not symmetrical around the TORCH estimate of 0.75 as we would anticipate the treatment effect in routine clinical care may be weaker than that seen in the optimised setting of a randomised trial. We recognise this rule could be met with a poorly powered, inconclusive result, so a second criterion is that the 95% confidence interval for the rate ratio must exclude 1.

Handling measurement of adherence to medication

Adherence to issued prescribing in general practice is likely to vary according to the treatment issued e.g. short course antibiotic treatment is notoriously not well adhered to, whereas long term life-saving treatment such as antiretroviral medication is more likely to be taken as prescribed. Whilst we do not have figures for adherence for COPD medication in UK general practice, we are able to estimate the proportion of time covered by prescribing as a proxy for adherence and will account for this in our analyses. Moreover, our intention is to estimate the effect of prescribing at the population level, and to some extent, the clinical effects we will measure are in part due to pharmacological effects, and in part the way the treatment is taken which includes adherence. Also of note, prescribing for COPD in the UK is predominantly through GPs and so we will not be missing prescribing information from other potential sources of treatment.

For peer review only

The data analysis for adherence will necessarily be a significant element of the work to be done for this study. However, we have reviewed the records for a random sample of 30 people with COPD starting treatment with FP/SAL to look at adherence patterns over the course of a year. Of the 30 patients, 20 (67%) were still receiving Seretide (FP/SAL) one year after starting treatment. Of the 20 who received Seretide for a full year, 15 (75%) received sufficient prescriptions to suggest at least 50% adherence over the year, and 8 (40%) had sufficient prescriptions to suggest 80% adherence or higher. As expected, this suggests two things: Firstly adherence is likely to be poorer in routine clinical care than in the trial population; in TORCH 80% of participants were estimated to have adherence at 80% or higher. Secondly there is a wide range of adherence in routine care. This will allow us to estimate both the population level effects of treatment as actually used in routine care, but also to estimate the treatment effect in patients with more similar levels of adherence to TORCH participants. Whilst we acknowledge that prescribing can only be a proxy for used medication, we believe it is not an unreasonable assumption that the amount of medication prescribed is correlated with the amount consumed. In the event that Objective 1 detects a null or poorer treatment effect than anticipated (rate ratio > 0.9), we will conduct a sensitivity analysis restricted to people estimated to be covered by FP/SAL treatment for 80% of their follow up.

Misclassification of (1) drug exposure periods and (2) outcome status

It is possible that an individual may still be exposed to FP/SAL for some time after a prescription has finished, for example if they have medication at home that they haven't used from a previous prescription. This would mean that people may become eligible for inclusion in the unexposed group while they are actually still exposed. If our result differs from the TORCH results (e.g. a rate ratio <0.65 or >0.9), we will conduct a sensitivity analysis in which we include an additional (grace) exposed period equivalent to the length of a single prescription at the end of each actual exposed period, and only classify individuals as eligible for inclusion as unexposed at the end of this additional period.

Our results could also be impacted by misclassification of outcome, given the routine nature of the data. Our initial approach for detection of COPD exacerbations is to use a validated case definition from previous work that maximises positive predictive value while maintaining a relatively high sensitivity.[12] If our result differs from the TORCH results, we will consider performing a sensitivity analysis in which we assess the impact of applying alternative case definitions for COPD exacerbations (see supplementary materials for an overview of articles relating to the case definitions we plan to utilise, including any validity measurements provided).

Missing data

CPRD data are shown to be almost complete for drug prescribing and mortality (partly through ONS linkage). Smoking history tends to be very well recorded for people with COPD and missingness is

likely to be minimal.[10] Information on important comorbidity is also well recorded in CPRD. We will conduct both complete record analyses and use multiple imputation where appropriate assumptions hold, applying findings from methodological work led by one of the study team (EW) into the use of multiple imputation in propensity score modelling.[16]

Ethics and dissemination

Approval by ethics and scientific comittees

Ethical approval for this study has been obtained from the London School of Hygiene & Tropical Medicine Ethics Committee (Ref: 11997).

An application for scientific approval related to use of the CPRD data has been made to the Independent Scientific Advisory Committee (ISAC) of the Medicines and Healthcare Products Regulatory Agency. CPRD data are already approved via a National Research Ethics Committee for purely non-interventional research of this type.

An application for use of the TORCH trial individual patient data was made to the clinicalstudydatarequest.com site, which is checked by the Wellcome Trust and relevant sponsors to make sure information is complete and that the sponsor's requirements for informed consent have been met. The application is then sent to an independent review panel that consider the scientific rationale, objectives, publication plan, conflicts of interest and qualifications and experience of the research team before making a decision on providing access to the data. We recently obtained approval of all aspects of this application.

Dissemination plans

Dissemination of findings will be via a combination of channels. The work will be published in high ranking peer reviewed journals and we anticipate 3 publications to arise directly from the planned work. Findings will also be presented at relevant scientific conferences such as the British Thoracic Society Conference and the European Respiratory Society International Congress. We will also engage with patients already identified from a clinic run by one of the authors of this protocol (JQ) and from Breathe Easy Groups and with relevant charities such as the British Lung Foundation to determine the most relevant ways to disseminate results directly to patients in an accessible manner, and to help our understanding of the likely impact of results to specific groups of patients. We will communicate directly with NICE to ensure they are kept informed of results that are of direct relevance to the guidance they have issued on COPD, and with the Medicines and Healthcare Products Regulatory Agency if it appears that findings may impact the risk/benefit profile of COPD treatments.

For peer review only

References

1. Comission H. Cleaning the air: a national study of chronic pulmonary disease. 2006.

2. Calverley PMA, Anderson JA, Celli B, Ferguson GT, Jenkins C, Jones PW, et al. Salmeterol and Fluticasone Propionate and Survival in Chronic Obstructive Pulmonary Disease. N Engl J Med. 2007 Feb 22;356(8):775–89.

3. Tashkin DP, Celli B, Senn S, Burkhart D, Kesten S, Menjoge S, et al. A 4-Year Trial of Tiotropium in Chronic Obstructive Pulmonary Disease. N Engl J Med. 2008 Oct 9;359(15):1543–54.

4. National Institute for Health and Care Excellence (NICE). Chronic obstructive pulmonary disease in over 16s: diagnosis and management | Guidance and guidelines | NICE. 2010;

5. Ferguson GT, Anzueto A, Fei R, Emmett A, Knobil K, Kalberg C, et al. Maintenance pharmacotherapy of mild and moderate COPD: what is the evidence? Respir Med. 2011 Sep;105(9):1268–74.

6. EU. REGULATION (EU) No 1235/2010 OF THE EUROPEAN PARLIAMENT AND OF THE COUNCIL. 2010.

7. Spring S. Guidance for Industry Postmarketing Studies and Clinical Trials — Implementation of Section 505(o)(3) of the Federal Food, Drug, and Cosmetic Act.

8. Herrett E, Gallagher AM, Bhaskaran K, Forbes H, Mathur R, Staa T van, et al. Data Resource Profile: Clinical Practice Research Datalink (CPRD). Int J Epidemiol. 2015 Jun;44(3):827–36.

9. Herrett E, Thomas SL, Schoonen WM, Smeeth L, Hall AJ. Validation and validity of diagnoses in the General Practice Research Database: a systematic review. Br J Clin Pharmacol. 2010 Jan;69(1):4–14.

10. Quint JK, Müllerova H, DiSantostefano RL, Forbes H, Eaton S, Hurst JR, et al. Validation of chronic obstructive pulmonary disease recording in the Clinical Practice Research Datalink (CPRD-GOLD). BMJ Open. 2014;4(7):e005540.

11. Millett ERC, Quint JK, Smeeth L, Daniel RM, Thomas SL. Incidence of Community-Acquired Lower Respiratory Tract Infections and Pneumonia among Older Adults in the United Kingdom: A Population-Based Study. Heimesaat MM, editor. PLoS One. 2013 Sep 11;8(9):e75131.

12. Rothnie KJ, Müllerová H, Hurst JR, Smeeth L, Davis K, Thomas SL, et al. Validation of the Recording of Acute Exacerbations of COPD in UK Primary Care Electronic Healthcare Records. Chotirmall SH, editor. PLoS One. 2016 Mar 9;11(3):e0151357.

For peer review only

13. Douglas I, Evans S, Smeeth L. Effect of statin treatment on short term mortality after pneumonia episode: cohort study. BMJ. 2011 Apr 6;342:d1642.

14. Williamson E, Morley R, Lucas A, Carpenter J. Propensity scores: From naïve enthusiasm to intuitive understanding. Stat Methods Med Res. 2012 Jun 24;21(3):273–93.

15. Root AA, Wong AYS, Ghebremichael-Weldeselassie Y, Smeeth L, Bhaskaran K, Evans SJW, et al. Evaluation of the risk of cardiovascular events with clarithromycin using both propensity score and self-controlled study designs. Br J Clin Pharmacol. 2016 Aug;82(2):512–21.

16. Leyrat C, Seaman SR, White IR, Douglas I, Smeeth L, Kim J, et al. Propensity score analysis with partially observed covariates: How should multiple imputation be used? Stat Methods Med Res. 2017 Jun 2;96228021771303.

17. Austin PC. An Introduction to Propensity Score Methods for Reducing the Effects of Confounding in Observational Studies. Multivariate Behav Res. 2011 May;46(3):399–424.

For peer review only

Authors contributions

KW, EW, JC, LW, SS, LS, JQ, ID contributed to study question and design. KW wrote the first draft of the protocol manuscript (based upon original grant/scientific approval applications to NIHR and ISAC that KW, EW, JC, LW, SS, LS, JQ and ID all contributed to). KW, EW, JC, LW, SS, LS, JQ, ID contributed to further drafts and approved the final version.

Acknowledgements

No information for this section.

Data sharing statement

There are currently no unpublished data from this study, as it is a protocol.

All of the data sources described can be accessed by making formal applications to the owners of the data (i.e. CPRD/HES data for the routinely collected non-interventional data and clinicalstudyrequest.com for the trial data used for validation of non-interventional methods).

Figure legends

Figure 1: Overview of study objectives and sources of data for the COPD real-world medicines effects study (RCT=randomised controlled trial, CPRD= the UK Clinical Practice Research Datalink, FP/SAL=Fluticasone propionate + salmeterol)

Figure 2: Flowchart illustrating the planned selection of CPRD participants for Objective 1 of the COPD real-world medicines effects study

Figure 3: Example timeline for a person in CPRD who is eligible for participation in Objective 1, illustrating multiple periods of unexposed/exposed (FL/SAL untreated/treated) eligibility.

For peer review only

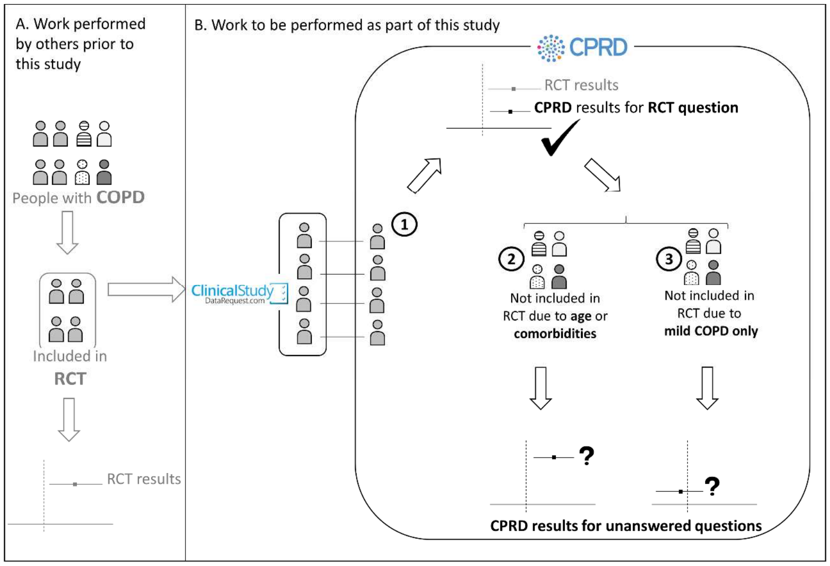

Figure 1: Overview of study objectives and sources of data for the COPD real-world medicines effects study (RCT=randomised controlled trial, CPRD= the UK Clinical Practice Research Datalink, FP/SAL=Fluticasone propionate + salmeterol)

338x190mm (300 x 300 DPI)

FIGURE 2

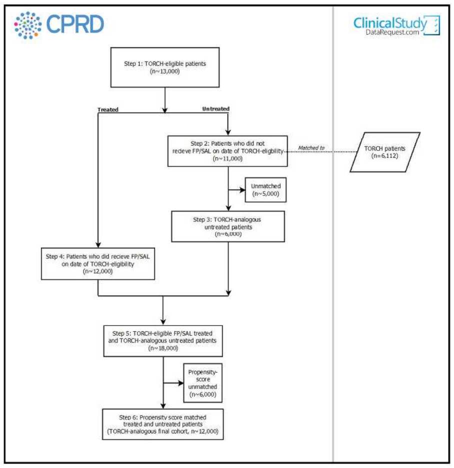

Figure 2: Flowchart illustrating the planned selection of CPRD participants for Objective 1 of the COPD real-world medicines effects study

338x190mm (300 x 300 DPI)

For peer review only

FIGURE 3

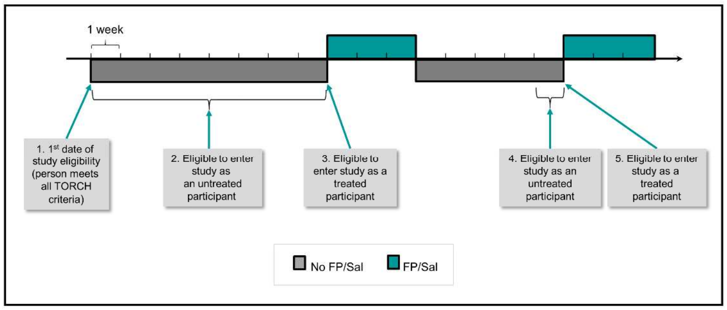

Figure 3: Example timeline for a person in CPRD who is eligible for participation in Objective 1, illustrating multiple periods of unexposed/exposed (FL/SAL untreated/treated) eligibility.

338x190mm (300 x 300 DPI)

review only

Overview of algorithms to be used for detecting COPD, COPD exacerbations and pneumonia

Condition	Paper (author, year)	Algorithm description[1]	Validity[2]	Other notes
COPD	Quint et al, 2014[10]	- CPRD[3] diagnostic (Read) code for COPD	PPV[4]: 87% (78 – 92)	1. Comparison with gold standard of respiratory physician review of information obtained by questionnaire from GPs<br>2. 8 algorithms presented in total, PPVs ranging from 12 to 89
COPD exacerbation	Rothnie et al, 2016[12]	- CPRD diagnostic (Read) code for LRTI or Acute Exacerbation COPD (AECOPD) **OR**<br>- A prescription of a COPD-specific antibiotic combined with OCS for 5-14 days **OR**<br>- A record (Read code) of two or more respiratory symptoms of AECOPD with a prescription of COPD-specific antibiotics and/or OCS on the same day	PPV: 86% (83 – 88)<br>Sensitivity: 63% (55 – 70)	1. Comparison with gold standard of respiratory physician review of information obtained by questionnaire from GPs<br>2. 15 algorithms presented in total, PPVs ranging from 61% – 100%, sensitivities ranging from 1.6% – 63%
Pneumonia	Millet et al, 2013[11]	- CPRD diagnostic (Read) codes and HES[5] diagnostic (ICD-10) codes for pneumonia (identified as a subset of an initial search for LRTI codes)<br>- Records in both database within the 28 days considered the same illness-episode	No validation performed	

Note 1: Main algorithm presented in article and to be applied initially in COPD medications real-world effects study (details on other algorithms presented in paper provided in the "Other notes" column where appropriate). **Note 2**: Validity=measure of validity presented in article:result obtained (95% CI). **Note 3**: CPRD=UK Clinical Practice Research Datalink **Note 4**: PPV=positive predictive value **Note 5:** HES=Hospital Episode Statistics

For peer review only

LAMA codes

Product code	BNF header	Drug substance	Drug product
61176	compound bronchodilator preparations	vilanterol trifenatate/umeclidinium bromide	anoro ellipta 55micrograms/dose / 22micrograms/dose dry powder inhaler (glaxosmithkline uk ltd)
61490	compound bronchodilator preparations		umeclidinium bromide 65micrograms/dose / vilanterol 22micrograms/dose dry powder inhaler
35014	antimuscarinic bronchodilators	tiotropium bromide monohydrate	tiotropium bromide 18microgram inhalation powder capsules with device
6474	antimuscarinics	glycopyrronium bromide	robinul 1mg tablet (idis world medicines)
50577	antimuscarinic bronchodilators	tiotropium bromide	spiriva 18microgram inhalation powder capsules with handihaler (de pharmaceuticals)
35011	antimuscarinic bronchodilators	tiotropium bromide monohydrate	tiotropium bromide 18microgram inhalation powder capsules
49227	antimuscarinic bronchodilators		aclidinium bromide 375micrograms/dose dry powder inhaler
50103	antimuscarinic bronchodilators		spiriva 18microgram inhalation powder capsules with handihaler (waymade healthcare plc)
7908	antimuscarinics	glycopyrronium bromide	robinul 2mg tablet (wyeth pharmaceuticals)
59638	antimuscarinic bronchodilators		spiriva 18microgram inhalation powder capsules with handihaler (sigma pharmaceuticals plc)
51967	antimuscarinic bronchodilators	tiotropium bromide	spiriva 18microgram inhalation powder capsules (mawdsley-brooks & company ltd)
53982	antimuscarinic bronchodilators		seebri breezhaler 44microgram inhalation powder capsules with device (novartis pharmaceuticals uk ltd)
6050	antimuscarinic bronchodilators	tiotropium bromide	spiriva 18 microgram capsule (boehringer ingelheim ltd)
7597	other ulcer healing drugs	glycopyrronium bromide	glycopyrronium bromide 2mg tablets
49228	antimuscarinic bronchodilators		eklira 322micrograms/dose genuair (almirall ltd)
59173	other ulcer healing drugs	glycopyrronium bromide	glycopyrronium bromide 200micrograms/5ml oral suspension

For peer review only

Product code	BNF header	Drug substance	Drug product
36864	antimuscarinic bronchodilators	tiotropium bromide	tiotropium bromide 2.5micrograms/dose solution for inhalation cartridge with device cfc free
55911	other ulcer healing drugs	glycopyrronium bromide	glycopyrronium bromide 500micrograms/5ml oral solution
34995	antimuscarinic bronchodilators	tiotropium bromide	spiriva 18microgram inhalation powder capsules with handihaler (boehringer ingelheim ltd)
35000	antimuscarinic bronchodilators	tiotropium bromide	spiriva 18microgram inhalation powder capsules (boehringer ingelheim ltd)
29138	other ulcer healing drugs	glycopyrronium bromide	glycopyrronium bromide 1mg/5ml oral solution
47269	other ulcer healing drugs	glycopyrronium bromide	glycopyrronium bromide 1mg/5ml oral suspension
54151	other ulcer healing drugs	glycopyrronium bromide	glycopyrronium bromide 600micrograms/5ml oral suspension
55795	other ulcer healing drugs	glycopyrronium bromide	glycopyrronium bromide 500micrograms/5ml oral suspension
38377	other ulcer healing drugs	glycopyrronium bromide	glycopyrronium bromide 2mg/5ml oral solution
7218	other ulcer healing drugs	glycopyrronium bromide	glycopyrronium bromide 1mg tablets
36869	antimuscarinic bronchodilators	tiotropium bromide	spiriva respimat 2.5micrograms/dose solution for inhalation cartridge with device (boehringer ingelheim ltd)
62109	antimuscarinic bronchodilators		umeclidinium bromide 65micrograms/dose dry powder inhaler
50292	antimuscarinic bronchodilators		spiriva 18microgram inhalation powder capsules (sigma pharmaceuticals plc)
55794	other ulcer healing drugs	glycopyrronium bromide	glycopyrronium bromide 5mg/5ml oral suspension
50047	other ulcer healing drugs	glycopyrronium bromide	glycopyrronium bromide 5mg/5ml oral solution
56262	other ulcer healing drugs	glycopyrronium bromide	glycopyrronium bromide 200micrograms/5ml oral solution
61879	antimuscarinic bronchodilators		incruse ellipta 55micrograms/dose dry powder inhaler (glaxosmithkline uk ltd)
53761	antimuscarinic bronchodilators		glycopyrronium bromide 55microgram inhalation powder capsules with device
746	antimuscarinic bronchodilators	tiotropium bromide	tiotropium 18 microgram capsule
38538	other ulcer healing drugs	glycopyrronium bromide	glycopyrronium bromide 2mg/5ml oral suspension
46214	antimuscarinics	glycopyrronium bromide	glycopyrronium bromide 5mg/5ml oral solution

For peer review only

Product code	BNF header	Drug substance	Drug product
61582	antimuscarinic bronchodilators	tiotropium bromide	spiriva respimat 2.5micrograms/dose solution for inhalation cartridge with device (waymade healthcare plc)

LABA codes

Product code	BNF header	Drug substance	Drug product
45610	selective beta 2 agonists	indacaterol maleate	indacaterol 300microgram inhalation powder capsules with device
7270	selective beta 2 agonists	salmeterol xinafoate	salmeterol 25micrograms/dose inhaler cfc free
10968	selective beta 2 agonists	formoterol fumarate dihydrate	foradil 12microgram inhalation powder capsules with device (novartis pharmaceuticals uk ltd)
549	unknown	salmeterol xinafoate	serevent 25micrograms/dose inhaler (glaxosmithkline uk ltd)
7133	selective beta 2 agonists	formoterol fumarate dihydrate	formoterol 12micrograms/dose dry powder inhaler
54742	selective beta 2 agonists	salmeterol xinafoate	salmeterol 25micrograms/dose inhaler cfc free (a a h pharmaceuticals ltd)
719	selective beta 2 agonists	salmeterol xinafoate	salmeterol 50micrograms/dose dry powder inhaler
57694	selective beta 2 agonists	salmeterol xinafoate	vertine 25micrograms/dose inhaler cfc free (teva uk ltd)
26829	selective beta 2 agonists	tulobuterol	brelomax 2mg tablet (abbott laboratories ltd)
19799	selective beta 2 agonists	tulobuterol	tulobuterol 2mg
56482	selective beta 2 agonists	formoterol fumarate dihydrate	oxis 12 turbohaler (waymade healthcare plc)
25784	selective beta 2 agonists	formoterol fumarate dihydrate	atimos modulite 12micrograms/dose inhaler (chiesi ltd)
47638	selective beta 2 agonists	salmeterol xinafoate	neovent 25micrograms/dose inhaler cfc free (kent pharmaceuticals ltd)
10672	peripheral vasodilators and related drugs	moxisylyte hydrochloride	opilon 40mg tablet (concord pharmaceuticals ltd)
50051	selective beta 2 agonists	salmeterol xinafoate	serevent 25micrograms/dose evohaler (waymade healthcare plc)
35725	selective beta 2 agonists	formoterol fumarate dihydrate	formoterol easyhaler 12micrograms/dose dry powder inhaler (orion pharma (uk) ltd)

For peer review only

Product code	BNF header	Drug substance	Drug product
2224	selective beta 2 agonists	salmeterol xinafoate	serevent 50micrograms/dose accuhaler (glaxosmithkline uk ltd)
43893	selective beta 2 agonists	indacaterol maleate	onbrez breezhaler 150microgram inhalation powder capsules with device (novartis pharmaceuticals uk ltd)
6526	selective beta 2 agonists	formoterol fumarate dihydrate	formoterol 12microgram inhalation powder capsules with device
43738	selective beta 2 agonists	indacaterol maleate	indacaterol 150microgram inhalation powder capsules with device
35825	selective beta 2 agonists	salmeterol xinafoate	serevent 50microgram disks (glaxosmithkline uk ltd)
43764	peripheral vasodilators and related drugs	moxisylyte hydrochloride	opilon 40mg tablets (archimedes pharma uk ltd)
56478	selective beta 2 agonists	salmeterol xinafoate	serevent 50micrograms/dose accuhaler (de pharmaceuticals)
42103	selective beta 2 agonists	tulobuterol	tulobuterol 1mg/5ml sugar free syrup
57544	selective beta 2 agonists	salmeterol xinafoate	serevent 50micrograms/dose accuhaler (waymade healthcare plc)
3297	selective beta 2 agonists	salmeterol xinafoate	salmeterol 50micrograms disc
57558	selective beta 2 agonists	formoterol fumarate dihydrate	oxis 6 turbohaler (lexon (uk) ltd)
9711	selective beta 2 agonists	formoterol fumarate dihydrate	formoterol 6micrograms/dose dry powder inhaler
35542	selective beta 2 agonists	salmeterol xinafoate	salmeterol 50microgram inhalation powder blisters with device
465	unknown	salmeterol xinafoate	salmeterol 25micrograms/dose inhaler
44064	selective beta 2 agonists	indacaterol maleate	onbrez breezhaler 300microgram inhalation powder capsules with device (novartis pharmaceuticals uk ltd)
35165	selective beta 2 agonists	salmeterol xinafoate	serevent 50microgram disks with diskhaler (glaxosmithkline uk ltd)
14306	selective beta 2 agonists	formoterol fumarate dihydrate	formoterol 12micrograms/dose inhaler cfc free
1974	selective beta 2 agonists	formoterol fumarate dihydrate	oxis 12 turbohaler (astrazeneca uk ltd)
7268	selective beta 2 agonists	salmeterol xinafoate	serevent 25micrograms/dose evohaler (glaxosmithkline uk ltd)
8365	peripheral vasodilators and related drugs	moxisylyte hydrochloride	moxisylyte 40mg tablets
35503	selective beta 2 agonists	salmeterol xinafoate	salmeterol 50microgram inhalation powder blisters
1975	selective beta 2 agonists	formoterol fumarate dihydrate	oxis 6 turbohaler (astrazeneca uk ltd)
22663	selective beta 2 agonists	tulobuterol	respacal 2mg tablet (ucb pharma ltd)

Product code	BNF header	Drug substance	Drug product
910	selective beta 2 agonists	salmeterol xinafoate	serevent diskhaler 50microgram inhalation powder (glaxo wellcome uk ltd)

For peer review only

ICS codes

Product code	BNF header	Drug substance	Drug product
54399	corticosteroids (for respiratory conditions)	beclometasone dipropionate	qvar 100 autohaler (sigma pharmaceuticals plc)
959	unknown	budesonide	budesonide 50micrograms/dose inhaler
2951	corticosteroids (for respiratory conditions)	fluticasone propionate	fluticasone 250microgram/actuation pressurised inhalation
50129	corticosteroids (for respiratory conditions)	beclometasone dipropionate	qvar 100micrograms/dose easi-breathe inhaler (de pharmaceuticals)
2229	corticosteroids (for respiratory conditions)	beclometasone dipropionate	becodisks 100microgram disc (allen & hanburys ltd)
3989	corticosteroids (for respiratory conditions)	fluticasone propionate	flixotide 100microgram disc (allen & hanburys ltd)
5551	corticosteroids (for respiratory conditions)	fluticasone propionate	flixotide 0.5mg/2ml nebules (glaxosmithkline uk ltd)
34794	corticosteroids (for respiratory conditions)	beclometasone dipropionate	beclometasone 200micrograms/dose inhaler (a a h pharmaceuticals ltd)
41269	corticosteroids (for respiratory conditions)	beclometasone dipropionate	beclometasone 400 cyclocaps (teva uk ltd)
29325	corticosteroids (for respiratory conditions)	beclometasone dipropionate	beclometasone 250micrograms/dose inhaler (generics (uk) ltd)
4499	corticosteroids (for respiratory conditions)	beclometasone dipropionate	aerobec 250microgram/actuation pressurised inhalation (meda pharmaceuticals ltd)
57589	corticosteroids (for respiratory conditions)	beclometasone dipropionate	becloforte 250micrograms/dose inhaler (dowelhurst ltd)
32874	corticosteroids (for respiratory conditions)	beclometasone dipropionate	beclometasone 50microgram/actuation inhalation powder (actavis uk ltd)
2159	corticosteroids (for respiratory conditions)	beclometasone dipropionate	aerobec 50 autohaler (meda pharmaceuticals ltd)
14590	corticosteroids (for respiratory conditions)	beclometasone dipropionate	asmabec 250microgram/actuation spacehaler (celltech pharma europe ltd)
1725	corticosteroids (for respiratory conditions)	beclometasone dipropionate	beclazone 50 easi-breathe inhaler (teva uk ltd)
908	corticosteroids (for respiratory conditions)	budesonide	pulmicort 400 turbohaler (astrazeneca uk ltd)
35288	corticosteroids (for respiratory conditions)	beclometasone dipropionate	beclometasone 400microgram inhalation powder blisters
49367	corticosteroids (for respiratory conditions)	beclometasone dipropionate	clenil modulite 50micrograms/dose inhaler (mawdsley-brooks & company ltd)
35107	corticosteroids (for respiratory conditions)	beclometasone dipropionate	beclometasone 400microgram inhalation powder blisters with device

For peer review only

Product code	BNF header	Drug substance	Drug product
1676	corticosteroids (for respiratory conditions)	fluticasone propionate	flixotide 125microgram/actuation inhalation powder (allen & hanburys ltd)
49772	corticosteroids (for respiratory conditions)	fluticasone propionate	fluticasone 250micrograms/dose evohaler (sigma pharmaceuticals plc)
48088	unknown	budesonide	budenofalk 9mg gastro-resistant granules sachets (dr. falk pharma uk ltd)
33258	corticosteroids (for respiratory conditions)	beclometasone dipropionate	beclometasone 250micrograms/dose inhaler (a a h pharmaceuticals ltd)
13037	corticosteroids (for respiratory conditions)	beclometasone dipropionate	pulvinal beclometasone dipropionate 200micrograms/dose dry powder inhaler (chiesi ltd)
19031	corticosteroids (for respiratory conditions)	beclometasone dipropionate	bdp 100microgram/actuation spacehaler (celltech pharma europe ltd)
16158	corticosteroids (for respiratory conditions)	beclometasone dipropionate	clenil modulite 50micrograms/dose inhaler (chiesi ltd)
2124	unknown		pulmicort refil 200 mcg inh
4759	corticosteroids (for respiratory conditions)	beclometasone dipropionate	beclometasone 100microgram inhalation powder capsules
3753	unknown		flixotide diskhaler-community pack 250 mcg
49711	corticosteroids (for respiratory conditions)	budesonide	pulmicort 200micrograms/dose inhaler (astrazeneca uk ltd)
15326	corticosteroids (for respiratory conditions)	beclometasone dipropionate	beclometasone 100micrograms/dose inhaler cfc free
2335	corticosteroids (for respiratory conditions)	beclometasone dipropionate	qvar 100 inhaler (teva uk ltd)
5580	corticosteroids (for respiratory conditions)	fluticasone propionate	flixotide accuhaler 50 50microgram/inhalation inhalation powder (allen & hanburys ltd)
51681	corticosteroids (for respiratory conditions)	beclometasone dipropionate	qvar 100 inhaler (sigma pharmaceuticals plc)
8433	corticosteroids (for respiratory conditions)	budesonide	budesonide 100micrograms/actuation inhaler
9577	corticosteroids (for respiratory conditions)	beclometasone dipropionate	asmabec 50 clickhaler (focus pharmaceuticals ltd)
2723	corticosteroids (for respiratory conditions)	fluticasone propionate	fluticasone 25micrograms/dose inhaler
23675	unknown		pulmicort l.s. refil
35638	corticosteroids (for respiratory conditions)	fluticasone propionate	fluticasone propionate 100microgram inhalation powder blisters with device
9571	corticosteroids (for respiratory conditions)	beclometasone dipropionate	beclometasone 250micrograms/actuation vortex inhaler
51234	corticosteroids (for respiratory conditions)	beclometasone dipropionate	qvar 100 inhaler (waymade healthcare plc)

For peer review only

Product code	BNF header	Drug substance	Drug product
60946	corticosteroids (in chronic bowel disorders)	budesonide	entocort cr 3mg capsules (waymade healthcare plc)
5223	corticosteroids (for respiratory conditions)	fluticasone propionate	fluticasone 50micrograms/dose inhaler cfc free
53057	corticosteroids (for respiratory conditions)	fluticasone propionate	flixotide 50micrograms/dose evohaler (lexon (uk) ltd)
13290	corticosteroids (for respiratory conditions)	beclometasone dipropionate	clenil modulite 100micrograms/dose inhaler (chiesi ltd)
42928	corticosteroids (for respiratory conditions)	fluticasone propionate	flixotide 100micrograms/dose accuhaler (glaxosmithkline uk ltd)
14736	corticosteroids (for respiratory conditions)	beclometasone dipropionate	pulvinal beclometasone dipropionate 400micrograms/dose dry powder inhaler (chiesi ltd)
52806	corticosteroids (for respiratory conditions)	beclometasone dipropionate	qvar 100 autohaler (lexon (uk) ltd)
57525	corticosteroids (for respiratory conditions)	fluticasone propionate	flixotide 250micrograms/dose accuhaler (stephar (u.k.) ltd)
33849	corticosteroids (for respiratory conditions)	beclometasone dipropionate	beclometasone 100microgram/actuation inhalation powder (neo laboratories ltd)
52732	corticosteroids (for respiratory conditions)	budesonide	pulmicort 0.5mg respules (necessity supplies ltd)
35611	corticosteroids (for respiratory conditions)	fluticasone propionate	flixotide 250microgram disks (glaxosmithkline uk ltd)
8111	corticosteroids (for respiratory conditions)	beclometasone dipropionate	becloforte vm 250microgram/actuation vm pack (allen & hanburys ltd)
39879	unknown	budesonide	budesonide 200micrograms/dose inhaler cfc free
1242	corticosteroids (for respiratory conditions)	beclometasone dipropionate	beclometasone 250micrograms/dose inhaler
26665	unknown		pulmicort complete
37447	corticosteroids (for respiratory conditions)	fluticasone propionate	fluticasone propionate 50microgram inhalation powder blisters
5718	corticosteroids (for respiratory conditions)	fluticasone propionate	flixotide 125micrograms/dose evohaler (glaxosmithkline uk ltd)
7653	corticosteroids (for respiratory conditions)	beclometasone dipropionate	beclometasone 400microgram inhalation powder capsules
28640	corticosteroids (for respiratory conditions)	beclometasone dipropionate	beclometasone 100microgram/actuation inhalation powder (actavis uk ltd)
4365	corticosteroids (for respiratory conditions)	beclometasone dipropionate	beclometasone 100micrograms disc
34739	corticosteroids (for respiratory conditions)	beclometasone dipropionate	beclometasone 50micrograms/dose inhaler (teva uk ltd)
8635	corticosteroids (for respiratory conditions)	fluticasone propionate	flixotide 50microgram disc (allen & hanburys ltd)

For peer review only

Product code	BNF header	Drug substance	Drug product
57579	corticosteroids (for respiratory conditions)	fluticasone propionate	flixotide 50micrograms/dose accuhaler (de pharmaceuticals)
4413	corticosteroids (for respiratory conditions)	beclometasone dipropionate	qvar 100 autohaler (teva uk ltd)
37203	corticosteroids (in chronic bowel disorders)	beclometasone dipropionate	beclometasone 5mg gastro-resistant modified-release tablets
4132	corticosteroids (for respiratory conditions)	fluticasone propionate	fluticasone 125microgram/actuation pressurised inhalation
3018	corticosteroids (for respiratory conditions)	beclometasone dipropionate	beclometasone 50micrograms/dose inhaler
9233	corticosteroids (for respiratory conditions)	beclometasone dipropionate	beclometasone 200microgram inhalation powder capsules
2160	corticosteroids (for respiratory conditions)	beclometasone dipropionate	beclometasone 50micrograms/dose breath actuated inhaler
2148	corticosteroids (for respiratory conditions)	beclometasone dipropionate	beclometasone 400microgram disc
34919	corticosteroids (for respiratory conditions)	beclometasone dipropionate	beclometasone 50micrograms/dose inhaler (a a h pharmaceuticals ltd)
2092	corticosteroids (for respiratory conditions)	budesonide	budesonide 200micrograms/dose dry powder inhaler
21005	corticosteroids (for respiratory conditions)	beclometasone dipropionate	beclometasone 250micrograms/dose inhaler cfc free
10090	corticosteroids (for respiratory conditions)	beclometasone dipropionate	beclometasone 50micrograms/actuation extrafine particle cfc free inhaler
1551	corticosteroids (for respiratory conditions)	beclometasone dipropionate	beclazone 250 inhaler (teva uk ltd)
39067	corticosteroids (in chronic bowel disorders)	beclometasone dipropionate	clipper 5mg gastro-resistant modified-release tablets (chiesi ltd)
51415	corticosteroids (for respiratory conditions)	beclometasone dipropionate	qvar 50 inhaler (mawdsley-brooks & company ltd)
35106	corticosteroids (for respiratory conditions)	beclometasone dipropionate	becodisks 100microgram with diskhaler (glaxosmithkline uk ltd)
35299	corticosteroids (for respiratory conditions)	beclometasone dipropionate	becodisks 400microgram (glaxosmithkline uk ltd)
54207	corticosteroids (for respiratory conditions)	beclometasone dipropionate	qvar 50 inhaler (de pharmaceuticals)
3927	corticosteroids (for respiratory conditions)	beclometasone dipropionate	filair 100 inhaler (meda pharmaceuticals ltd)
17670	corticosteroids (for respiratory conditions)	budesonide	easyhaler budesonide 100micrograms/dose dry powder inhaler (orion pharma (uk) ltd)
883	corticosteroids (for respiratory conditions)	beclometasone dipropionate	becodisks 200microgram disc (allen & hanburys ltd)
8450	unknown		flixotide diskhaler-community pack 50 mcg
3289	corticosteroids (for respiratory conditions)	fluticasone propionate	flixotide 25micrograms/dose inhaler (glaxosmithkline uk ltd)

For peer review only

Product code	BNF header	Drug substance	Drug product
53480	corticosteroids (for respiratory conditions)	beclometasone dipropionate	qvar 100 autohaler (stephar (u.k.) ltd)
39102	unknown	budesonide	budesonide 100micrograms/dose inhaler cfc free
20763	unknown		becloforte
35374	corticosteroids (for respiratory conditions)	fluticasone propionate	flixotide 500microgram disks (glaxosmithkline uk ltd)
39200	corticosteroids (for respiratory conditions)	beclometasone dipropionate	aerobec forte 250 autohaler (meda pharmaceuticals ltd)
9599	corticosteroids (for respiratory conditions)	beclometasone dipropionate	beclazone 50microgram/actuation inhalation powder (actavis uk ltd)
5804	corticosteroids (for respiratory conditions)	beclometasone dipropionate	beclometasone 250micrograms/dose dry powder inhaler
47943	corticosteroids (for respiratory conditions)	beclometasone dipropionate	beclazone easi-breathe (roi) 100microgram/actuation pressurised inhalation (ivax pharmaceuticals ireland)
5683	corticosteroids (for respiratory conditions)	fluticasone propionate	flixotide 250micrograms/dose evohaler (glaxosmithkline uk ltd)
48340	corticosteroids (for respiratory conditions)	beclometasone dipropionate	clenil modulite 100micrograms/dose inhaler (mawdsley-brooks & company ltd)
1885	corticosteroids (for respiratory conditions)	beclometasone dipropionate	beclazone 200 inhaler (teva uk ltd)
911	corticosteroids (for respiratory conditions)	fluticasone propionate	flixotide accuhaler 250 250microgram/inhalation inhalation powder (allen & hanburys ltd)
6095	corticosteroids (in chronic bowel disorders)	budesonide	budesonide 3mg gastro-resistant capsules
28073	corticosteroids (for respiratory conditions)	beclometasone dipropionate	beclometasone 250microgram/actuation pressurised inhalation (approved prescription services ltd)
4131	corticosteroids (for respiratory conditions)	fluticasone propionate	fluticasone 100microgram disc
43074	corticosteroids (for respiratory conditions)	fluticasone propionate	flixotide 500micrograms/dose accuhaler (glaxosmithkline uk ltd)
35905	corticosteroids (for respiratory conditions)	fluticasone propionate	fluticasone propionate 250microgram inhalation powder blisters
27583	unknown		pulmicort
17654	corticosteroids (for respiratory conditions)	beclometasone dipropionate	easyhaler beclometasone 200micrograms/dose dry powder inhaler (orion pharma (uk) ltd)
4545	corticosteroids (for respiratory conditions)	budesonide	pulmicort ls 50microgram refill canister (astrazeneca uk ltd)
1412	corticosteroids (for respiratory conditions)	fluticasone propionate	flixotide 250microgram/actuation inhalation powder (allen & hanburys ltd)

For peer review only

Product code	BNF header	Drug substance	Drug product
3363	corticosteroids (for respiratory conditions)	beclometasone dipropionate	becloforte 400microgram disks with diskhaler (glaxosmithkline uk ltd)
36462	corticosteroids (for respiratory conditions)	fluticasone propionate	fluticasone propionate 500microgram inhalation powder blisters
56471	corticosteroids (for respiratory conditions)	beclometasone dipropionate	becodisks 200microgram (mawdsley-brooks & company ltd)
14294	corticosteroids (for respiratory conditions)	beclometasone dipropionate	qvar 50micrograms/dose easi-breathe inhaler (teva uk ltd)
3570	corticosteroids (for respiratory conditions)	budesonide	budesonide 200micrograms/actuation refill canister
9164	corticosteroids (for respiratory conditions)	fluticasone propionate	fluticasone propionate 50micrograms/dose dry powder inhaler
27188	corticosteroids (for respiratory conditions)	budesonide	easyhaler budesonide 200micrograms/dose dry powder inhaler (orion pharma (uk) ltd)
39099	unknown	budesonide	pulmicort 100micrograms/dose inhaler cfc free (astrazeneca uk ltd)
4926	corticosteroids (for respiratory conditions)	fluticasone propionate	flixotide accuhaler 100 100microgram/inhalation inhalation powder (allen & hanburys ltd)
1642	corticosteroids (for respiratory conditions)	budesonide	budesonide 400micrograms/dose dry powder inhaler
34859	corticosteroids (for respiratory conditions)	beclometasone dipropionate	beclometasone 250microgram/actuation inhalation powder (neo laboratories ltd)
16054	corticosteroids (for respiratory conditions)	budesonide	budesonide 200micrograms/actuation breath actuated powder inhaler
960	corticosteroids (for respiratory conditions)	budesonide	pulmicort 100 turbohaler (astrazeneca uk ltd)
51480	corticosteroids (for respiratory conditions)	beclometasone dipropionate	qvar 100 autohaler (de pharmaceuticals)
7891	corticosteroids (for respiratory conditions)	fluticasone propionate	fluticasone 500microgram disc
56462	corticosteroids (for respiratory conditions)	beclometasone dipropionate	becodisks 400microgram (waymade healthcare plc)
7638	corticosteroids (for respiratory conditions)	fluticasone propionate	fluticasone 250microgram disc
15706	corticosteroids (for respiratory conditions)	beclometasone dipropionate	beclometasone 100 micrograms/actuation vortex inhaler
27915	unknown		fluticasone prop disk refill
2893	corticosteroids (for respiratory conditions)	beclometasone dipropionate	beclometasone 200micrograms disc
5885	corticosteroids (for respiratory conditions)	fluticasone propionate	fluticasone propionate 100micrograms/dose dry powder inhaler

For peer review only

Product code	BNF header	Drug substance	Drug product
3898	corticosteroids (in chronic bowel disorders)	budesonide	budesonide 3mg gastro-resistant modified-release capsules
3546	corticosteroids (for respiratory conditions)	beclometasone dipropionate	qvar 50 inhaler (teva uk ltd)
30210	corticosteroids (for respiratory conditions)	beclometasone dipropionate	beclometasone 250micrograms/dose inhaler (teva uk ltd)
1552	corticosteroids (for respiratory conditions)	beclometasone dipropionate	becloforte easi-breathe 250microgram/actuation pressurised inhalation (allen & hanburys ltd)
5822	corticosteroids (for respiratory conditions)	fluticasone propionate	fluticasone 250micrograms/dose inhaler cfc free
35430	corticosteroids (for respiratory conditions)	beclometasone dipropionate	becodisks 200microgram with diskhaler (glaxosmithkline uk ltd)
42994	corticosteroids (for respiratory conditions)	fluticasone propionate	flixotide 250micrograms/dose accuhaler (glaxosmithkline uk ltd)
36290	corticosteroids (for respiratory conditions)	fluticasone propionate	flixotide 50microgram disks with diskhaler (glaxosmithkline uk ltd)
24898	corticosteroids (for respiratory conditions)	beclometasone dipropionate	spacehaler bdp 100microgram/actuation spacehaler (celltech pharma europe ltd)
35700	corticosteroids (for respiratory conditions)	fluticasone propionate	fluticasone propionate 500microgram inhalation powder blisters with device
3188	unknown		pulmicort complete 50 mcg inh
7788	corticosteroids (for respiratory conditions)	budesonide	budesonide 100micrograms/dose dry powder inhaler
35293	corticosteroids (for respiratory conditions)	beclometasone dipropionate	beclometasone 200microgram inhalation powder blisters with device
8251	unknown		pulmicort refil 50 mg inh
1259	corticosteroids (for respiratory conditions)	beclometasone dipropionate	beclometasone 200micrograms/dose inhaler
16525	corticosteroids (in chronic bowel disorders)	budesonide	budenofalk 3mg gastro-resistant capsules (dr. falk pharma uk ltd)
1680	corticosteroids (for respiratory conditions)	budesonide	pulmicort ls 50micrograms/dose inhaler (astrazeneca uk ltd)
10254	corticosteroids (for respiratory conditions)	mometasone furoate	mometasone 400micrograms/dose dry powder inhaler
20812	unknown		pulmicort refill
14524	corticosteroids (for respiratory conditions)	beclometasone dipropionate	bdp 250microgram/actuation spacehaler (celltech pharma europe ltd)
2992	corticosteroids (for respiratory conditions)	beclometasone dipropionate	beclazone 50 inhaler (teva uk ltd)
7602	corticosteroids (for respiratory conditions)	fluticasone propionate	fluticasone 50microgram disc

For peer review only

Product code	BNF header	Drug substance	Drug product
16305	corticosteroids (for respiratory conditions)	fluticasone propionate	flixotide 2mg/2ml nebules (glaxosmithkline uk ltd)
14757	corticosteroids (for respiratory conditions)	beclometasone dipropionate	pulvinal beclometasone dipropionate 100micrograms/dose dry powder inhaler (chiesi ltd)
909	unknown	budesonide	budesonide 200micrograms/dose inhaler
1959	corticosteroids (for respiratory conditions)	budesonide	pulmicort 0.5mg respules (astrazeneca uk ltd)
1861	corticosteroids (for respiratory conditions)	beclometasone dipropionate	aerobec 100 autohaler (meda pharmaceuticals ltd)
16018	corticosteroids (for respiratory conditions)	mometasone furoate	mometasone 200micrograms/dose dry powder inhaler
35631	corticosteroids (for respiratory conditions)	budesonide	budelin novolizer 200micrograms/dose inhalation powder (meda pharmaceuticals ltd)
60937	corticosteroids (for respiratory conditions)	budesonide	pulmicort 200 turbohaler (dowelhurst ltd)
50037	corticosteroids (for respiratory conditions)	budesonide	pulmicort 0.5mg respules (waymade healthcare plc)
19389	corticosteroids (for respiratory conditions)	beclometasone dipropionate	asmabec 50microgram/actuation spacehaler (celltech pharma europe ltd)
2282	corticosteroids (for respiratory conditions)	fluticasone propionate	fluticasone propionate 500micrograms/dose dry powder inhaler
18848	corticosteroids (for respiratory conditions)	beclometasone dipropionate	qvar 100micrograms/dose easi-breathe inhaler (teva uk ltd)
2440	corticosteroids (for respiratory conditions)	fluticasone propionate	flixotide accuhaler 500 500microgram/inhalation inhalation powder (allen & hanburys ltd)
23741	corticosteroids (for respiratory conditions)	budesonide	novolizer budesonide 200microgram/actuation pressurised inhalation (meda pharmaceuticals ltd)
3988	unknown		flixotide diskhaler-community pack 100 mcg
35724	corticosteroids (for respiratory conditions)	budesonide	budelin novolizer 200micrograms/dose inhalation powder refill (meda pharmaceuticals ltd)
1380	corticosteroids (in chronic bowel disorders)	budesonide	entocort cr 3mg capsules (astrazeneca uk ltd)
56144	corticosteroids (in chronic bowel disorders)	budesonide	budenofalk 9mg gastro-resistant granules sachets (dr. falk pharma uk ltd)
56475	corticosteroids (for respiratory conditions)	fluticasone propionate	flixotide 50micrograms/dose accuhaler (sigma pharmaceuticals plc)
40057	corticosteroids (for respiratory conditions)	budesonide	pulmicort 200micrograms/dose inhaler cfc free (astrazeneca uk ltd)
1236	corticosteroids (for respiratory conditions)	beclometasone dipropionate	becloforte 250micrograms/dose inhaler (glaxosmithkline uk ltd)

For peer review only

Product code	BNF header	Drug substance	Drug product
11497	corticosteroids (for respiratory conditions)	beclometasone dipropionate	beclometasone 400micrograms/dose dry powder inhaler
35408	corticosteroids (for respiratory conditions)	beclometasone dipropionate	becodisks 100microgram (glaxosmithkline uk ltd)
34315	corticosteroids (for respiratory conditions)	beclometasone dipropionate	beclometasone 250microgram/actuation inhalation powder (actavis uk ltd)
956	corticosteroids (for respiratory conditions)	budesonide	pulmicort 200 turbohaler (astrazeneca uk ltd)
18394	corticosteroids (for respiratory conditions)	beclometasone dipropionate	bdp 50microgram/actuation spacehaler (celltech pharma europe ltd)
14321	corticosteroids (for respiratory conditions)	beclometasone dipropionate	beclometasone 200micrograms/dose inhaler cfc free
11732	corticosteroids (for respiratory conditions)	beclometasone dipropionate	beclometasone 50micrograms/dose breath actuated inhaler cfc free
3993	corticosteroids (for respiratory conditions)	beclometasone dipropionate	filair forte 250micrograms/dose inhaler (meda pharmaceuticals ltd)
35225	corticosteroids (for respiratory conditions)	fluticasone propionate	flixotide 100microgram disks with diskhaler (glaxosmithkline uk ltd)
5521	corticosteroids (for respiratory conditions)	beclometasone dipropionate	beclometasone 200micrograms/dose dry powder inhaler
4601	corticosteroids (for respiratory conditions)	beclometasone dipropionate	asmabec 100 clickhaler (focus pharmaceuticals ltd)
16148	corticosteroids (for respiratory conditions)	beclometasone dipropionate	clenil modulite 250micrograms/dose inhaler (chiesi ltd)
2892	corticosteroids (for respiratory conditions)	beclometasone dipropionate	becloforte 400microgram disks (glaxosmithkline uk ltd)
35071	corticosteroids (for respiratory conditions)	beclometasone dipropionate	becodisks 200microgram (glaxosmithkline uk ltd)
35510	corticosteroids (for respiratory conditions)	budesonide	budesonide 200micrograms/dose dry powder inhalation cartridge with device
14567	corticosteroids (for respiratory conditions)	beclometasone dipropionate	asmabec 250 clickhaler (focus pharmaceuticals ltd)
56498	corticosteroids (for respiratory conditions)	budesonide	pulmicort 200 turbohaler (waymade healthcare plc)
24660	unknown		betamethasone valerate
51997	corticosteroids (in chronic bowel disorders)	budesonide	budesonide 9mg gastro-resistant granules sachets
1734	corticosteroids (for respiratory conditions)	beclometasone dipropionate	beclometasone 100micrograms/dose breath actuated inhaler
36090	corticosteroids (for respiratory conditions)	fluticasone propionate	flixotide 100microgram disks (glaxosmithkline uk ltd)
5975	corticosteroids (for respiratory conditions)	fluticasone propionate	fluticasone 125micrograms/dose inhaler cfc free
19401	corticosteroids (for respiratory conditions)	beclometasone dipropionate	beclometasone 250micrograms/actuation inhaler and compact spacer

For peer review only

Product code	BNF header	Drug substance	Drug product
895	corticosteroids (for respiratory conditions)	beclometasone dipropionate	beclazone 100 easi-breathe inhaler (teva uk ltd)
51815	corticosteroids (for respiratory conditions)	fluticasone propionate	flixotide 250micrograms/dose evohaler (waymade healthcare plc)
36021	corticosteroids (for respiratory conditions)	fluticasone propionate	fluticasone propionate 50microgram inhalation powder blisters with device
11198	corticosteroids (for respiratory conditions)	beclometasone dipropionate	beclometasons 50 micrograms/actuation vortex inhaler
50287	corticosteroids (for respiratory conditions)	beclometasone dipropionate	qvar 100 inhaler (de pharmaceuticals)
34428	corticosteroids (for respiratory conditions)	beclometasone dipropionate	beclometasone 50microgram/actuation inhalation powder (neo laboratories ltd)
26063	corticosteroids (for respiratory conditions)	beclometasone dipropionate	beclometasone 100micrograms/dose inhaler (teva uk ltd)
35602	corticosteroids (for respiratory conditions)	budesonide	budesonide 200micrograms/dose dry powder inhalation cartridge
30238	corticosteroids (for respiratory conditions)	beclometasone dipropionate	beclometasone 50microgram/actuation pressurised inhalation (approved prescription services ltd)
14700	corticosteroids (for respiratory conditions)	budesonide	budesonide 400micrograms/actuation inhaler
38	corticosteroids (for respiratory conditions)	beclometasone dipropionate	beclometasone 100micrograms/dose inhaler
13815	corticosteroids (for respiratory conditions)	beclometasone dipropionate	beclazone 100microgram/actuation inhalation powder (actavis uk ltd)
947	corticosteroids (for respiratory conditions)	budesonide	budesonide 50micrograms/actuation refill canister
10321	unknown	budesonide	budesonide 400microgram inhalation powder capsules
1426	corticosteroids (for respiratory conditions)	fluticasone propionate	flixotide 500microgram disc (allen & hanburys ltd)
3150	corticosteroids (for respiratory conditions)	beclometasone dipropionate	beclometasone 100micrograms/actuation extrafine particle cfc free inhaler
35113	corticosteroids (for respiratory conditions)	beclometasone dipropionate	beclometasone 200microgram inhalation powder blisters
1424	corticosteroids (for respiratory conditions)	fluticasone propionate	flixotide 250microgram disc (allen & hanburys ltd)
4803	corticosteroids (for respiratory conditions)	beclometasone dipropionate	beclazone 250microgram/actuation inhalation powder (actavis uk ltd)
56493	corticosteroids (for respiratory conditions)	beclometasone dipropionate	qvar 50micrograms/dose easi-breathe inhaler (sigma pharmaceuticals plc)
61664	corticosteroids (for respiratory conditions)	beclometasone dipropionate	clenil modulite 250micrograms/dose inhaler (waymade healthcare plc)

For peer review only

Product code	BNF header	Drug substance	Drug product
35772	corticosteroids (for respiratory conditions)	fluticasone propionate	fluticasone propionate 100microgram inhalation powder blisters
3119	corticosteroids (for respiratory conditions)	beclometasone dipropionate	becloforte integra 250microgram/actuation inhaler with compact spacer (glaxo laboratories ltd)
5522	corticosteroids (for respiratory conditions)	beclometasone dipropionate	beclometasone 100micrograms/dose dry powder inhaler
25204	corticosteroids (for respiratory conditions)	beclometasone dipropionate	beclometasone 100micrograms/dose inhaler (a a h pharmaceuticals ltd)
7724	corticosteroids (for respiratory conditions)	betamethasone valerate	betamethasone valerate 100micrograms/actuation inhaler
3743	corticosteroids (for respiratory conditions)	beclometasone dipropionate	filair 50 inhaler (meda pharmaceuticals ltd)
1956	corticosteroids (for respiratory conditions)	budesonide	pulmicort 1mg respules (astrazeneca uk ltd)
56499	corticosteroids (for respiratory conditions)	fluticasone propionate	flixotide 500micrograms/dose accuhaler (waymade healthcare plc)
2125	corticosteroids (for respiratory conditions)	budesonide	pulmicort 200microgram refill canister (astrazeneca uk ltd)
36401	corticosteroids (for respiratory conditions)	fluticasone propionate	fluticasone propionate 250microgram inhalation powder blisters with device
4688	corticosteroids (for respiratory conditions)	fluticasone propionate	fluticasone 50microgram/actuation pressurised inhalation
3065	corticosteroids (for respiratory conditions)	betamethasone valerate	bextasol inhalation powder (allen & hanburys ltd)
35118	corticosteroids (for respiratory conditions)	beclometasone dipropionate	becodisks 400microgram with diskhaler (glaxosmithkline uk ltd)
1518	corticosteroids (for respiratory conditions)	fluticasone propionate	flixotide 50microgram/actuation inhalation powder (allen & hanburys ltd)
5309	corticosteroids (for respiratory conditions)	fluticasone propionate	flixotide 50micrograms/dose evohaler (glaxosmithkline uk ltd)
47225	unknown	budesonide	budesonide 9mg gastro-resistant granules sachets
30649	corticosteroids (for respiratory conditions)	budesonide	easyhaler budesonide 400micrograms/dose dry powder inhaler (orion pharma (uk) ltd)
35580	corticosteroids (for respiratory conditions)	beclometasone dipropionate	beclometasone 100microgram inhalation powder blisters with device
41412	corticosteroids (for respiratory conditions)	beclometasone dipropionate	beclometasone 400micrograms/actuation inhaler
20825	corticosteroids (for respiratory conditions)	beclometasone dipropionate	spacehaler bdp 250microgram/actuation spacehaler (celltech pharma europe ltd)

For peer review only

Product code	BNF header	Drug substance	Drug product
35461	corticosteroids (for respiratory conditions)	fluticasone propionate	flixotide 250microgram disks with diskhaler (glaxosmithkline uk ltd)
42985	corticosteroids (for respiratory conditions)	fluticasone propionate	flixotide 50micrograms/dose accuhaler (glaxosmithkline uk ltd)
9921	corticosteroids (for respiratory conditions)	beclometasone dipropionate	beclometasone 100micrograms/dose breath actuated inhaler cfc free
56474	corticosteroids (for respiratory conditions)	fluticasone propionate	flixotide 125micrograms/dose evohaler (de pharmaceuticals)
35986	corticosteroids (for respiratory conditions)	fluticasone propionate	flixotide 50microgram disks (glaxosmithkline uk ltd)
11149	glucocorticoid therapy	betamethasone	betnelan 500microgram tablets (focus pharmaceuticals ltd)
1243	corticosteroids (for respiratory conditions)	beclometasone dipropionate	beclazone 250 easi-breathe inhaler (teva uk ltd)
31774	corticosteroids (for respiratory conditions)	beclometasone dipropionate	beclometasone 50micrograms/dose inhaler (generics (uk) ltd)
56477	corticosteroids (for respiratory conditions)	fluticasone propionate	flixotide 100micrograms/dose accuhaler (waymade healthcare plc)
2600	corticosteroids (for respiratory conditions)	beclometasone dipropionate	beclometasone 250micrograms/dose breath actuated inhaler
16151	corticosteroids (for respiratory conditions)	beclometasone dipropionate	clenil modulite 200micrograms/dose inhaler (chiesi ltd)
1100	corticosteroids (for respiratory conditions)	beclometasone dipropionate	beclazone 100 inhaler (teva uk ltd)
1951	corticosteroids (for respiratory conditions)	beclometasone dipropionate	becodisks 400microgram disc (allen & hanburys ltd)
7948	corticosteroids (for respiratory conditions)	fluticasone propionate	fluticasone propionate 250micrograms/dose dry powder inhaler
21482	corticosteroids (for respiratory conditions)	beclometasone dipropionate	beclometasone 100micrograms/dose inhaler (generics (uk) ltd)
9477	corticosteroids (for respiratory conditions)	beclometasone dipropionate	asmabec 100microgram/actuation spacehaler (celltech pharma europe ltd)
27679	corticosteroids (for respiratory conditions)	beclometasone dipropionate	beclometasone 100microgram/actuation pressurised inhalation (approved prescription services ltd)
35652	corticosteroids (for respiratory conditions)	beclometasone dipropionate	beclometasone 100microgram inhalation powder blisters
46157	corticosteroids (for respiratory conditions)	beclometasone dipropionate	beclometasone 200 cyclocaps (teva uk ltd)
56484	corticosteroids (for respiratory conditions)	fluticasone propionate	flixotide 250micrograms/dose accuhaler (waymade healthcare plc)
3442	unknown		pulmicort complete 200 mcg inh

For peer review only

Product code	BNF header	Drug substance	Drug product
28761	corticosteroids (for respiratory conditions)	beclometasone dipropionate	spacehaler bdp 50microgram/actuation spacehaler (celltech pharma europe ltd)
5992	corticosteroids (for respiratory conditions)	beclometasone dipropionate	beclometasone 50micrograms/dose dry powder inhaler
454	corticosteroids (for respiratory conditions)	budesonide	pulmicort 200microgram inhaler (astrazeneca uk ltd)
3220	corticosteroids (for respiratory conditions)	beclometasone dipropionate	qvar 50 autohaler (teva uk ltd)
18537	unknown	budesonide	budesonide 200microgram inhalation powder capsules
57555	corticosteroids (for respiratory conditions)	fluticasone propionate	flixotide 125micrograms/dose evohaler (dowelhurst ltd)
48709	corticosteroids (for respiratory conditions)	beclometasone dipropionate	qvar 100micrograms/dose easi-breathe inhaler (sigma pharmaceuticals plc)
16584	corticosteroids (for respiratory conditions)	beclometasone dipropionate	beclometasone 50micrograms/dose inhaler cfc free
35392	corticosteroids (for respiratory conditions)	fluticasone propionate	flixotide 500microgram disks with diskhaler (glaxosmithkline uk ltd)

For peer review only

FP_SAL codes

Product code	BNF header	Drug substance	Drug product
638	Selective Beta 2 Agonists/Corticosteroids (for Respiratory Conditions)	Fluticasone propionate/Salmeterol xinafoate	Seretide 250 Accuhaler (GlaxoSmithKline UK Ltd)
665	Selective Beta 2 Agonists/Corticosteroids (for Respiratory Conditions)	Salmeterol xinafoate/Fluticasone propionate	Seretide 100 Accuhaler (GlaxoSmithKline UK Ltd)
3666	Selective Beta 2 Agonists/Corticosteroids (for Respiratory Conditions)	Salmeterol xinafoate/Fluticasone propionate	Seretide 500 Accuhaler (GlaxoSmithKline UK Ltd)
5143	Selective Beta 2 Agonists/Corticosteroids (for Respiratory Conditions)	Fluticasone propionate/Salmeterol xinafoate	Seretide 50 Evohaler (GlaxoSmithKline UK Ltd)
5161	Selective Beta 2 Agonists/Corticosteroids (for Respiratory Conditions)	Fluticasone propionate/Salmeterol xinafoate	Seretide 125 Evohaler (GlaxoSmithKline UK Ltd)
5172	Selective Beta 2 Agonists/Corticosteroids (for Respiratory Conditions)	Fluticasone propionate/Salmeterol xinafoate	Seretide 250 Evohaler (GlaxoSmithKline UK Ltd)
5558	Selective Beta 2 Agonists/Corticosteroids (for Respiratory Conditions)	Salmeterol Xinafoate/Fluticasone Propionate	Salmeterol 50micrograms with fluticasone 500micrograms CFC free inhaler
5864	Selective Beta 2 Agonists/Corticosteroids (for Respiratory Conditions)	Salmeterol Xinafoate/Fluticasone Propionate	Salmeterol 25micrograms with fluticasone 250micrograms CFC free inhaler
5942	Selective Beta 2 Agonists/Corticosteroids (for Respiratory Conditions)	Salmeterol Xinafoate/Fluticasone Propionate	Salmeterol 50micrograms with fluticasone 250micrograms CFC free inhaler
6569	Selective Beta 2 Agonists/Corticosteroids (for Respiratory Conditions)	Salmeterol Xinafoate/Fluticasone Propionate	Salmeterol 25micrograms with fluticasone 125micrograms CFC free inhaler
6616	Selective Beta 2 Agonists/Corticosteroids (for Respiratory Conditions)	Salmeterol Xinafoate/Fluticasone Propionate	Salmeterol 25micrograms with fluticasone 50micrograms CFC free inhaler
6938	Selective Beta 2 Agonists/Corticosteroids (for Respiratory Conditions)	Salmeterol Xinafoate/Fluticasone Propionate	Salmeterol 50micrograms with fluticasone 100micrograms dry powder inhaler
11410	Selective Beta 2 Agonists/Corticosteroids (for Respiratory Conditions)	Salmeterol xinafoate/Fluticasone propionate	Fluticasone propionate 500micrograms/dose / Salmeterol 50micrograms/dose dry powder inhaler
11588	Selective Beta 2 Agonists/Corticosteroids (for Respiratory Conditions)	Fluticasone propionate/Salmeterol xinafoate	Fluticasone 125micrograms/dose / Salmeterol 25micrograms/dose inhaler CFC free
11618	Selective Beta 2 Agonists/Corticosteroids (for Respiratory Conditions)	Fluticasone propionate/Salmeterol xinafoate	Fluticasone 250micrograms/dose / Salmeterol 25micrograms/dose inhaler CFC free
12994	Selective Beta 2 Agonists/Corticosteroids (for Respiratory Conditions)	Fluticasone propionate/Salmeterol xinafoate	Fluticasone 50micrograms/dose / Salmeterol 25micrograms/dose inhaler CFC free

For peer review only

Product code	BNF header	Drug substance	Drug product
13040	Selective Beta 2 Agonists/Corticosteroids (for Respiratory Conditions)	Fluticasone propionate/Salmeterol xinafoate	Fluticasone propionate 250micrograms/dose / Salmeterol 50micrograms/dose dry powder inhaler
13273	Selective Beta 2 Agonists/Corticosteroids (for Respiratory Conditions)	Salmeterol xinafoate/Fluticasone propionate	Fluticasone propionate 100micrograms/dose / Salmeterol 50micrograms/dose dry powder inhaler
48739	Selective Beta 2 Agonists/Corticosteroids (for Respiratory Conditions)	Fluticasone propionate/Salmeterol xinafoate	Seretide 250 Evohaler (DE Pharmaceuticals)
49000	Selective Beta 2 Agonists/Corticosteroids (for Respiratory Conditions)	Fluticasone propionate/Salmeterol xinafoate	Seretide 250 Evohaler (Waymade Healthcare Plc)
50560	Selective Beta 2 Agonists/Corticosteroids (for Respiratory Conditions)	Fluticasone propionate/Salmeterol xinafoate	Seretide 250 Accuhaler (Sigma Pharmaceuticals Plc)
50689	Selective Beta 2 Agonists/Corticosteroids (for Respiratory Conditions)	Fluticasone propionate/Formoterol fumarate dihydrate	Flutiform 50micrograms/dose / 5micrograms/dose inhaler (Napp Pharmaceuticals Ltd)
50886	Selective Beta 2 Agonists/Corticosteroids (for Respiratory Conditions)	Fluticasone propionate/Salmeterol xinafoate	Seretide 250 Evohaler (Stephar (U.K.) Ltd)
51027	Selective Beta 2 Agonists/Corticosteroids (for Respiratory Conditions)	Fluticasone propionate/Salmeterol xinafoate	Seretide 125 Evohaler (DE Pharmaceuticals)
51151	Selective Beta 2 Agonists/Corticosteroids (for Respiratory Conditions)	Fluticasone propionate/Salmeterol xinafoate	Seretide 125 Evohaler (Lexon (UK) Ltd)
51270	Selective Beta 2 Agonists/Corticosteroids (for Respiratory Conditions)	Fluticasone propionate/Formoterol fumarate dihydrate	Fluticasone 50micrograms/dose / Formoterol 5micrograms/dose inhaler CFC free
51394	Selective Beta 2 Agonists/Corticosteroids (for Respiratory Conditions)	Salmeterol xinafoate/Fluticasone propionate	Seretide 500 Accuhaler (Waymade Healthcare Plc)
51593	Selective Beta 2 Agonists/Corticosteroids (for Respiratory Conditions)	Salmeterol xinafoate/Fluticasone propionate	Seretide 500 Accuhaler (DE Pharmaceuticals)
51861	Selective Beta 2 Agonists/Corticosteroids (for Respiratory Conditions)	Salmeterol xinafoate/Fluticasone propionate	Seretide 500 Accuhaler (Mawdsley-Brooks & Company Ltd)
51909	Selective Beta 2 Agonists/Corticosteroids (for Respiratory Conditions)	Fluticasone propionate/Salmeterol xinafoate	Seretide 250 Evohaler (Necessity Supplies Ltd)
53230	Selective Beta 2 Agonists/Corticosteroids (for Respiratory Conditions)	Fluticasone propionate/Salmeterol xinafoate	Seretide 250 Accuhaler (DE Pharmaceuticals)
53283	Selective Beta 2 Agonists/Corticosteroids (for Respiratory Conditions)	Salmeterol xinafoate/Fluticasone propionate	Seretide 100 Accuhaler (Waymade Healthcare Plc)
55411	Corticosteroids Used In Nasal Allergy/Antihistamines In Nasal Allergy	Fluticasone propionate/Azelastine hydrochloride	Fluticasone propionate 50micrograms/dose / Azelastine 137micrograms/dose nasal spray
55435	Corticosteroids Used In Nasal Allergy/Antihistamines In Nasal Allergy	Fluticasone propionate/Azelastine hydrochloride	Dymista 137micrograms/dose / 50micrograms/dose nasal spray (Meda Pharmaceuticals Ltd)

For peer review only

Product code	BNF header	Drug substance	Drug product
55677	Selective Beta 2 Agonists/Corticosteroids (for Respiratory Conditions)	Salmeterol xinafoate/Fluticasone propionate	Seretide 500 Accuhaler (Lexon (UK) Ltd)
61280	Selective Beta 2 Agonists/Corticosteroids (for Respiratory Conditions)	Fluticasone propionate/Salmeterol xinafoate	Seretide 250 Accuhaler (Waymade Healthcare Plc)
62126	Selective Beta 2 Agonists/Corticosteroids (for Respiratory Conditions)	Salmeterol xinafoate/Fluticasone propionate	Seretide 100 Accuhaler (DE Pharmaceuticals)
63252	Selective Beta 2 Agonists/Corticosteroids (for Respiratory Conditions)	Fluticasone propionate/Salmeterol xinafoate	Seretide 250 Evohaler (Lexon (UK) Ltd)
63945	Selective Beta 2 Agonists/Corticosteroids (for Respiratory Conditions)	Fluticasone propionate/Salmeterol xinafoate	Seretide 250 Accuhaler (Lexon (UK) Ltd)
64372	Selective Beta 2 Agonists/Corticosteroids (for Respiratory Conditions)	Fluticasone propionate/Salmeterol xinafoate	Sirdupla 25micrograms/dose / 125micrograms/dose inhaler (Mylan Ltd)
64373	Selective Beta 2 Agonists/Corticosteroids (for Respiratory Conditions)	Fluticasone propionate/Salmeterol xinafoate	Sirdupla 25micrograms/dose / 250micrograms/dose inhaler (Mylan Ltd)
65117	Selective Beta 2 Agonists/Corticosteroids (for Respiratory Conditions)	Fluticasone propionate/Salmeterol xinafoate	Seretide 125 Evohaler (Mawdsley-Brooks & Company Ltd)

For peer review only

STROBE 2007 (v4) Statement—Checklist of items that should be included in reports of *cohort studies*

Section/Topic	Item #	Recommendation	Reported on page #
**Title and abstract**	1	(*a*) Indicate the study's design with a commonly used term in the title or the abstract	1
		(*b*) Provide in the abstract an informative and balanced summary of what was done and what was found	3 (note: is a protocol for an observational study so here and subsequently is reported *what is planned to be done* only)
**Introduction**			
Background/rationale	2	Explain the scientific background and rationale for the investigation being reported	5
Objectives	3	State specific objectives, including any prespecified hypotheses	6
**Methods**			
Study design	4	Present key elements of study design early in the paper	6
Setting	5	Describe the setting, locations, and relevant dates, including periods of recruitment, exposure, follow-up, and data collection	6
Participants	6	(*a*) Give the eligibility criteria, and the sources and methods of selection of participants. Describe methods of follow-up	7 - 10
		(*b*) For matched studies, give matching criteria and number of exposed and unexposed	7 - 10
Variables	7	Clearly define all outcomes, exposures, predictors, potential confounders, and effect modifiers. Give diagnostic criteria, if applicable	13 - 14
Data sources/ measurement	8*	For each variable of interest, give sources of data and details of methods of assessment (measurement). Describe comparability of assessment methods if there is more than one group	6
Bias	9	Describe any efforts to address potential sources of bias	14 - 16
Study size	10	Explain how the study size was arrived at	14
Quantitative variables	11	Explain how quantitative variables were handled in the analyses. If applicable, describe which groupings were chosen and why	11

For peer review only

Statistical methods	12	(*a*) Describe all statistical methods, including those used to control for confounding	14 - 16
		(*b*) Describe any methods used to examine subgroups and interactions	14 - 16
		(*c*) Explain how missing data were addressed	16
		(*d*) If applicable, explain how loss to follow-up was addressed	NA
		(*e*) Describe any sensitivity analyses	14 - 16
**Results**			***NA (is a protocol for an observational study)***
Participants	13*	(a) Report numbers of individuals at each stage of study—eg numbers potentially eligible, examined for eligibility, confirmed eligible, included in the study, completing follow-up, and analysed	
		(b) Give reasons for non-participation at each stage	
		(c) Consider use of a flow diagram	
Descriptive data	14*	(a) Give characteristics of study participants (eg demographic, clinical, social) and information on exposures and potential confounders	
		(b) Indicate number of participants with missing data for each variable of interest	
		(c) Summarise follow-up time (eg, average and total amount)	
Outcome data	15*	Report numbers of outcome events or summary measures over time	
Main results	16	(*a*) Give unadjusted estimates and, if applicable, confounder-adjusted estimates and their precision (eg, 95% confidence interval). Make clear which confounders were adjusted for and why they were included	
		(*b*) Report category boundaries when continuous variables were categorized	
		(*c*) If relevant, consider translating estimates of relative risk into absolute risk for a meaningful time period	
Other analyses	17	Report other analyses done—eg analyses of subgroups and interactions, and sensitivity analyses	
**Discussion**			***NA (is a protocol for an observational study)***
Key results	18	Summarise key results with reference to study objectives	
**Limitations**			***NA (is a protocol for an observational study)***
Interpretation	20	Give a cautious overall interpretation of results considering objectives, limitations, multiplicity of analyses, results from similar studies, and other relevant evidence	

For peer review only

Generalisability	21	Discuss the generalisability (external validity) of the study results	
**Other information**			
Funding	22	Give the source of funding and the role of the funders for the present study and, if applicable, for the original study on which the present article is based	2

*Give information separately for cases and controls in case-control studies and, if applicable, for exposed and unexposed groups in cohort and cross-sectional studies.

Note: An Explanation and Elaboration article discusses each checklist item and gives methodological background and published examples of transparent reporting. The STROBE checklist is best used in conjunction with this article (freely available on the Web sites of PLoS Medicine at http://www.plosmedicine.org/, Annals of Internal Medicine at http://www.annals.org/, and Epidemiology at http://www.epidem.com/). Information on the STROBE Initiative is available at www.strobe-statement.org.

For peer review only

Real world effects of medications for chronic obstructive pulmonary disease: protocol for a UK population-based non-interventional cohort study with validation against randomised trial results

Journal:	*BMJ Open*
Manuscript ID	bmjopen-2017-019475.R2
Article Type:	Protocol
Date Submitted by the Author:	24-Jan-2018
Complete List of Authors:	Wing, Kevin; London School of Hygiene and Tropical Medicine,<br>Williamson, Elizabeth; London School of Hygiene and Tropical Medicine,<br>Carpenter, James; London School of Hygiene and Tropical Medicine<br>Wise, Lesley; London School of Hygiene and Tropical Medicine<br>Schneeweiss, Sebastian; Brigham and Womens' Hospital, Harvard Medical School<br>Smeeth, Liam; London School of Hygiene and Tropical Medicine<br>Quint, Jennifer; Imperial College London<br>Douglas, Ian; London School of Hygiene and Tropical Medicine
**Primary Subject Heading**:	Epidemiology
Secondary Subject Heading:	Evidence based practice, Research methods, Respiratory medicine, Public health, Pharmacology and therapeutics
Keywords:	Chronic airways disease < THORACIC MEDICINE, Epidemiology < THORACIC MEDICINE, CLINICAL PHARMACOLOGY, EPIDEMIOLOGY, PRIMARY CARE, PUBLIC HEALTH

SCHOLARONE™
Manuscripts

TITLE PAGE

Title: Real world effects of medications for chronic obstructive pulmonary disease: protocol for a UK population-based non-interventional cohort study with validation against randomised trial results

Authors:

Kevin Wing[1*]

Elizabeth Williamson[2]

James Carpenter[2]

Lesley Wise[1]

Sebastian Schneeweiss[3,4]

Liam Smeeth[1]

Jennifer Quint[5]

Ian Douglas[1]

[1]Department of Non-communicable Disease Epidemiology, Faculty of Epidemiology and Population Health, London School of Hygiene and Tropical Medicine, London, UK

[2]Department of Medical Statistics, Faculty of Epidemiology and Population Health, London School of Hygiene and Tropical Medicine, London, UK

[3]Department of Epidemiology, Harvard Medical School.

[4]Division of Pharmacoepidemiology and Pharmacoeconomics, Department of Medicine, Brigham & Women's Hospital

[5]National Heart and Lung Institute, Imperial College London, London, United Kingdom

***Submitting and corresponding author:** Kevin Wing (kevin.wing@lshtm.ac.uk), London School of Hygiene and Tropical Medicine, Keppel Street, London WC1E 7HT. Tel: +44 20 7636 8636, Fax: +44 20 7436 5389

For peer review only

Funding statement

This work was supported by NIHR grant number 15/80/28

Competing interests statement

Dr Wing and Dr Williamson declare no competing interests.

Professor Carpenter is funded 60% by a grant from the MRC, via a secondment to the MRC Clinical Trials Unit. The remaining funding is a combination of HEFCE and small contributions from various NIHR, ESRC, MRC and EU funds. He undertakes methodological consultancy work for Novartis and GlaxoSmithKline, and has given missing data courses for GlaxoSmithKline, Bayer and Boehringer. Professor Smeeth reports grants from Wellcome, MRC, NIHR, BHF, Diabetes UK, ESRC and the EU; grants and personal fees for advisory work from GSK, and personal fees for advisory work from AstraZeneca. He is a Trustee of the British Heart Foundation.

Dr Wise is an independent consultant to the pharmaceutical industry and is employed to provide advice by a number of different companies none of which is involved in this therapeutic area.

Professor Schneeweiss is a consultant to WHISCON, LLC and to Aetion, Inc., a software manufacturer of which he also owns equity. He is principal investigator of investigator-initiated grants to the Brigham and Women's Hospital from Bayer, Genentech and Boehringer Ingelheim unrelated to the topic of this study. He does not receive personal fees from biopharmaceutical companies.

Professor Smeeth reports grants from Wellcome, MRC, NIHR, BHF, Diabetes UK, ESRC and the EU; grants and personal fees for advisory work from GSK, and personal fees for advisory work from AstraZeneca. He is a Trustee of the British Heart Foundation.

Dr Quint's research group has received funding from MRC, Wellcome, BLF, GSK, BI, AZ and Insmed for other projects, none of which relate to this work.

Dr Douglas is funded by an unrestricted grant from, has consulted for and holds stock in GlaxoSmithKline.

Word count (not including abstract, tables, figures, or footnotes): 4386

Statement about prior postings and presentations: This work has not been published elsewhere.

For peer review only

Abstract

Introduction

Chronic obstructive pulmonary disease (COPD) is a progressive disease affecting 3 million people in the UK, in which patients exhibit airflow obstruction that is not fully reversible. COPD treatment guidelines are largely informed by randomised controlled trial (RCT) results, but it is unclear if these findings apply to large patient populations not studied in trials. Non-interventional studies could be used to study patient groups excluded from trials, but the use of these studies to estimate treatment effectiveness is in its infancy. In this study we will use individual trial data to validate non-interventional methods for assessing COPD treatment effectiveness, before applying these methods to the analysis of treatment effectiveness within people excluded from, or under-represented in COPD trials.

Methods and analysis

Using individual patient data from the landmark COPD TORCH trial and validated methods for detecting COPD and exacerbations in routinely collected primary care data, we will assemble a cohort in the UK Clinical Practice Research Datalink (selecting people from between the dates of 1st January 2004 and 1st January 2017) with similar characteristics to TORCH participants and test whether non-interventional data can generate comparable results to trials, using cohort methodology with propensity score techniques to adjust for potential confounding. We will then use the methodological template we have developed to determine risks and benefits of COPD treatments in people excluded from TORCH. Outcomes are pneumonia, COPD exacerbation, mortality and time to treatment change. Groups to be studied include the elderly (>80 years), people with substantial comorbidity, people with and without underlying cardiovascular disease and people with mild COPD.

Ethics and dissemination

Ethical approval has been granted by the LSHTM Medicines Ethics Committee (Ref: 11997). The study is under review by the Independent Scientific Advisory Committee (ISAC) of the UK Medicines and Healthcare Products Regulatory Agency (MHRA). An application to use the TORCH trial data made to clinicalstudydatarequest.com has been approved. In addition to scientific publications, dissemination methods will be developed based on discussions with COPD patient groups.

For peer review only

Strengths and limitations of this study

Strengths

- Large cohort study
- Use of validated methods for detecting COPD within the Clinical Practice Research Datatlink
- Use of randomised controlled trial (RCT) individual patient data to assess ability of non-interventional methods to detect COPD treatment effects within an RCT-analagous population

Limitations

- Adherence to medication will need to be assessed based on proxy variables (e.g. time covered by prescription)

For peer review only

Introduction

Background and rationale

Chronic obstructive pulmonary disease (COPD) affects 3 million people in the UK.[1] The most common cause is smoking, and patients exhibit airflow obstruction that is not fully reversible. The disease is progressive, with declining lung function and a worsening of symptoms. Most troublesome are acute exacerbations manifested as a sudden worsening of symptoms e.g. severe coughing, shortness of breath and chest congestion, requiring urgent treatment, and possibly hospitalisation. Whilst smoking cessation remains the most effective intervention, the rate of exacerbation can be reduced by regular medication such as combination long-acting beta-agonists (LABA) and inhaled corticosteroids (ICS) or long-acting muscarinic antagonists (LAMAs).[2,3]

COPD treatment guidelines are largely informed by randomised controlled trial (RCT) results,[4] but it is not clear if these findings apply to large patient populations not studied in trials. Fluticasone propionate + salmeterol (FP/SAL) is a LABA/ICS combination and is one of the most widely used COPD treatments. It was studied in large randomised trials (e.g. the TORCH trial),[2] but the effects of treatment in important patient groups who were not studied are unknown. Some were excluded from trials (e.g. those >80 years and those with substantial comorbidity) while others are under represented (e.g. people with mild COPD),[2,5] meaning conclusions about these groups are difficult to make.

Whilst the conduct of non-interventional studies (sometimes also referred to as “observational studies”) to investigate possible drug harms is well established, the use of these studies to estimate treatment effectiveness is in its infancy. Issues of treatment channelling and indication bias mean that measuring the intended benefit of a treatment is beset with difficulties. Over the next few years we will see more non-interventional studies of drug effectiveness emerging due to recent legislation requiring pharmaceutical companies to study the real world effects of medications;[6,7] however, rigorous, validated methodology is needed to translate these complex data into reliable evidence. For example, the availability of anonymised individual patient data from randomised controlled trials provides the potential for “RCT-analogous” cohorts to be selected from non-interventional data sources (by matching patient records from non-interventional data to the RCT patient records on key characteristics). If subsequent analysis of a non-interventional RCT-analogous cohort generates results that are similar to those generated by the reference RCT, one could be confident in the validity of the results, and in the non-interventional methods used to obtain these results in this setting.

In this study we will use TORCH[2] individual trial data to validate non-interventional methods for assessing COPD treatment effectiveness, before going on to apply these methods to the analysis of

For peer review only - http://bmjopen.bmj.com/site/about/guidelines.xhtml

treatment effectiveness within people excluded from or underrepresented in the TORCH trial. Non-interventional data will be obtained from the UK Clinical Practice Research Datalink (linked to the Hospital Episodes Statistics database).[8] The results we generate will aid patients, prescribers and policy makers in deciding the most appropriate treatment for COPD for all types of patients. The approach used can also provide a template for treatment effectiveness research using non-interventional data with inbuilt validation against a randomised trial.

Aims and objectives

The aims of our study are (1) to measure the association between treatments for COPD and a number of COPD outcomes including exacerbation rate, mortality, pneumonia, and time to treatment change amongst patients not included in randomised clinical trials for COPD treatments and (2) to develop a methodological framework with in built validation against RCT data, for using non-interventional electronic health records (EHR) to answer questions about drug treatment effects (i.e. both benefits and risks).

Specific objectives are to: (1) validate methods for measuring COPD medication effectiveness in EHR data by comparing with trial results; (2) use EHR data to measure COPD medication effectiveness in patients excluded from trials (most importantly those >80 years or with substantial comorbidity) and (3) determine COPD treatment effectiveness in an under-studied disease stage (mild COPD).

Methods and analysis

Figure 1 provides a high-level overview of the study, detailing each objective and data source used, and showing how existing RCT data will be used in Objective 1 in order to validate methods for analysing COPD in routinely collected electronic health data that will then be applied to unanswered questions in Objective 2 and 3.

Study design

We have chosen a cohort study design as it will allow us to measure the effects of prescribing different treatments for COPD on future outcomes in different types of patients. Eligibility criteria for cohort entry will vary between objectives (detailed in the selection of participants section below).

Setting/data sources

For peer review only

Patient data used in this study will be obtained from two different sources: the TORCH randomised trial and the UK Clinical Practices Research Datalink (CPRD) (linked to Hospital Episodes Statistics – HES – data).

TORCH

TORCH was a placebo controlled randomised trial of the combined inhaler fluticasone propionate (FP) + salmeterol (SAL) (FP/SAL) for the treatment of COPD, published in 2007. Patients were randomised to receive FP/SAL, FP alone, SAL alone or placebo and the primary comparison of interest was between FP/SAL and placebo.[2] Key outcomes were expected benefits (rate of COPD exacerbation and mortality) and an expected harm due to the immunosuppressive action of the corticosteroid FP (pneumonia). Whilst findings for the primary endpoint of mortality were null, this was thought to be due to poor statistical power as a result of a lower than anticipated mortality rate. Nonetheless, a lower rate of exacerbations was seen with FP/SAL, and a higher rate of pneumonia was observed. As one of the largest trials in COPD, and with three year follow up, TORCH is a landmark study, providing a validation point for our study. We will obtain individual patient data from the TORCH study via www.clinicalstudydatarequest.com for use in Objective 1 (see selection of participants section below)

CPRD

The CPRD is a very large database of prospectively collected, anonymised UK population-based electronic health records. CPRD primary care records comprise ~8-10% of the UK population and contain comprehensive information on clinical diagnoses, prescribing, referrals, tests and demographic/lifestyle factors.[8] In order to contribute to the database, general practices and other health centres must meet prespecified standards for research-quality data (i.e. be “up to standard”). Data quality/validity are therefore high and the data are nationally representative.[8,9] A patient starts contributing follow-up time to the database at the date they join an “up to standard” practice (or the date that their practice starts contributing up to standard data), and stop contributing follow-up time on either their death date, their transfer out date (the date that they leave the database due to reasons other than death) or on the last collection date for their practice. Linkage between the primary care records in CPRD and hospital episode statistics (HES) is well established for >60% of practices in the CPRD, providing a data set augmented with detailed secondary care diagnostic and procedural records. Algorithms have been established to identify COPD, COPD exacerbations and pneumonia (both hospital and primary care managed) in CPRD/HES linked data (including validated algorithms for COPD and exacerbations).[10–12] See supplementary materials for a high-level overview of these algorithms.

For peer review only

Figure 1: Overview of study objectives and sources of data for the COPD real-world medicines effects study (RCT=randomised controlled trial, CPRD= the UK Clinical Practice Research Datalink, FP/SAL=Fluticasone propionate + salmeterol)

[FIGURE 1 HERE]

A. Work performed by others prior to this study
Of the total population of people with COPD, only a subset are included in RCTs of COPD treatments, based on the RCT inclusion/exclusion criteria. The RCT generates results that inform clinical practice, and the anonymised raw data for the study can be made available to other researchers via the Clinical Study Data Request website. For this study, the specific COPD treatment-RCT of interest is the TORCH trial,[2] investigating the effect of FP/SAL on COPD exacerbations.

B. Work to be performed as part of this study

1. Objective 1

A cohort of TORCH (RCT)-analogous patients will be selected from the UK Clinical Practice Research Datalink (CPRD), by matching people with COPD within CPRD to the records of people included in the trial. An analysis of the effect of FP/SAL on COPD exacerbations will then be performed on this TORCH-analagous CPRD cohort. If the results obtained are comparable to those obtained in the TORCH trial itself, this will serve as a validation step, showing that data from the non-inverventional ("real-world") CPRD source can reliably be used to study COPD treatment effects.

2. Objective 2

The validated analysis techniques used for Objective 1 will then be used to study people in CPRD who would not have been eligible for inclusion in an RCT due to their age and the presence of other comorbidities, and for whom the effect of FP/SAL is currently unknown.

3. Objective 3

The validated analysis techniques will then be used to study people with mild COPD only, who have been under-represented in RCTs, and for whom the effect of COPD treatments is unclear.

For peer review only

Selection of participants

Participants will be selected from the CPRD from between the dates of 1st January 2004 and 1st January 2017. All patients will need to have been registered with an up-to standard practice for at least 12 months. Participant selection criteria will then vary by objective as detailed below.

Objective 1: Validation of non-interventional methods by comparing with trial results

An overview of each of the steps for participant selection for Objective 1 is provided in Figure 2.

Step 1

We will select all patients in the CPRD with COPD who are eligible for HES-linkage and during the period covered by the linkage would have met the following TORCH study **inclusion** criteria:

- a diagnosis of COPD
- age 40-80 years
- smoking history
- lung function (FEV_1 <60% predicted, FEV_1/FVC ratio<70%)

An eligible-for-inclusion date will then be assigned as the date that all of the above inclusion criteria were met for the individual. We will then exclude any individual who has any of the following TORCH study **exclusion** criteria prior to their eligible-for-inclusion date:

- a diagnosis of asthma (within the previous 5 years)
- a diagnosis for any (non-COPD) respiratory disorder
- a record of lung surgery
- a diagnosis of alpha-1 antitrypsin deficiency
- evidence of drug or alcohol abuse
- a record of having received long-term oxygen therapy
- diagnoses for conditions likely to interfere with the TORCH trial or cause death within 3 years
- current use of oral corticosteroid therapy (defined as continuous use for greater than 6 weeks, with courses of oral corticosteroidsteroids separated by a period of less than 7 days considered as continuous use)
- any exposure to FP/SAL within the previous 4 weeks

Finally, in-line with the TORCH trial approach, anyone who has an exacerbation requiring oral corticosteroid therapy or hospitalisation during the run-in period (the 2-week period following eligibility) will also be excluded.

Feasibility counts in the CPRD indicate there are ~13,000 patients meeting these criteria (Figure 2). Given the limited information on how asthma exclusions were applied in the TORCH study, we will perform a sensitivity analysis in which the asthma exclusion is a diagnosis within the previous 1 year, rather than 5 years as specified above.

For peer review only

Step 2

Next we will determine if/when these patients ever received FP/SAL. During any time between attaining TORCH eligibility and a subsequent prescription for FP/SAL, patients will be eligible for inclusion as an unexposed (to FP/SAL) patient in Objective 1. There may be multiple time periods within a person's record where eligibility as an unexposed patient is met (Figure 3). Feasibility work shows that between 1st January 2004 and 1st January 2017 there were ~11,000 TORCH eligible patients in CPRD who did not receive FP/SAL at the time they attained TORCH eligibility and therefore have at least one time period that means they are eligible for inclusion as an Objective 1 unexposed participant (Figure 2). Individuals in CPRD who have more than one unexposed eligibility period within their record (Figure 3) will be able to contribute more than once to the pool of unexposed participants (with the covariates and person-time contributed unique to the specific unexposed eligibility period).

Step 3

Having obtained individual level patient data for TORCH participants from clinicalstudydatarequest.com, we will then match each TORCH participant (n=6,112) 1:1 with the closest available unexposed patient record in the CPRD pool of FP/SAL untreated patients obtained in Step 2. Matching will be based on the following TORCH baseline characteristics:

- age
- sex
- body mass index
- previous treatment with:
    - inhaled corticosteroids
    - long acting beta-agonists
- history of COPD exacerbations
- history of cardiovascular disease
- lung function

Where an individual from CPRD has multiple unexposed "eligibility periods" that can be matched to a TORCH participant, the CPRD characteristics that will be matched on will be those from the beginning of the specific eligibility period.

Some of the TORCH inclusion criteria will not be fully assessable using CPRD data (e.g. we will be able to assess whether patients are smokers but will not always know their pack year history). Hence the inclusion/exclusion criteria are analogous with TORCH criteria but we acknowledge they are not identical. Identification of criteria will be done based on algorithms already determined and by the identification of clinical codes in the CPRD. For those individuals that have contributed multiple unexposed records to the pool of CPRD unexposed participants (Figure 3), after one of their unexposed records has been matched to a TORCH participant we will remove all of their other unexposed records (meaning that an individual can only appear once in the final TORCH-matched

For peer review only

unexposed cohort). We anticipate matching all or the majority of TORCH participants with a CPRD patient, giving us a pool of TORCH-analogous untreated patients within CPRD, with similar baseline characteristics as TORCH participants at the point of randomisation (n~6,000, Figure 2).

Step 4

Following this, we will select all patients in CPRD meeting the TORCH eligibility criteria specified above, and who also received treatment with FP/SAL (either on the date of eligibility or at a later date). From feasibility work, we anticipate ~12,000 eligible FP/SAL treated patients (Figure 2), some of whom may have multiple time-periods of treated patient eligibility and therefore could contribute more than once to the initial pool of treated patients (Figure 3). In contrast to the unexposed TORCH-eligible cohort, our initial approach will not involve matching participants of the exposed (to FP/SAL) TORCH-eligible cohort with participants from the TORCH trial, as this would negatively impact the ability to calculate propensity scores for receiving FP/SAL in Step 5. Note that there will be overlap between those selected as untreated (in Step 2) and as treated (in Step 4). Approximately 90% of the Step 4 treated patients will also have been eligible as Step 2 untreated patients, as they will have had periods where they were not treated with FP/SAL and met the Step 2 untreated eligibility criteria in addition to separate periods where they were treated with FP/SAL and met the Step 4 treated eligibility criteria (Figure 3). If a person is included in both the untreated Step 2 and treated Step 4 cohorts, they will be contributing different periods of their person-time to each cohort (pre-FP/SAL treatment for Step 2 vs post-FP/SAL treatment for Step 4), and this will be handled in the analysis by assigning different index dates for Step 2 compared to Step 4.

Step 5

We will combine the CPRD groups obtained in Steps 3 and 4 (n~18,000, see note in Step 4 relating to 90% overlap) and using their baseline characteristics will calculate propensity scores for receiving FP/SAL. The propensity score calculation will be based upon a wide range of covariates (see statistical analysis section for full details). Where a participant is contributing more than one treated time period record to the pool of exposed records (as described in Step 4), baseline characteristics will be updated at the beginning of each treatment-eligible period. Multiple eligible treatment periods from a single person are then included in the propensity score model as if they came from different individuals. The variables selected for the score will then become the basis for propensity score modelling in Objectives 2 and 3.

Step 6

Each untreated patient derived in Step 3 (n~6,000) will be matched 1:1 with the FP/SAL treated patient record from Step 4 with the closest propensity score (~12,000) giving us an analysis population for Objective 1 of ~12,000 patients – double the size of TORCH (Figure 2). For those individuals that have contributed multiple exposed records to the pool of CPRD exposed participants (Figure 3), after one of their exposed records has been propensity-score matched to an unexposed

For peer review only

participant, we will remove all other exposed records for that individual from the remaining pool of CPRD exposed participants. This will mean that an individual can only appear once as an exposed participant in the final propensity-score matched cohort.

We will also apply an alternative additional approach for Objective 1, where instead of generating and using propensity scores to obtain a final analysis population at Steps 5 and 6, we will match records from our exposed TORCH-eligible cohort with participants from the TORCH trial to create a TORCH-analogous exposed patient cohort. This will then be combined with the TORCH-analogous unexposed patient cohort to create a final analysis population (with multivariable regression techniques used to account for confounding instead of propensity scores).

For peer review only

Figure 2: Flowchart illustrating the planned selection of CPRD participants for Objective 1 of the COPD real-world medicines effects study

[FIGURE 2 HERE]

For peer review only

Note in relation to Step 5 (n~18,000) compared to Step 1 (n~13,000): Approximately 90% of the treated patients will also have been eligible as untreated patients, as they did not receive FP/SAL on their TORCH-eligibility date. This means that they will have at least one period of time during which they are untreated-eligible but then did subsequently go-on to receive FP/SAL (meaning they have at least one period of time during which they are treated-eligible). If a person is included as both a treated and untreated participant, they will be contributing different periods of their person-time to each cohort (pre-FP/SAL treatment for the untreated versus post-FP/SAL treatment for treated), and this is handled in the analysis by assigning different index dates

Figure 3: Example timeline for a person in CPRD who is eligible for participation in Objective 1, illustrating multiple periods of unexposed/exposed (FL/SAL untreated/treated) eligibility.

[FIGURE 3 HERE]

1. 1st date of study eligibility: person meets TORCH eligibility as detailed in Objective 1, Step 1 on this date i.e. has a diagnosis of COPD, is between 0-80 years, FEV1<60% predicted and FEV1/FVC ratio <70%, smoking history, no asthma history, no lung surgery history, no long-term O_2 therapy, no alpha-1 antitrypsin deficiency, no drug/alcohol abuse, no exposure to FP/SAL within the previous 4 weeks.
2. Eligible to enter study as an untreated participant: patient can be selected as an untreated participant on any date within this period (as detailed in Step 2).
3. Eligible to enter study as a treated participant: FP/SAL treatment starts, patient is able to be selected as a treated study participant on the date that FP/SAL treatment starts (as detailed in Step 4).
4. Eligible to enter study as an untreated participant: patient stops treatment, but is not immediately eligible for selection again as an untreated study participant. After 4 weeks of no FP/SAL treatment however, they meet the TORCH eligibility ciriteria, and may be selected at any date during the remaining one untreated week as an untreated patient. This is the second untreated period that this person can contribute to the total pool of untreated period records that will be available for matching to the TORCH participants (as detailed in Steps 2 and 3).
5. Eligible to enter study as a treated participant: FP/SAL treatment (re)starts, patient can be selected as a treated study participant on the date that FP/SAL treatment (re)starts. This is the second treated period that this person can contribute to the total pool of treated period records that will be available for propensity score matching to the untreated participants (as detailed in Steps 4 to 6).

Objective 2: Measurement of COPD treatment effects in patients excluded from trials

We will select separate cohorts of patients who have a valid COPD diagnosis in the CPRD[8] and who would not have been eligible for inclusion in the TORCH trial (and therefore also not eligible for our Objective 1) due to the following characteristics: (1) age >80 years OR (2) history of lung surgery OR (3) history of long term oxygen therapy OR (4) evidence of drug/alcohol abuse OR (4) an asthma diagnosis at any time prior to inclusion OR (5) substantial comorbidity. In relation to substantial comorbidity, TORCH required people to be excluded from the trial if they had serious uncontrolled disease with a likelihood of causing death within 3 years. It is likely this criterion affected participant selection and led to a lower overall rate of death than originally anticipated, although we recognise this criterion is subjective. During Objective 1 we will be able to select groups of people who were generally not included despite being eligible, most likely because of this subjective exclusion criterion. We anticipate this will be people with substantial comorbidity e.g. serious vascular disease. Status for such diseases is readily identified in both the CPRD data and in the TORCH baseline data. We will only be able to specify this criterion in detail after we have completed Objective 1.

Participants for each of the Objective 2 cohorts will be selected in a similar fashion to the Objective 1 cohort, with the amended eligibility criteria specified above applied (i.e. Step 1 will be modified for selection of each of the Objective 2 cohorts). As for Objective 1, each participant will be allowed to

For peer review only

have multiple FP/SAL exposed and unexposed eligibility periods in their record, as described in Figure 3. In contrast to Objective 1, there will be no matching of unexposed patients to TORCH patients, as we do not require a TORCH-analagous cohort for this analysis (i.e. no Step 3). All other selection steps will be as applied for Objective 1, including the use of propensity score matching in order to obtain comparable unexposed and exposed groups for analysis.

Objective 3: Determination of treatment effects in an under-studied disease stage

We will select separate cohorts of patients who have a valid COPD diagnosis in the CPRD[8] and who would not have been eligible for inclusion in the TORCH trial (or our Objective 1) due to the following characteristics: (1) >60% predicted FEV1 (or >50% plus MRC breathlessness scale 1 or 2, or >50% plus COPD Assessment Test (CAT) score <10) AND (2) no exacerbations in the year post COPD diagnosis. We will also perform a sensitivity analysis where we allow the group of people with FEV1 >60% predicted who had a maximum of one exacerbation within 1 year post COPD diagnosis to be included. As for Objective 2, the selection steps will be similar to Objective 1, with modified criteria for step 1 and the removal of the TORCH-matching step (step 3).

Exposures, outcomes and co-variates

Exposures

For all objectives, exposures will be determined using CPRD prescribing records and code lists for COPD treatments (codelists provided in supplementary materials).

For Objective 1, use of FP/SAL (tradename Seretide) is the primary exposure of interest and will be compared with no treatment with FP/SAL. We will limit included patients to those receiving Seretide 500/50, the dose used in TORCH. This information is recorded for all prescriptions of Seretide and this dose is the only currently approved dose for COPD in the UK (though we recognise some prescribing may not follow the licensed indication). If the results for our FP/SAL vs no treatment comparison are not consistent with the TORCH FP/SAL vs placebo results (see Statistical analysis – Validation of results against TORCH section for a definition of consistent), we will perform additional analyses where instead of using a no-treatment comparator group, our Objective 1 comparator group will be people exposed to Salmeterol, one of the other comparator groups from the TORCH trial.

As a secondary analysis in Objective 1 other treatments for COPD will also be compared with no treatment. Selection of unexposed and exposed people for each of these drugs will follow Steps 1 – 6 detailed above in the Selection of Participants – Objective 1 section, although Step 3 will be omitted (as these cohorts will not need to be matched to TORCH). The other treatments we plan to include are as follows:

a) long-acting beta agonist (LABA)

For peer review only

b) long-acting muscarinic antagonist (LAMA)
c) LABA + LAMA
d) LABA + inhaled corticosteroid (ICS) other than FP/SAL
e) LABA + LAMA + ICS

For Objectives 2 and 3 we will again use recorded prescribing information to determine the dose recieved. We will be reliant mostly on the strength of each individual drug which is recorded automatically against each product and does not require GPs to enter this data, ensuring completeness. We will then be able to stratify analyses based on the dose prescribed. Specific exposures for Objectives 2 and 3 are as follows (all versus no treatment):

a) FP/SAL
b) LABA
c) LAMA
d) LABA + LAMA
e) LABA + ICS
f) LABA + LAMA + ICS

Outcomes

Outcomes to be measured are as follows:

1. COPD exacerbation*:* to be defined using a CPRD-Hospital Episodes Statistics (HES) algorithm developed previously by authors of this study protocol[10]
2. All cause mortality: as recorded in ONS mortality statistics (data that is linked to CPRD data)
3. Pneumonia: as defined using a CPRD-HES algorithm published previously by authors of this study protocol[11]
4. Time to COPD treatment change: determined by prescribing records indicating the start of a new, additional COPD treatment

Covariates

Covariates to be considered for inclusion in the propensity score include the following (all obtained from CPRD data):

- Lung function (FEV1, FEV1/FVC)
- Age
- Gender
- Body mass index
- Alcohol consumption
- Vascular disease (broken into individual components e.g. hypertension, heart failure, atherosclerotic disease)
- Use of prescribed aspirin and statins
- Prior treatment with other COPD medication
- Type 2 diabetes
- History of cancer
- Renal disease
- Healthcare utilisation intensity (number of prior visits, hospitalisations, number of distinct medications used, number of procedures)

For peer review only

Sample size

Objective 1

Assuming a baseline conservative exacerbation rate of 0.5 per patient per year,[10] we would only require a sample of 408 patients per treatment group to detect a reduction in annual exacerbation rate to 0.4 per year, with 80% power and 5% significance. The estimated sample size is ~12,000 which will provide ample power for the main outcomes of interest, but also allow stratification by patient characteristics to determine stratified results, and will also be ample for the secondary analyses where we will use 99% confidence intervals. For example, to detect a reduction from 0.5 to 0.4 exacerbations per year with 80% power and 1% significance we would need ~600 people in each treatment group.

Objectives 2 and 3

We are also confident that we will have sufficient numbers to allow well powered analyses for Objectives 2 and 3. For example a feasibility count looking at the number of people over the age of 80 eligible for inclusion in Objective 2 estimated that there would be >2,000 people in each exposure group.

Statistical analysis

Propensity score for addressing confounding

The propensity score will be constructed using the principle that predictors of the exposure and outcome, or outcome only (mortality) should be included. We will consider a wide range of factors for inclusion (as listed in the covariates section above), such as: age, sex, body mass index, alcohol consumption, and a wide range of comorbidities (e.g. type 2 diabetes, coronary heart disease, cerebrovascular disease, peripheral vascular disease, heart failure, hypertension, renal disease, cancer). We will further adjust for healthcare utilisation intensity (number of prior visits, hospitalisations, number of distinct medications used, number of procedures, etc.) as these are generic correlates of disease state and the likelihood of recording completeness. We have substantial prior experience of building propensity models[13–15,16].

For the additional alternative approach to Objective 1 relying on matching of both unexposed and exposed patients to the TORCH trial patients (described in Selection of participants – Objective 1 – Step 6), we will use multivariable regression techniques to address confounding, considering a similar wide range of covariates for adjustment.

The variable list used for the propensity score model obtained in Objective 1 will be the basis for propensity score modelling in Objectives 2 and 3, but additional variables will also be considered given the different nature of the patient populations being studied in these Objectives.

For peer review only

Methods of analysis

For all objectives, comparisons will be made according to FP/SAL (or other drugs being analysed as specified in the Exposure section) status for rate of COPD exacerbation, pneumonia and mortality over 3 years. All analyses will be performed according to the “intention-to-treat” principle (as was done in the TORCH study), meaning that if a participant enters the study as either an exposed or unexposed participant, they will remain assigned to that exposure category for the entire duration of their follow-up (irrespective as to whether their true exposure status changes). For exacerbations, a negative binomial model will be used, accounting for variability between patients in the number and frequency of exacerbations, with the number of exacerbations as the outcome and the log of treated time as an offset variable. Time to mortality, first pneumonia and treatment change will be analysed using Cox proportional hazards regression. This mirrors TORCH endpoints of major benefit and harm. We anticipate the propensity matching process will allow us to assemble treated and untreated groups that are very similar with respect to baseline characteristics except FP/SAL treatment status. However, this will be tested by assessing standardised differences for each baseline variable. If substantial differences are noted for important variables, it may be necessary to further adjust the statistical models. This could also include examining the effect of using greedy vs optimum matching approaches in order to obtain the closest propensity score match, and/or matching at a ratio other than 1:1.[17]

Validation of results against TORCH

We will validate our findings against TORCH as part of Objective 1 by determining whether results of the CPRD FP/SAL versus no FP/SAL treatment analysis are compatible with the TORCH exacerbations rate ratio for FP/SAL versus placebo (0.75; 95% CI 0.69-0.81). This outcome has been selected as it is an outcome of key significance for people with COPD[4] and the result in TORCH shows a clear benefit with 95% confidence limits below 1. We have set two criteria that must be met for us to conclude results are consistent. First the effect size must be clinically comparable with TORCH findings; the rate ratio for exacerbations in CPRD must be between 0.65 and 0.9. This range is deliberately not symmetrical around the TORCH estimate of 0.75 as we would anticipate the treatment effect in routine clinical care may be weaker than that seen in the optimised setting of a randomised trial. We recognise this rule could be met with a poorly powered, inconclusive result, so a second criterion is that the 95% confidence interval for the rate ratio must exclude 1. If we go on to compare FP/SAL with Salmeterol alone (see Exposures, outcomes and co-variates section, Exposures subheading), the 95% confidence interval would also need to exclude 1, and the rate ratio would need to be between 0.81 and 0.95 (compared with the TORCH FP/SAL versus Salmeterol result of 0.88, 95% CI 0.81-0.95).

For peer review only

Handling measurement of adherence to medication

Adherence to issued prescribing in general practice is likely to vary according to the treatment issued e.g. short course antibiotic treatment is notoriously not well adhered to, whereas long term life-saving treatment such as antiretroviral medication is more likely to be taken as prescribed. Whilst we do not have figures for adherence for COPD medication in UK general practice, we are able to estimate the proportion of time covered by prescribing as a proxy for adherence and will account for this in our analyses. Moreover, our intention is to estimate the effect of prescribing at the population level, and to some extent, the clinical effects we will measure are in part due to pharmacological effects, and in part the way the treatment is taken which includes adherence. Also of note, prescribing for COPD in the UK is predominantly through GPs and so we will not be missing prescribing information from other potential sources of treatment.

The data analysis for adherence will necessarily be a significant element of the work to be done for this study. However, we have reviewed the records for a random sample of 30 people with COPD starting treatment with FP/SAL to look at adherence patterns over the course of a year. Of the 30 patients, 20 (67%) were still receiving Seretide (FP/SAL) one year after starting treatment. Of the 20 who received Seretide for a full year, 15 (75%) received sufficient prescriptions to suggest at least 50% adherence over the year, and 8 (40%) had sufficient prescriptions to suggest 80% adherence or higher. As expected, this suggests two things: Firstly adherence is likely to be poorer in routine clinical care than in the trial population; in TORCH 80% of participants were estimated to have adherence at 80% or higher. Secondly there is a wide range of adherence in routine care. This will allow us to estimate both the population level effects of treatment as actually used in routine care, but also to estimate the treatment effect in patients with more similar levels of adherence to TORCH participants. Whilst we acknowledge that prescribing can only be a proxy for used medication, we believe it is not an unreasonable assumption that the amount of medication prescribed is correlated with the amount consumed. We plan to assess adherence for the cohort that we select for Objective 1 beyond 1 year and report the findings. In the event that Objective 1 detects a null or poorer treatment effect than anticipated (rate ratio > 0.9), we will conduct a sensitivity analysis restricted to people estimated to be covered by FP/SAL treatment for 80% of their follow up.

Misclassification of (1) drug exposure periods and (2) outcome status

It is possible that an individual may still be exposed to FP/SAL for some time after a prescription has finished, for example if they have medication at home that they haven't used from a previous prescription. This would mean that people may become eligible for inclusion in the unexposed group while they are actually still exposed. If our result differs from the TORCH results (e.g. a rate ratio <0.65 or >0.9), we will conduct a sensitivity analysis in which we include an additional (grace) exposed period equivalent to the length of a single prescription at the end of each actual exposed

For peer review only

period, and only classify individuals as eligible for inclusion as unexposed at the end of this additional period.

Our results could also be impacted by misclassification of outcome, given the routine nature of the data. Our initial approach for detection of COPD exacerbations is to use a validated case definition from previous work that maximises positive predictive value while maintaining a relatively high sensitivity.[12] If our result differs from the TORCH results, we will consider performing a sensitivity analysis in which we assess the impact of applying alternative case definitions for COPD exacerbations (see supplementary materials for an overview of articles relating to the case definitions we plan to utilise, including any validity measurements provided).

Missing data

CPRD data are shown to be almost complete for drug prescribing and mortality (partly through ONS linkage). Smoking history tends to be very well recorded for people with COPD and missingness is likely to be minimal.[10] Information on important comorbidity is also well recorded in CPRD. We will conduct both complete record analyses and use multiple imputation where appropriate assumptions hold, applying findings from methodological work led by one of the study team (EW) into the use of multiple imputation in propensity score modelling.[16]

Ethics and dissemination

Approval by ethics and scientific comittees

Ethical approval for this study has been obtained from the London School of Hygiene & Tropical Medicine Ethics Committee (Ref: 11997).

An application for scientific approval related to use of the CPRD data has been made to the Independent Scientific Advisory Committee (ISAC) of the Medicines and Healthcare Products Regulatory Agency. CPRD data are already approved via a National Research Ethics Committee for purely non-interventional research of this type.

An application for use of the TORCH trial individual patient data was made to the clinicalstudydatarequest.com site, which is checked by the Wellcome Trust and relevant sponsors to make sure information is complete and that the sponsor's requirements for informed consent have been met. The application is then sent to an independent review panel that consider the scientific rationale, objectives, publication plan, conflicts of interest and qualifications and experience of the research team before making a decision on providing access to the data. We recently obtained approval of all aspects of this application.

For peer review only

Dissemination plans

Dissemination of findings will be via a combination of channels. The work will be published in high ranking peer reviewed journals and we anticipate 3 publications to arise directly from the planned work. Findings will also be presented at relevant scientific conferences such as the British Thoracic Society Conference and the European Respiratory Society International Congress. We will also engage with patients already identified from a clinic run by one of the authors of this protocol (JQ) and from Breathe Easy Groups and with relevant charities such as the British Lung Foundation to determine the most relevant ways to disseminate results directly to patients in an accessible manner, and to help our understanding of the likely impact of results to specific groups of patients. We will communicate directly with NICE to ensure they are kept informed of results that are of direct relevance to the guidance they have issued on COPD, and with the Medicines and Healthcare Products Regulatory Agency if it appears that findings may impact the risk/benefit profile of COPD treatments.

For peer review only

References

1. Comission H. Cleaning the air: a national study of chronic pulmonary disease. 2006.

2. Calverley PMA, Anderson JA, Celli B, Ferguson GT, Jenkins C, Jones PW, et al. Salmeterol and Fluticasone Propionate and Survival in Chronic Obstructive Pulmonary Disease. N Engl J Med. 2007 Feb 22;356(8):775–89.

3. Tashkin DP, Celli B, Senn S, Burkhart D, Kesten S, Menjoge S, et al. A 4-Year Trial of Tiotropium in Chronic Obstructive Pulmonary Disease. N Engl J Med. 2008 Oct 9;359(15):1543–54.

4. National Institute for Health and Care Excellence (NICE). Chronic obstructive pulmonary disease in over 16s: diagnosis and management | Guidance and guidelines | NICE. 2010;

5. Ferguson GT, Anzueto A, Fei R, Emmett A, Knobil K, Kalberg C, et al. Maintenance pharmacotherapy of mild and moderate COPD: what is the evidence? Respir Med. 2011 Sep;105(9):1268–74.

6. EU. REGULATION (EU) No 1235/2010 OF THE EUROPEAN PARLIAMENT AND OF THE COUNCIL. 2010.

7. Spring S. Guidance for Industry Postmarketing Studies and Clinical Trials — Implementation of Section 505(o)(3) of the Federal Food, Drug, and Cosmetic Act.

8. Herrett E, Gallagher AM, Bhaskaran K, Forbes H, Mathur R, Staa T van, et al. Data Resource Profile: Clinical Practice Research Datalink (CPRD). Int J Epidemiol. 2015 Jun;44(3):827–36.

9. Herrett E, Thomas SL, Schoonen WM, Smeeth L, Hall AJ. Validation and validity of diagnoses in the General Practice Research Database: a systematic review. Br J Clin Pharmacol. 2010 Jan;69(1):4–14.

10. Quint JK, Müllerova H, DiSantostefano RL, Forbes H, Eaton S, Hurst JR, et al. Validation of chronic obstructive pulmonary disease recording in the Clinical Practice Research Datalink (CPRD-GOLD). BMJ Open. 2014;4(7):e005540.

11. Millett ERC, Quint JK, Smeeth L, Daniel RM, Thomas SL. Incidence of Community-Acquired Lower Respiratory Tract Infections and Pneumonia among Older Adults in the United Kingdom: A Population-Based Study. Heimesaat MM, editor. PLoS One. 2013 Sep 11;8(9):e75131.

12. Rothnie KJ, Müllerová H, Hurst JR, Smeeth L, Davis K, Thomas SL, et al. Validation of the Recording of Acute Exacerbations of COPD in UK Primary Care Electronic Healthcare Records. Chotirmall SH, editor. PLoS One. 2016 Mar 9;11(3):e0151357.

For peer review only

13. Douglas I, Evans S, Smeeth L. Effect of statin treatment on short term mortality after pneumonia episode: cohort study. BMJ. 2011 Apr 6;342:d1642.

14. Williamson E, Morley R, Lucas A, Carpenter J. Propensity scores: From naïve enthusiasm to intuitive understanding. Stat Methods Med Res. 2012 Jun 24;21(3):273–93.

15. Root AA, Wong AYS, Ghebremichael-Weldeselassie Y, Smeeth L, Bhaskaran K, Evans SJW, et al. Evaluation of the risk of cardiovascular events with clarithromycin using both propensity score and self-controlled study designs. Br J Clin Pharmacol. 2016 Aug;82(2):512–21.

16. Leyrat C, Seaman SR, White IR, Douglas I, Smeeth L, Kim J, et al. Propensity score analysis with partially observed covariates: How should multiple imputation be used? Stat Methods Med Res. 2017 Jun 2;96228021771303.

17. Austin PC. An Introduction to Propensity Score Methods for Reducing the Effects of Confounding in Observational Studies. Multivariate Behav Res. 2011 May;46(3):399–424.

Authors contributions

KW, EW, JC, LW, SS, LS, JQ, ID contributed to study question and design. KW wrote the first draft of the protocol manuscript (based upon original grant/scientific approval applications to NIHR and ISAC that KW, EW, JC, LW, SS, LS, JQ and ID all contributed to). KW, EW, JC, LW, SS, LS, JQ, ID contributed to further drafts and approved the final version.

Acknowledgements

No information for this section.

Data sharing statement

There are currently no unpublished data from this study, as it is a protocol.

All of the data sources described can be accessed by making formal applications to the owners of the data (i.e. CPRD/HES data for the routinely collected non-interventional data and clinicalstudyrequest.com for the trial data used for validation of non-interventional methods).

For peer review only

Figure legends

Figure 1: Overview of study objectives and sources of data for the COPD real-world medicines effects study (RCT=randomised controlled trial, CPRD= the UK Clinical Practice Research Datalink, FP/SAL=Fluticasone propionate + salmeterol)

Figure 2: Flowchart illustrating the planned selection of CPRD participants for Objective 1 of the COPD real-world medicines effects study

Figure 3: Example timeline for a person in CPRD who is eligible for participation in Objective 1, illustrating multiple periods of unexposed/exposed (FL/SAL untreated/treated) eligibility.

For peer review only

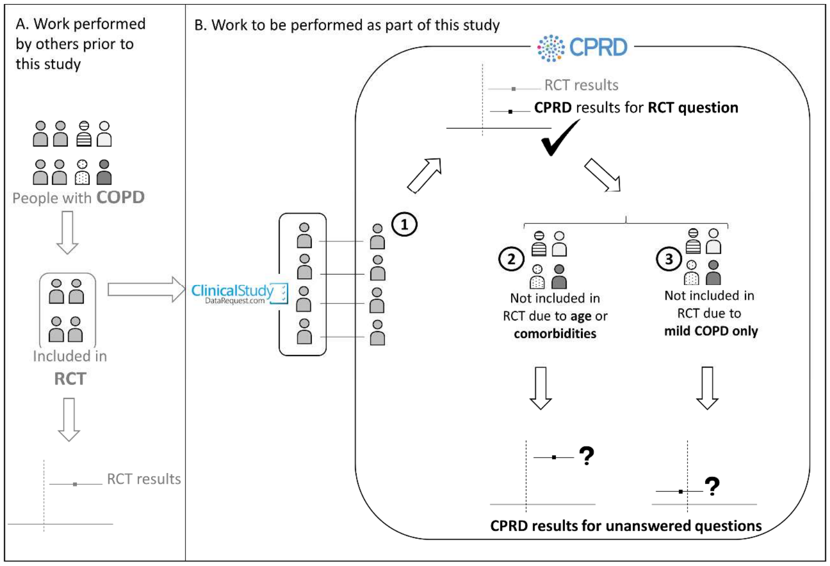

Figure 1: Overview of study objectives and sources of data for the COPD real-world medicines effects study (RCT=randomised controlled trial, CPRD= the UK Clinical Practice Research Datalink, FP/SAL=Fluticasone propionate + salmeterol)

338x190mm (300 x 300 DPI)

FIGURE 2

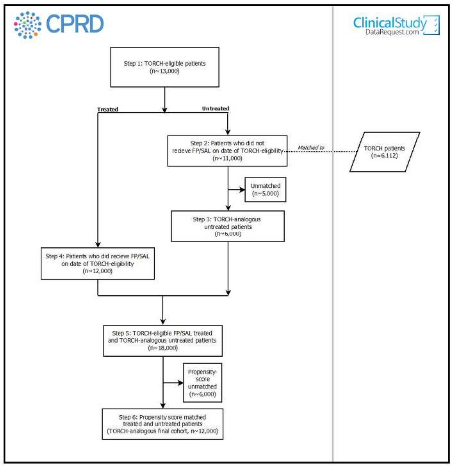

Figure 2: Flowchart illustrating the planned selection of CPRD participants for Objective 1 of the COPD real-world medicines effects study

338x190mm (300 x 300 DPI)

review only

FIGURE 3

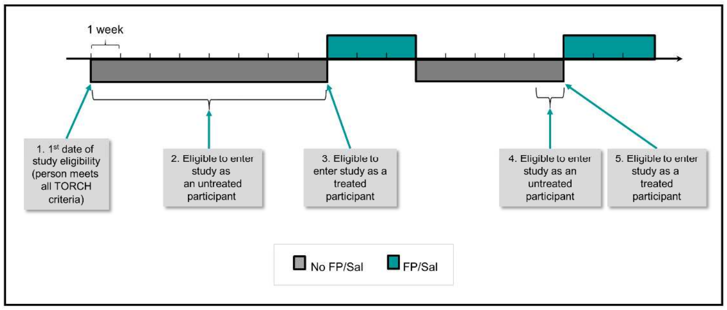

Figure 3: Example timeline for a person in CPRD who is eligible for participation in Objective 1, illustrating multiple periods of unexposed/exposed (FL/SAL untreated/treated) eligibility.

338x190mm (300 x 300 DPI)

review only

Overview of algorithms to be used for detecting COPD, COPD exacerbations and pneumonia

Condition	Paper (author, year)	Algorithm description[1]	Validity[2]	Other notes
COPD	Quint et al, 2014[10]	- CPRD[3] diagnostic (Read) code for COPD	PPV[4]: 87% (78 – 92)	1. Comparison with gold standard of respiratory physician review of information obtained by questionnaire from GPs<br>2. 8 algorithms presented in total, PPVs ranging from 12 to 89
COPD exacerbation	Rothnie et al, 2016[12]	- CPRD diagnostic (Read) code for LRTI or Acute Exacerbation COPD (AECOPD) **OR**<br>- A prescription of a COPD-specific antibiotic combined with OCS for 5-14 days **OR**<br>- A record (Read code) of two or more respiratory symptoms of AECOPD with a prescription of COPD-specific antibiotics and/or OCS on the same day	PPV: 86% (83 – 88)<br>Sensitivity: 63% (55 – 70)	1. Comparison with gold standard of respiratory physician review of information obtained by questionnaire from GPs<br>2. 15 algorithms presented in total, PPVs ranging from 61% – 100%, sensitivities ranging from 1.6% – 63%
Pneumonia	Millet et al, 2013[11]	- CPRD diagnostic (Read) codes and HES[5] diagnostic (ICD-10) codes for pneumonia (identified as a subset of an initial search for LRTI codes)<br>- Records in both database within the 28 days considered the same illness-episode	No validation performed	

Note 1: Main algorithm presented in article and to be applied initially in COPD medications real-world effects study (details on other algorithms presented in paper provided in the “Other notes” column where appropriate). **Note 2**: Validity=measure of validity presented in article:result obtained (95% CI). **Note 3**: CPRD=UK Clinical Practice Research Datalink **Note 4**: PPV=positive predictive value **Note 5:** HES=Hospital Episode Statistics

For peer review only

LAMA codes

Product code	BNF header	Drug substance	Drug product
61176	compound bronchodilator preparations	vilanterol trifenatate/umeclidinium bromide	anoro ellipta 55micrograms/dose / 22micrograms/dose dry powder inhaler (glaxosmithkline uk ltd)
61490	compound bronchodilator preparations		umeclidinium bromide 65micrograms/dose / vilanterol 22micrograms/dose dry powder inhaler
35014	antimuscarinic bronchodilators	tiotropium bromide monohydrate	tiotropium bromide 18microgram inhalation powder capsules with device
6474	antimuscarinics	glycopyrronium bromide	robinul 1mg tablet (idis world medicines)
50577	antimuscarinic bronchodilators	tiotropium bromide	spiriva 18microgram inhalation powder capsules with handihaler (de pharmaceuticals)
35011	antimuscarinic bronchodilators	tiotropium bromide monohydrate	tiotropium bromide 18microgram inhalation powder capsules
49227	antimuscarinic bronchodilators		aclidinium bromide 375micrograms/dose dry powder inhaler
50103	antimuscarinic bronchodilators		spiriva 18microgram inhalation powder capsules with handihaler (waymade healthcare plc)
7908	antimuscarinics	glycopyrronium bromide	robinul 2mg tablet (wyeth pharmaceuticals)
59638	antimuscarinic bronchodilators		spiriva 18microgram inhalation powder capsules with handihaler (sigma pharmaceuticals plc)
51967	antimuscarinic bronchodilators	tiotropium bromide	spiriva 18microgram inhalation powder capsules (mawdsley-brooks & company ltd)
53982	antimuscarinic bronchodilators		seebri breezhaler 44microgram inhalation powder capsules with device (novartis pharmaceuticals uk ltd)
6050	antimuscarinic bronchodilators	tiotropium bromide	spiriva 18 microgram capsule (boehringer ingelheim ltd)
7597	other ulcer healing drugs	glycopyrronium bromide	glycopyrronium bromide 2mg tablets
49228	antimuscarinic bronchodilators		eklira 322micrograms/dose genuair (almirall ltd)
59173	other ulcer healing drugs	glycopyrronium bromide	glycopyrronium bromide 200micrograms/5ml oral suspension

For peer review only

Product code	BNF header	Drug substance	Drug product
36864	antimuscarinic bronchodilators	tiotropium bromide	tiotropium bromide 2.5micrograms/dose solution for inhalation cartridge with device cfc free
55911	other ulcer healing drugs	glycopyrronium bromide	glycopyrronium bromide 500micrograms/5ml oral solution
34995	antimuscarinic bronchodilators	tiotropium bromide	spiriva 18microgram inhalation powder capsules with handihaler (boehringer ingelheim ltd)
35000	antimuscarinic bronchodilators	tiotropium bromide	spiriva 18microgram inhalation powder capsules (boehringer ingelheim ltd)
29138	other ulcer healing drugs	glycopyrronium bromide	glycopyrronium bromide 1mg/5ml oral solution
47269	other ulcer healing drugs	glycopyrronium bromide	glycopyrronium bromide 1mg/5ml oral suspension
54151	other ulcer healing drugs	glycopyrronium bromide	glycopyrronium bromide 600micrograms/5ml oral suspension
55795	other ulcer healing drugs	glycopyrronium bromide	glycopyrronium bromide 500micrograms/5ml oral suspension
38377	other ulcer healing drugs	glycopyrronium bromide	glycopyrronium bromide 2mg/5ml oral solution
7218	other ulcer healing drugs	glycopyrronium bromide	glycopyrronium bromide 1mg tablets
36869	antimuscarinic bronchodilators	tiotropium bromide	spiriva respimat 2.5micrograms/dose solution for inhalation cartridge with device (boehringer ingelheim ltd)
62109	antimuscarinic bronchodilators		umeclidinium bromide 65micrograms/dose dry powder inhaler
50292	antimuscarinic bronchodilators		spiriva 18microgram inhalation powder capsules (sigma pharmaceuticals plc)
55794	other ulcer healing drugs	glycopyrronium bromide	glycopyrronium bromide 5mg/5ml oral suspension
50047	other ulcer healing drugs	glycopyrronium bromide	glycopyrronium bromide 5mg/5ml oral solution
56262	other ulcer healing drugs	glycopyrronium bromide	glycopyrronium bromide 200micrograms/5ml oral solution
61879	antimuscarinic bronchodilators		incruse ellipta 55micrograms/dose dry powder inhaler (glaxosmithkline uk ltd)
53761	antimuscarinic bronchodilators		glycopyrronium bromide 55microgram inhalation powder capsules with device
746	antimuscarinic bronchodilators	tiotropium bromide	tiotropium 18 microgram capsule
38538	other ulcer healing drugs	glycopyrronium bromide	glycopyrronium bromide 2mg/5ml oral suspension
46214	antimuscarinics	glycopyrronium bromide	glycopyrronium bromide 5mg/5ml oral solution

For peer review only

Product code	BNF header	Drug substance	Drug product
61582	antimuscarinic bronchodilators	tiotropium bromide	spiriva respimat 2.5micrograms/dose solution for inhalation cartridge with device (waymade healthcare plc)

LABA codes

Product code	BNF header	Drug substance	Drug product
45610	selective beta 2 agonists	indacaterol maleate	indacaterol 300microgram inhalation powder capsules with device
7270	selective beta 2 agonists	salmeterol xinafoate	salmeterol 25micrograms/dose inhaler cfc free
10968	selective beta 2 agonists	formoterol fumarate dihydrate	foradil 12microgram inhalation powder capsules with device (novartis pharmaceuticals uk ltd)
549	unknown	salmeterol xinafoate	serevent 25micrograms/dose inhaler (glaxosmithkline uk ltd)
7133	selective beta 2 agonists	formoterol fumarate dihydrate	formoterol 12micrograms/dose dry powder inhaler
54742	selective beta 2 agonists	salmeterol xinafoate	salmeterol 25micrograms/dose inhaler cfc free (a a h pharmaceuticals ltd)
719	selective beta 2 agonists	salmeterol xinafoate	salmeterol 50micrograms/dose dry powder inhaler
57694	selective beta 2 agonists	salmeterol xinafoate	vertine 25micrograms/dose inhaler cfc free (teva uk ltd)
26829	selective beta 2 agonists	tulobuterol	brelomax 2mg tablet (abbott laboratories ltd)
19799	selective beta 2 agonists	tulobuterol	tulobuterol 2mg
56482	selective beta 2 agonists	formoterol fumarate dihydrate	oxis 12 turbohaler (waymade healthcare plc)
25784	selective beta 2 agonists	formoterol fumarate dihydrate	atimos modulite 12micrograms/dose inhaler (chiesi ltd)
47638	selective beta 2 agonists	salmeterol xinafoate	neovent 25micrograms/dose inhaler cfc free (kent pharmaceuticals ltd)
10672	peripheral vasodilators and related drugs	moxisylyte hydrochloride	opilon 40mg tablet (concord pharmaceuticals ltd)
50051	selective beta 2 agonists	salmeterol xinafoate	serevent 25micrograms/dose evohaler (waymade healthcare plc)
35725	selective beta 2 agonists	formoterol fumarate dihydrate	formoterol easyhaler 12micrograms/dose dry powder inhaler (orion pharma (uk) ltd)

For peer review only

Product code	BNF header	Drug substance	Drug product
2224	selective beta 2 agonists	salmeterol xinafoate	serevent 50micrograms/dose accuhaler (glaxosmithkline uk ltd)
43893	selective beta 2 agonists	indacaterol maleate	onbrez breezhaler 150microgram inhalation powder capsules with device (novartis pharmaceuticals uk ltd)
6526	selective beta 2 agonists	formoterol fumarate dihydrate	formoterol 12microgram inhalation powder capsules with device
43738	selective beta 2 agonists	indacaterol maleate	indacaterol 150microgram inhalation powder capsules with device
35825	selective beta 2 agonists	salmeterol xinafoate	serevent 50microgram disks (glaxosmithkline uk ltd)
43764	peripheral vasodilators and related drugs	moxisylyte hydrochloride	opilon 40mg tablets (archimedes pharma uk ltd)
56478	selective beta 2 agonists	salmeterol xinafoate	serevent 50micrograms/dose accuhaler (de pharmaceuticals)
42103	selective beta 2 agonists	tulobuterol	tulobuterol 1mg/5ml sugar free syrup
57544	selective beta 2 agonists	salmeterol xinafoate	serevent 50micrograms/dose accuhaler (waymade healthcare plc)
3297	selective beta 2 agonists	salmeterol xinafoate	salmeterol 50micrograms disc
57558	selective beta 2 agonists	formoterol fumarate dihydrate	oxis 6 turbohaler (lexon (uk) ltd)
9711	selective beta 2 agonists	formoterol fumarate dihydrate	formoterol 6micrograms/dose dry powder inhaler
35542	selective beta 2 agonists	salmeterol xinafoate	salmeterol 50microgram inhalation powder blisters with device
465	unknown	salmeterol xinafoate	salmeterol 25micrograms/dose inhaler
44064	selective beta 2 agonists	indacaterol maleate	onbrez breezhaler 300microgram inhalation powder capsules with device (novartis pharmaceuticals uk ltd)
35165	selective beta 2 agonists	salmeterol xinafoate	serevent 50microgram disks with diskhaler (glaxosmithkline uk ltd)
14306	selective beta 2 agonists	formoterol fumarate dihydrate	formoterol 12micrograms/dose inhaler cfc free
1974	selective beta 2 agonists	formoterol fumarate dihydrate	oxis 12 turbohaler (astrazeneca uk ltd)
7268	selective beta 2 agonists	salmeterol xinafoate	serevent 25micrograms/dose evohaler (glaxosmithkline uk ltd)
8365	peripheral vasodilators and related drugs	moxisylyte hydrochloride	moxisylyte 40mg tablets
35503	selective beta 2 agonists	salmeterol xinafoate	salmeterol 50microgram inhalation powder blisters
1975	selective beta 2 agonists	formoterol fumarate dihydrate	oxis 6 turbohaler (astrazeneca uk ltd)
22663	selective beta 2 agonists	tulobuterol	respacal 2mg tablet (ucb pharma ltd)

For peer review only

Product code	BNF header	Drug substance	Drug product
910	selective beta 2 agonists	salmeterol xinafoate	serevent diskhaler 50microgram inhalation powder (glaxo wellcome uk ltd)

For peer review only

ICS codes

Product code	BNF header	Drug substance	Drug product
54399	corticosteroids (for respiratory conditions)	beclometasone dipropionate	qvar 100 autohaler (sigma pharmaceuticals plc)
959	unknown	budesonide	budesonide 50micrograms/dose inhaler
2951	corticosteroids (for respiratory conditions)	fluticasone propionate	fluticasone 250microgram/actuation pressurised inhalation
50129	corticosteroids (for respiratory conditions)	beclometasone dipropionate	qvar 100micrograms/dose easi-breathe inhaler (de pharmaceuticals)
2229	corticosteroids (for respiratory conditions)	beclometasone dipropionate	becodisks 100microgram disc (allen & hanburys ltd)
3989	corticosteroids (for respiratory conditions)	fluticasone propionate	flixotide 100microgram disc (allen & hanburys ltd)
5551	corticosteroids (for respiratory conditions)	fluticasone propionate	flixotide 0.5mg/2ml nebules (glaxosmithkline uk ltd)
34794	corticosteroids (for respiratory conditions)	beclometasone dipropionate	beclometasone 200micrograms/dose inhaler (a a h pharmaceuticals ltd)
41269	corticosteroids (for respiratory conditions)	beclometasone dipropionate	beclometasone 400 cyclocaps (teva uk ltd)
29325	corticosteroids (for respiratory conditions)	beclometasone dipropionate	beclometasone 250micrograms/dose inhaler (generics (uk) ltd)
4499	corticosteroids (for respiratory conditions)	beclometasone dipropionate	aerobec 250microgram/actuation pressurised inhalation (meda pharmaceuticals ltd)
57589	corticosteroids (for respiratory conditions)	beclometasone dipropionate	becloforte 250micrograms/dose inhaler (dowelhurst ltd)
32874	corticosteroids (for respiratory conditions)	beclometasone dipropionate	beclometasone 50microgram/actuation inhalation powder (actavis uk ltd)
2159	corticosteroids (for respiratory conditions)	beclometasone dipropionate	aerobec 50 autohaler (meda pharmaceuticals ltd)
14590	corticosteroids (for respiratory conditions)	beclometasone dipropionate	asmabec 250microgram/actuation spacehaler (celltech pharma europe ltd)
1725	corticosteroids (for respiratory conditions)	beclometasone dipropionate	beclazone 50 easi-breathe inhaler (teva uk ltd)
908	corticosteroids (for respiratory conditions)	budesonide	pulmicort 400 turbohaler (astrazeneca uk ltd)
35288	corticosteroids (for respiratory conditions)	beclometasone dipropionate	beclometasone 400microgram inhalation powder blisters
49367	corticosteroids (for respiratory conditions)	beclometasone dipropionate	clenil modulite 50micrograms/dose inhaler (mawdsley-brooks & company ltd)
35107	corticosteroids (for respiratory conditions)	beclometasone dipropionate	beclometasone 400microgram inhalation powder blisters with device

For peer review only

Product code	BNF header	Drug substance	Drug product
1676	corticosteroids (for respiratory conditions)	fluticasone propionate	flixotide 125microgram/actuation inhalation powder (allen & hanburys ltd)
49772	corticosteroids (for respiratory conditions)	fluticasone propionate	fluticasone 250micrograms/dose evohaler (sigma pharmaceuticals plc)
48088	unknown	budesonide	budenofalk 9mg gastro-resistant granules sachets (dr. falk pharma uk ltd)
33258	corticosteroids (for respiratory conditions)	beclometasone dipropionate	beclometasone 250micrograms/dose inhaler (a a h pharmaceuticals ltd)
13037	corticosteroids (for respiratory conditions)	beclometasone dipropionate	pulvinal beclometasone dipropionate 200micrograms/dose dry powder inhaler (chiesi ltd)
19031	corticosteroids (for respiratory conditions)	beclometasone dipropionate	bdp 100microgram/actuation spacehaler (celltech pharma europe ltd)
16158	corticosteroids (for respiratory conditions)	beclometasone dipropionate	clenil modulite 50micrograms/dose inhaler (chiesi ltd)
2124	unknown		pulmicort refil 200 mcg inh
4759	corticosteroids (for respiratory conditions)	beclometasone dipropionate	beclometasone 100microgram inhalation powder capsules
3753	unknown		flixotide diskhaler-community pack 250 mcg
49711	corticosteroids (for respiratory conditions)	budesonide	pulmicort 200micrograms/dose inhaler (astrazeneca uk ltd)
15326	corticosteroids (for respiratory conditions)	beclometasone dipropionate	beclometasone 100micrograms/dose inhaler cfc free
2335	corticosteroids (for respiratory conditions)	beclometasone dipropionate	qvar 100 inhaler (teva uk ltd)
5580	corticosteroids (for respiratory conditions)	fluticasone propionate	flixotide accuhaler 50 50microgram/inhalation inhalation powder (allen & hanburys ltd)
51681	corticosteroids (for respiratory conditions)	beclometasone dipropionate	qvar 100 inhaler (sigma pharmaceuticals plc)
8433	corticosteroids (for respiratory conditions)	budesonide	budesonide 100micrograms/actuation inhaler
9577	corticosteroids (for respiratory conditions)	beclometasone dipropionate	asmabec 50 clickhaler (focus pharmaceuticals ltd)
2723	corticosteroids (for respiratory conditions)	fluticasone propionate	fluticasone 25micrograms/dose inhaler
23675	unknown		pulmicort l.s. refil
35638	corticosteroids (for respiratory conditions)	fluticasone propionate	fluticasone propionate 100microgram inhalation powder blisters with device
9571	corticosteroids (for respiratory conditions)	beclometasone dipropionate	beclometasone 250micrograms/actuation vortex inhaler
51234	corticosteroids (for respiratory conditions)	beclometasone dipropionate	qvar 100 inhaler (waymade healthcare plc)

For peer review only

Product code	BNF header	Drug substance	Drug product
60946	corticosteroids (in chronic bowel disorders)	budesonide	entocort cr 3mg capsules (waymade healthcare plc)
5223	corticosteroids (for respiratory conditions)	fluticasone propionate	fluticasone 50micrograms/dose inhaler cfc free
53057	corticosteroids (for respiratory conditions)	fluticasone propionate	flixotide 50micrograms/dose evohaler (lexon (uk) ltd)
13290	corticosteroids (for respiratory conditions)	beclometasone dipropionate	clenil modulite 100micrograms/dose inhaler (chiesi ltd)
42928	corticosteroids (for respiratory conditions)	fluticasone propionate	flixotide 100micrograms/dose accuhaler (glaxosmithkline uk ltd)
14736	corticosteroids (for respiratory conditions)	beclometasone dipropionate	pulvinal beclometasone dipropionate 400micrograms/dose dry powder inhaler (chiesi ltd)
52806	corticosteroids (for respiratory conditions)	beclometasone dipropionate	qvar 100 autohaler (lexon (uk) ltd)
57525	corticosteroids (for respiratory conditions)	fluticasone propionate	flixotide 250micrograms/dose accuhaler (stephar (u.k.) ltd)
33849	corticosteroids (for respiratory conditions)	beclometasone dipropionate	beclometasone 100microgram/actuation inhalation powder (neo laboratories ltd)
52732	corticosteroids (for respiratory conditions)	budesonide	pulmicort 0.5mg respules (necessity supplies ltd)
35611	corticosteroids (for respiratory conditions)	fluticasone propionate	flixotide 250microgram disks (glaxosmithkline uk ltd)
8111	corticosteroids (for respiratory conditions)	beclometasone dipropionate	becloforte vm 250microgram/actuation vm pack (allen & hanburys ltd)
39879	unknown	budesonide	budesonide 200micrograms/dose inhaler cfc free
1242	corticosteroids (for respiratory conditions)	beclometasone dipropionate	beclometasone 250micrograms/dose inhaler
26665	unknown		pulmicort complete
37447	corticosteroids (for respiratory conditions)	fluticasone propionate	fluticasone propionate 50microgram inhalation powder blisters
5718	corticosteroids (for respiratory conditions)	fluticasone propionate	flixotide 125micrograms/dose evohaler (glaxosmithkline uk ltd)
7653	corticosteroids (for respiratory conditions)	beclometasone dipropionate	beclometasone 400microgram inhalation powder capsules
28640	corticosteroids (for respiratory conditions)	beclometasone dipropionate	beclometasone 100microgram/actuation inhalation powder (actavis uk ltd)
4365	corticosteroids (for respiratory conditions)	beclometasone dipropionate	beclometasone 100micrograms disc
34739	corticosteroids (for respiratory conditions)	beclometasone dipropionate	beclometasone 50micrograms/dose inhaler (teva uk ltd)
8635	corticosteroids (for respiratory conditions)	fluticasone propionate	flixotide 50microgram disc (allen & hanburys ltd)

For peer review only

Product code	BNF header	Drug substance	Drug product
57579	corticosteroids (for respiratory conditions)	fluticasone propionate	flixotide 50micrograms/dose accuhaler (de pharmaceuticals)
4413	corticosteroids (for respiratory conditions)	beclometasone dipropionate	qvar 100 autohaler (teva uk ltd)
37203	corticosteroids (in chronic bowel disorders)	beclometasone dipropionate	beclometasone 5mg gastro-resistant modified-release tablets
4132	corticosteroids (for respiratory conditions)	fluticasone propionate	fluticasone 125microgram/actuation pressurised inhalation
3018	corticosteroids (for respiratory conditions)	beclometasone dipropionate	beclometasone 50micrograms/dose inhaler
9233	corticosteroids (for respiratory conditions)	beclometasone dipropionate	beclometasone 200microgram inhalation powder capsules
2160	corticosteroids (for respiratory conditions)	beclometasone dipropionate	beclometasone 50micrograms/dose breath actuated inhaler
2148	corticosteroids (for respiratory conditions)	beclometasone dipropionate	beclometasone 400microgram disc
34919	corticosteroids (for respiratory conditions)	beclometasone dipropionate	beclometasone 50micrograms/dose inhaler (a a h pharmaceuticals ltd)
2092	corticosteroids (for respiratory conditions)	budesonide	budesonide 200micrograms/dose dry powder inhaler
21005	corticosteroids (for respiratory conditions)	beclometasone dipropionate	beclometasone 250micrograms/dose inhaler cfc free
10090	corticosteroids (for respiratory conditions)	beclometasone dipropionate	beclometasone 50micrograms/actuation extrafine particle cfc free inhaler
1551	corticosteroids (for respiratory conditions)	beclometasone dipropionate	beclazone 250 inhaler (teva uk ltd)
39067	corticosteroids (in chronic bowel disorders)	beclometasone dipropionate	clipper 5mg gastro-resistant modified-release tablets (chiesi ltd)
51415	corticosteroids (for respiratory conditions)	beclometasone dipropionate	qvar 50 inhaler (mawdsley-brooks & company ltd)
35106	corticosteroids (for respiratory conditions)	beclometasone dipropionate	becodisks 100microgram with diskhaler (glaxosmithkline uk ltd)
35299	corticosteroids (for respiratory conditions)	beclometasone dipropionate	becodisks 400microgram (glaxosmithkline uk ltd)
54207	corticosteroids (for respiratory conditions)	beclometasone dipropionate	qvar 50 inhaler (de pharmaceuticals)
3927	corticosteroids (for respiratory conditions)	beclometasone dipropionate	filair 100 inhaler (meda pharmaceuticals ltd)
17670	corticosteroids (for respiratory conditions)	budesonide	easyhaler budesonide 100micrograms/dose dry powder inhaler (orion pharma (uk) ltd)
883	corticosteroids (for respiratory conditions)	beclometasone dipropionate	becodisks 200microgram disc (allen & hanburys ltd)
8450	unknown		flixotide diskhaler-community pack 50 mcg
3289	corticosteroids (for respiratory conditions)	fluticasone propionate	flixotide 25micrograms/dose inhaler (glaxosmithkline uk ltd)

For peer review only

Product code	BNF header	Drug substance	Drug product
53480	corticosteroids (for respiratory conditions)	beclometasone dipropionate	qvar 100 autohaler (stephar (u.k.) ltd)
39102	unknown	budesonide	budesonide 100micrograms/dose inhaler cfc free
20763	unknown		becloforte
35374	corticosteroids (for respiratory conditions)	fluticasone propionate	flixotide 500microgram disks (glaxosmithkline uk ltd)
39200	corticosteroids (for respiratory conditions)	beclometasone dipropionate	aerobec forte 250 autohaler (meda pharmaceuticals ltd)
9599	corticosteroids (for respiratory conditions)	beclometasone dipropionate	beclazone 50microgram/actuation inhalation powder (actavis uk ltd)
5804	corticosteroids (for respiratory conditions)	beclometasone dipropionate	beclometasone 250micrograms/dose dry powder inhaler
47943	corticosteroids (for respiratory conditions)	beclometasone dipropionate	beclazone easi-breathe (roi) 100microgram/actuation pressurised inhalation (ivax pharmaceuticals ireland)
5683	corticosteroids (for respiratory conditions)	fluticasone propionate	flixotide 250micrograms/dose evohaler (glaxosmithkline uk ltd)
48340	corticosteroids (for respiratory conditions)	beclometasone dipropionate	clenil modulite 100micrograms/dose inhaler (mawdsley-brooks & company ltd)
1885	corticosteroids (for respiratory conditions)	beclometasone dipropionate	beclazone 200 inhaler (teva uk ltd)
911	corticosteroids (for respiratory conditions)	fluticasone propionate	flixotide accuhaler 250 250microgram/inhalation inhalation powder (allen & hanburys ltd)
6095	corticosteroids (in chronic bowel disorders)	budesonide	budesonide 3mg gastro-resistant capsules
28073	corticosteroids (for respiratory conditions)	beclometasone dipropionate	beclometasone 250microgram/actuation pressurised inhalation (approved prescription services ltd)
4131	corticosteroids (for respiratory conditions)	fluticasone propionate	fluticasone 100microgram disc
43074	corticosteroids (for respiratory conditions)	fluticasone propionate	flixotide 500micrograms/dose accuhaler (glaxosmithkline uk ltd)
35905	corticosteroids (for respiratory conditions)	fluticasone propionate	fluticasone propionate 250microgram inhalation powder blisters
27583	unknown		pulmicort
17654	corticosteroids (for respiratory conditions)	beclometasone dipropionate	easyhaler beclometasone 200micrograms/dose dry powder inhaler (orion pharma (uk) ltd)
4545	corticosteroids (for respiratory conditions)	budesonide	pulmicort ls 50microgram refill canister (astrazeneca uk ltd)
1412	corticosteroids (for respiratory conditions)	fluticasone propionate	flixotide 250microgram/actuation inhalation powder (allen & hanburys ltd)

For peer review only

Product code	BNF header	Drug substance	Drug product
3363	corticosteroids (for respiratory conditions)	beclometasone dipropionate	becloforte 400microgram disks with diskhaler (glaxosmithkline uk ltd)
36462	corticosteroids (for respiratory conditions)	fluticasone propionate	fluticasone propionate 500microgram inhalation powder blisters
56471	corticosteroids (for respiratory conditions)	beclometasone dipropionate	becodisks 200microgram (mawdsley-brooks & company ltd)
14294	corticosteroids (for respiratory conditions)	beclometasone dipropionate	qvar 50micrograms/dose easi-breathe inhaler (teva uk ltd)
3570	corticosteroids (for respiratory conditions)	budesonide	budesonide 200micrograms/actuation refill canister
9164	corticosteroids (for respiratory conditions)	fluticasone propionate	fluticasone propionate 50micrograms/dose dry powder inhaler
27188	corticosteroids (for respiratory conditions)	budesonide	easyhaler budesonide 200micrograms/dose dry powder inhaler (orion pharma (uk) ltd)
39099	unknown	budesonide	pulmicort 100micrograms/dose inhaler cfc free (astrazeneca uk ltd)
4926	corticosteroids (for respiratory conditions)	fluticasone propionate	flixotide accuhaler 100 100microgram/inhalation inhalation powder (allen & hanburys ltd)
1642	corticosteroids (for respiratory conditions)	budesonide	budesonide 400micrograms/dose dry powder inhaler
34859	corticosteroids (for respiratory conditions)	beclometasone dipropionate	beclometasone 250microgram/actuation inhalation powder (neo laboratories ltd)
16054	corticosteroids (for respiratory conditions)	budesonide	budesonide 200micrograms/actuation breath actuated powder inhaler
960	corticosteroids (for respiratory conditions)	budesonide	pulmicort 100 turbohaler (astrazeneca uk ltd)
51480	corticosteroids (for respiratory conditions)	beclometasone dipropionate	qvar 100 autohaler (de pharmaceuticals)
7891	corticosteroids (for respiratory conditions)	fluticasone propionate	fluticasone 500microgram disc
56462	corticosteroids (for respiratory conditions)	beclometasone dipropionate	becodisks 400microgram (waymade healthcare plc)
7638	corticosteroids (for respiratory conditions)	fluticasone propionate	fluticasone 250microgram disc
15706	corticosteroids (for respiratory conditions)	beclometasone dipropionate	beclometasone 100 micrograms/actuation vortex inhaler
27915	unknown		fluticasone prop disk refill
2893	corticosteroids (for respiratory conditions)	beclometasone dipropionate	beclometasone 200micrograms disc
5885	corticosteroids (for respiratory conditions)	fluticasone propionate	fluticasone propionate 100micrograms/dose dry powder inhaler

For peer review only

Product code	BNF header	Drug substance	Drug product
3898	corticosteroids (in chronic bowel disorders)	budesonide	budesonide 3mg gastro-resistant modified-release capsules
3546	corticosteroids (for respiratory conditions)	beclometasone dipropionate	qvar 50 inhaler (teva uk ltd)
30210	corticosteroids (for respiratory conditions)	beclometasone dipropionate	beclometasone 250micrograms/dose inhaler (teva uk ltd)
1552	corticosteroids (for respiratory conditions)	beclometasone dipropionate	becloforte easi-breathe 250microgram/actuation pressurised inhalation (allen & hanburys ltd)
5822	corticosteroids (for respiratory conditions)	fluticasone propionate	fluticasone 250micrograms/dose inhaler cfc free
35430	corticosteroids (for respiratory conditions)	beclometasone dipropionate	becodisks 200microgram with diskhaler (glaxosmithkline uk ltd)
42994	corticosteroids (for respiratory conditions)	fluticasone propionate	flixotide 250micrograms/dose accuhaler (glaxosmithkline uk ltd)
36290	corticosteroids (for respiratory conditions)	fluticasone propionate	flixotide 50microgram disks with diskhaler (glaxosmithkline uk ltd)
24898	corticosteroids (for respiratory conditions)	beclometasone dipropionate	spacehaler bdp 100microgram/actuation spacehaler (celltech pharma europe ltd)
35700	corticosteroids (for respiratory conditions)	fluticasone propionate	fluticasone propionate 500microgram inhalation powder blisters with device
3188	unknown		pulmicort complete 50 mcg inh
7788	corticosteroids (for respiratory conditions)	budesonide	budesonide 100micrograms/dose dry powder inhaler
35293	corticosteroids (for respiratory conditions)	beclometasone dipropionate	beclometasone 200microgram inhalation powder blisters with device
8251	unknown		pulmicort refil 50 mg inh
1259	corticosteroids (for respiratory conditions)	beclometasone dipropionate	beclometasone 200micrograms/dose inhaler
16525	corticosteroids (in chronic bowel disorders)	budesonide	budenofalk 3mg gastro-resistant capsules (dr. falk pharma uk ltd)
1680	corticosteroids (for respiratory conditions)	budesonide	pulmicort ls 50micrograms/dose inhaler (astrazeneca uk ltd)
10254	corticosteroids (for respiratory conditions)	mometasone furoate	mometasone 400micrograms/dose dry powder inhaler
20812	unknown		pulmicort refill
14524	corticosteroids (for respiratory conditions)	beclometasone dipropionate	bdp 250microgram/actuation spacehaler (celltech pharma europe ltd)
2992	corticosteroids (for respiratory conditions)	beclometasone dipropionate	beclazone 50 inhaler (teva uk ltd)
7602	corticosteroids (for respiratory conditions)	fluticasone propionate	fluticasone 50microgram disc

For peer review only

Product code	BNF header	Drug substance	Drug product
16305	corticosteroids (for respiratory conditions)	fluticasone propionate	flixotide 2mg/2ml nebules (glaxosmithkline uk ltd)
14757	corticosteroids (for respiratory conditions)	beclometasone dipropionate	pulvinal beclometasone dipropionate 100micrograms/dose dry powder inhaler (chiesi ltd)
909	unknown	budesonide	budesonide 200micrograms/dose inhaler
1959	corticosteroids (for respiratory conditions)	budesonide	pulmicort 0.5mg respules (astrazeneca uk ltd)
1861	corticosteroids (for respiratory conditions)	beclometasone dipropionate	aerobec 100 autohaler (meda pharmaceuticals ltd)
16018	corticosteroids (for respiratory conditions)	mometasone furoate	mometasone 200micrograms/dose dry powder inhaler
35631	corticosteroids (for respiratory conditions)	budesonide	budelin novolizer 200micrograms/dose inhalation powder (meda pharmaceuticals ltd)
60937	corticosteroids (for respiratory conditions)	budesonide	pulmicort 200 turbohaler (dowelhurst ltd)
50037	corticosteroids (for respiratory conditions)	budesonide	pulmicort 0.5mg respules (waymade healthcare plc)
19389	corticosteroids (for respiratory conditions)	beclometasone dipropionate	asmabec 50microgram/actuation spacehaler (celltech pharma europe ltd)
2282	corticosteroids (for respiratory conditions)	fluticasone propionate	fluticasone propionate 500micrograms/dose dry powder inhaler
18848	corticosteroids (for respiratory conditions)	beclometasone dipropionate	qvar 100micrograms/dose easi-breathe inhaler (teva uk ltd)
2440	corticosteroids (for respiratory conditions)	fluticasone propionate	flixotide accuhaler 500 500microgram/inhalation inhalation powder (allen & hanburys ltd)
23741	corticosteroids (for respiratory conditions)	budesonide	novolizer budesonide 200microgram/actuation pressurised inhalation (meda pharmaceuticals ltd)
3988	unknown		flixotide diskhaler-community pack 100 mcg
35724	corticosteroids (for respiratory conditions)	budesonide	budelin novolizer 200micrograms/dose inhalation powder refill (meda pharmaceuticals ltd)
1380	corticosteroids (in chronic bowel disorders)	budesonide	entocort cr 3mg capsules (astrazeneca uk ltd)
56144	corticosteroids (in chronic bowel disorders)	budesonide	budenofalk 9mg gastro-resistant granules sachets (dr. falk pharma uk ltd)
56475	corticosteroids (for respiratory conditions)	fluticasone propionate	flixotide 50micrograms/dose accuhaler (sigma pharmaceuticals plc)
40057	corticosteroids (for respiratory conditions)	budesonide	pulmicort 200micrograms/dose inhaler cfc free (astrazeneca uk ltd)
1236	corticosteroids (for respiratory conditions)	beclometasone dipropionate	becloforte 250micrograms/dose inhaler (glaxosmithkline uk ltd)

For peer review only

Product code	BNF header	Drug substance	Drug product
11497	corticosteroids (for respiratory conditions)	beclometasone dipropionate	beclometasone 400micrograms/dose dry powder inhaler
35408	corticosteroids (for respiratory conditions)	beclometasone dipropionate	becodisks 100microgram (glaxosmithkline uk ltd)
34315	corticosteroids (for respiratory conditions)	beclometasone dipropionate	beclometasone 250microgram/actuation inhalation powder (actavis uk ltd)
956	corticosteroids (for respiratory conditions)	budesonide	pulmicort 200 turbohaler (astrazeneca uk ltd)
18394	corticosteroids (for respiratory conditions)	beclometasone dipropionate	bdp 50microgram/actuation spacehaler (celltech pharma europe ltd)
14321	corticosteroids (for respiratory conditions)	beclometasone dipropionate	beclometasone 200micrograms/dose inhaler cfc free
11732	corticosteroids (for respiratory conditions)	beclometasone dipropionate	beclometasone 50micrograms/dose breath actuated inhaler cfc free
3993	corticosteroids (for respiratory conditions)	beclometasone dipropionate	filair forte 250micrograms/dose inhaler (meda pharmaceuticals ltd)
35225	corticosteroids (for respiratory conditions)	fluticasone propionate	flixotide 100microgram disks with diskhaler (glaxosmithkline uk ltd)
5521	corticosteroids (for respiratory conditions)	beclometasone dipropionate	beclometasone 200micrograms/dose dry powder inhaler
4601	corticosteroids (for respiratory conditions)	beclometasone dipropionate	asmabec 100 clickhaler (focus pharmaceuticals ltd)
16148	corticosteroids (for respiratory conditions)	beclometasone dipropionate	clenil modulite 250micrograms/dose inhaler (chiesi ltd)
2892	corticosteroids (for respiratory conditions)	beclometasone dipropionate	becloforte 400microgram disks (glaxosmithkline uk ltd)
35071	corticosteroids (for respiratory conditions)	beclometasone dipropionate	becodisks 200microgram (glaxosmithkline uk ltd)
35510	corticosteroids (for respiratory conditions)	budesonide	budesonide 200micrograms/dose dry powder inhalation cartridge with device
14567	corticosteroids (for respiratory conditions)	beclometasone dipropionate	asmabec 250 clickhaler (focus pharmaceuticals ltd)
56498	corticosteroids (for respiratory conditions)	budesonide	pulmicort 200 turbohaler (waymade healthcare plc)
24660	unknown		betamethasone valerate
51997	corticosteroids (in chronic bowel disorders)	budesonide	budesonide 9mg gastro-resistant granules sachets
1734	corticosteroids (for respiratory conditions)	beclometasone dipropionate	beclometasone 100micrograms/dose breath actuated inhaler
36090	corticosteroids (for respiratory conditions)	fluticasone propionate	flixotide 100microgram disks (glaxosmithkline uk ltd)
5975	corticosteroids (for respiratory conditions)	fluticasone propionate	fluticasone 125micrograms/dose inhaler cfc free
19401	corticosteroids (for respiratory conditions)	beclometasone dipropionate	beclometasone 250micrograms/actuation inhaler and compact spacer

For peer review only

Product code	BNF header	Drug substance	Drug product
895	corticosteroids (for respiratory conditions)	beclometasone dipropionate	beclazone 100 easi-breathe inhaler (teva uk ltd)
51815	corticosteroids (for respiratory conditions)	fluticasone propionate	flixotide 250micrograms/dose evohaler (waymade healthcare plc)
36021	corticosteroids (for respiratory conditions)	fluticasone propionate	fluticasone propionate 50microgram inhalation powder blisters with device
11198	corticosteroids (for respiratory conditions)	beclometasone dipropionate	beclometasons 50 micrograms/actuation vortex inhaler
50287	corticosteroids (for respiratory conditions)	beclometasone dipropionate	qvar 100 inhaler (de pharmaceuticals)
34428	corticosteroids (for respiratory conditions)	beclometasone dipropionate	beclometasone 50microgram/actuation inhalation powder (neo laboratories ltd)
26063	corticosteroids (for respiratory conditions)	beclometasone dipropionate	beclometasone 100micrograms/dose inhaler (teva uk ltd)
35602	corticosteroids (for respiratory conditions)	budesonide	budesonide 200micrograms/dose dry powder inhalation cartridge
30238	corticosteroids (for respiratory conditions)	beclometasone dipropionate	beclometasone 50microgram/actuation pressurised inhalation (approved prescription services ltd)
14700	corticosteroids (for respiratory conditions)	budesonide	budesonide 400micrograms/actuation inhaler
38	corticosteroids (for respiratory conditions)	beclometasone dipropionate	beclometasone 100micrograms/dose inhaler
13815	corticosteroids (for respiratory conditions)	beclometasone dipropionate	beclazone 100microgram/actuation inhalation powder (actavis uk ltd)
947	corticosteroids (for respiratory conditions)	budesonide	budesonide 50micrograms/actuation refill canister
10321	unknown	budesonide	budesonide 400microgram inhalation powder capsules
1426	corticosteroids (for respiratory conditions)	fluticasone propionate	flixotide 500microgram disc (allen & hanburys ltd)
3150	corticosteroids (for respiratory conditions)	beclometasone dipropionate	beclometasone 100micrograms/actuation extrafine particle cfc free inhaler
35113	corticosteroids (for respiratory conditions)	beclometasone dipropionate	beclometasone 200microgram inhalation powder blisters
1424	corticosteroids (for respiratory conditions)	fluticasone propionate	flixotide 250microgram disc (allen & hanburys ltd)
4803	corticosteroids (for respiratory conditions)	beclometasone dipropionate	beclazone 250microgram/actuation inhalation powder (actavis uk ltd)
56493	corticosteroids (for respiratory conditions)	beclometasone dipropionate	qvar 50micrograms/dose easi-breathe inhaler (sigma pharmaceuticals plc)
61664	corticosteroids (for respiratory conditions)	beclometasone dipropionate	clenil modulite 250micrograms/dose inhaler (waymade healthcare plc)

For peer review only

Product code	BNF header	Drug substance	Drug product
35772	corticosteroids (for respiratory conditions)	fluticasone propionate	fluticasone propionate 100microgram inhalation powder blisters
3119	corticosteroids (for respiratory conditions)	beclometasone dipropionate	becloforte integra 250microgram/actuation inhaler with compact spacer (glaxo laboratories ltd)
5522	corticosteroids (for respiratory conditions)	beclometasone dipropionate	beclometasone 100micrograms/dose dry powder inhaler
25204	corticosteroids (for respiratory conditions)	beclometasone dipropionate	beclometasone 100micrograms/dose inhaler (a a h pharmaceuticals ltd)
7724	corticosteroids (for respiratory conditions)	betamethasone valerate	betamethasone valerate 100micrograms/actuation inhaler
3743	corticosteroids (for respiratory conditions)	beclometasone dipropionate	filair 50 inhaler (meda pharmaceuticals ltd)
1956	corticosteroids (for respiratory conditions)	budesonide	pulmicort 1mg respules (astrazeneca uk ltd)
56499	corticosteroids (for respiratory conditions)	fluticasone propionate	flixotide 500micrograms/dose accuhaler (waymade healthcare plc)
2125	corticosteroids (for respiratory conditions)	budesonide	pulmicort 200microgram refill canister (astrazeneca uk ltd)
36401	corticosteroids (for respiratory conditions)	fluticasone propionate	fluticasone propionate 250microgram inhalation powder blisters with device
4688	corticosteroids (for respiratory conditions)	fluticasone propionate	fluticasone 50microgram/actuation pressurised inhalation
3065	corticosteroids (for respiratory conditions)	betamethasone valerate	bextasol inhalation powder (allen & hanburys ltd)
35118	corticosteroids (for respiratory conditions)	beclometasone dipropionate	becodisks 400microgram with diskhaler (glaxosmithkline uk ltd)
1518	corticosteroids (for respiratory conditions)	fluticasone propionate	flixotide 50microgram/actuation inhalation powder (allen & hanburys ltd)
5309	corticosteroids (for respiratory conditions)	fluticasone propionate	flixotide 50micrograms/dose evohaler (glaxosmithkline uk ltd)
47225	unknown	budesonide	budesonide 9mg gastro-resistant granules sachets
30649	corticosteroids (for respiratory conditions)	budesonide	easyhaler budesonide 400micrograms/dose dry powder inhaler (orion pharma (uk) ltd)
35580	corticosteroids (for respiratory conditions)	beclometasone dipropionate	beclometasone 100microgram inhalation powder blisters with device
41412	corticosteroids (for respiratory conditions)	beclometasone dipropionate	beclometasone 400micrograms/actuation inhaler
20825	corticosteroids (for respiratory conditions)	beclometasone dipropionate	spacehaler bdp 250microgram/actuation spacehaler (celltech pharma europe ltd)

For peer review only

Product code	BNF header	Drug substance	Drug product
35461	corticosteroids (for respiratory conditions)	fluticasone propionate	flixotide 250microgram disks with diskhaler (glaxosmithkline uk ltd)
42985	corticosteroids (for respiratory conditions)	fluticasone propionate	flixotide 50micrograms/dose accuhaler (glaxosmithkline uk ltd)
9921	corticosteroids (for respiratory conditions)	beclometasone dipropionate	beclometasone 100micrograms/dose breath actuated inhaler cfc free
56474	corticosteroids (for respiratory conditions)	fluticasone propionate	flixotide 125micrograms/dose evohaler (de pharmaceuticals)
35986	corticosteroids (for respiratory conditions)	fluticasone propionate	flixotide 50microgram disks (glaxosmithkline uk ltd)
11149	glucocorticoid therapy	betamethasone	betnelan 500microgram tablets (focus pharmaceuticals ltd)
1243	corticosteroids (for respiratory conditions)	beclometasone dipropionate	beclazone 250 easi-breathe inhaler (teva uk ltd)
31774	corticosteroids (for respiratory conditions)	beclometasone dipropionate	beclometasone 50micrograms/dose inhaler (generics (uk) ltd)
56477	corticosteroids (for respiratory conditions)	fluticasone propionate	flixotide 100micrograms/dose accuhaler (waymade healthcare plc)
2600	corticosteroids (for respiratory conditions)	beclometasone dipropionate	beclometasone 250micrograms/dose breath actuated inhaler
16151	corticosteroids (for respiratory conditions)	beclometasone dipropionate	clenil modulite 200micrograms/dose inhaler (chiesi ltd)
1100	corticosteroids (for respiratory conditions)	beclometasone dipropionate	beclazone 100 inhaler (teva uk ltd)
1951	corticosteroids (for respiratory conditions)	beclometasone dipropionate	becodisks 400microgram disc (allen & hanburys ltd)
7948	corticosteroids (for respiratory conditions)	fluticasone propionate	fluticasone propionate 250micrograms/dose dry powder inhaler
21482	corticosteroids (for respiratory conditions)	beclometasone dipropionate	beclometasone 100micrograms/dose inhaler (generics (uk) ltd)
9477	corticosteroids (for respiratory conditions)	beclometasone dipropionate	asmabec 100microgram/actuation spacehaler (celltech pharma europe ltd)
27679	corticosteroids (for respiratory conditions)	beclometasone dipropionate	beclometasone 100microgram/actuation pressurised inhalation (approved prescription services ltd)
35652	corticosteroids (for respiratory conditions)	beclometasone dipropionate	beclometasone 100microgram inhalation powder blisters
46157	corticosteroids (for respiratory conditions)	beclometasone dipropionate	beclometasone 200 cyclocaps (teva uk ltd)
56484	corticosteroids (for respiratory conditions)	fluticasone propionate	flixotide 250micrograms/dose accuhaler (waymade healthcare plc)
3442	unknown		pulmicort complete 200 mcg inh

For peer review only

Product code	BNF header	Drug substance	Drug product
28761	corticosteroids (for respiratory conditions)	beclometasone dipropionate	spacehaler bdp 50microgram/actuation spacehaler (celltech pharma europe ltd)
5992	corticosteroids (for respiratory conditions)	beclometasone dipropionate	beclometasone 50micrograms/dose dry powder inhaler
454	corticosteroids (for respiratory conditions)	budesonide	pulmicort 200microgram inhaler (astrazeneca uk ltd)
3220	corticosteroids (for respiratory conditions)	beclometasone dipropionate	qvar 50 autohaler (teva uk ltd)
18537	unknown	budesonide	budesonide 200microgram inhalation powder capsules
57555	corticosteroids (for respiratory conditions)	fluticasone propionate	flixotide 125micrograms/dose evohaler (dowelhurst ltd)
48709	corticosteroids (for respiratory conditions)	beclometasone dipropionate	qvar 100micrograms/dose easi-breathe inhaler (sigma pharmaceuticals plc)
16584	corticosteroids (for respiratory conditions)	beclometasone dipropionate	beclometasone 50micrograms/dose inhaler cfc free
35392	corticosteroids (for respiratory conditions)	fluticasone propionate	flixotide 500microgram disks with diskhaler (glaxosmithkline uk ltd)

For peer review only

FP_SAL codes

Product code	BNF header	Drug substance	Drug product
638	Selective Beta 2 Agonists/Corticosteroids (for Respiratory Conditions)	Fluticasone propionate/Salmeterol xinafoate	Seretide 250 Accuhaler (GlaxoSmithKline UK Ltd)
665	Selective Beta 2 Agonists/Corticosteroids (for Respiratory Conditions)	Salmeterol xinafoate/Fluticasone propionate	Seretide 100 Accuhaler (GlaxoSmithKline UK Ltd)
3666	Selective Beta 2 Agonists/Corticosteroids (for Respiratory Conditions)	Salmeterol xinafoate/Fluticasone propionate	Seretide 500 Accuhaler (GlaxoSmithKline UK Ltd)
5143	Selective Beta 2 Agonists/Corticosteroids (for Respiratory Conditions)	Fluticasone propionate/Salmeterol xinafoate	Seretide 50 Evohaler (GlaxoSmithKline UK Ltd)
5161	Selective Beta 2 Agonists/Corticosteroids (for Respiratory Conditions)	Fluticasone propionate/Salmeterol xinafoate	Seretide 125 Evohaler (GlaxoSmithKline UK Ltd)
5172	Selective Beta 2 Agonists/Corticosteroids (for Respiratory Conditions)	Fluticasone propionate/Salmeterol xinafoate	Seretide 250 Evohaler (GlaxoSmithKline UK Ltd)
5558	Selective Beta 2 Agonists/Corticosteroids (for Respiratory Conditions)	Salmeterol Xinafoate/Fluticasone Propionate	Salmeterol 50micrograms with fluticasone 500micrograms CFC free inhaler
5864	Selective Beta 2 Agonists/Corticosteroids (for Respiratory Conditions)	Salmeterol Xinafoate/Fluticasone Propionate	Salmeterol 25micrograms with fluticasone 250micrograms CFC free inhaler
5942	Selective Beta 2 Agonists/Corticosteroids (for Respiratory Conditions)	Salmeterol Xinafoate/Fluticasone Propionate	Salmeterol 50micrograms with fluticasone 250micrograms CFC free inhaler
6569	Selective Beta 2 Agonists/Corticosteroids (for Respiratory Conditions)	Salmeterol Xinafoate/Fluticasone Propionate	Salmeterol 25micrograms with fluticasone 125micrograms CFC free inhaler
6616	Selective Beta 2 Agonists/Corticosteroids (for Respiratory Conditions)	Salmeterol Xinafoate/Fluticasone Propionate	Salmeterol 25micrograms with fluticasone 50micrograms CFC free inhaler
6938	Selective Beta 2 Agonists/Corticosteroids (for Respiratory Conditions)	Salmeterol Xinafoate/Fluticasone Propionate	Salmeterol 50micrograms with fluticasone 100micrograms dry powder inhaler
11410	Selective Beta 2 Agonists/Corticosteroids (for Respiratory Conditions)	Salmeterol xinafoate/Fluticasone propionate	Fluticasone propionate 500micrograms/dose / Salmeterol 50micrograms/dose dry powder inhaler
11588	Selective Beta 2 Agonists/Corticosteroids (for Respiratory Conditions)	Fluticasone propionate/Salmeterol xinafoate	Fluticasone 125micrograms/dose / Salmeterol 25micrograms/dose inhaler CFC free
11618	Selective Beta 2 Agonists/Corticosteroids (for Respiratory Conditions)	Fluticasone propionate/Salmeterol xinafoate	Fluticasone 250micrograms/dose / Salmeterol 25micrograms/dose inhaler CFC free
12994	Selective Beta 2 Agonists/Corticosteroids (for Respiratory Conditions)	Fluticasone propionate/Salmeterol xinafoate	Fluticasone 50micrograms/dose / Salmeterol 25micrograms/dose inhaler CFC free

For peer review only

Product code	BNF header	Drug substance	Drug product
13040	Selective Beta 2 Agonists/Corticosteroids (for Respiratory Conditions)	Fluticasone propionate/Salmeterol xinafoate	Fluticasone propionate 250micrograms/dose / Salmeterol 50micrograms/dose dry powder inhaler
13273	Selective Beta 2 Agonists/Corticosteroids (for Respiratory Conditions)	Salmeterol xinafoate/Fluticasone propionate	Fluticasone propionate 100micrograms/dose / Salmeterol 50micrograms/dose dry powder inhaler
48739	Selective Beta 2 Agonists/Corticosteroids (for Respiratory Conditions)	Fluticasone propionate/Salmeterol xinafoate	Seretide 250 Evohaler (DE Pharmaceuticals)
49000	Selective Beta 2 Agonists/Corticosteroids (for Respiratory Conditions)	Fluticasone propionate/Salmeterol xinafoate	Seretide 250 Evohaler (Waymade Healthcare Plc)
50560	Selective Beta 2 Agonists/Corticosteroids (for Respiratory Conditions)	Fluticasone propionate/Salmeterol xinafoate	Seretide 250 Accuhaler (Sigma Pharmaceuticals Plc)
50689	Selective Beta 2 Agonists/Corticosteroids (for Respiratory Conditions)	Fluticasone propionate/Formoterol fumarate dihydrate	Flutiform 50micrograms/dose / 5micrograms/dose inhaler (Napp Pharmaceuticals Ltd)
50886	Selective Beta 2 Agonists/Corticosteroids (for Respiratory Conditions)	Fluticasone propionate/Salmeterol xinafoate	Seretide 250 Evohaler (Stephar (U.K.) Ltd)
51027	Selective Beta 2 Agonists/Corticosteroids (for Respiratory Conditions)	Fluticasone propionate/Salmeterol xinafoate	Seretide 125 Evohaler (DE Pharmaceuticals)
51151	Selective Beta 2 Agonists/Corticosteroids (for Respiratory Conditions)	Fluticasone propionate/Salmeterol xinafoate	Seretide 125 Evohaler (Lexon (UK) Ltd)
51270	Selective Beta 2 Agonists/Corticosteroids (for Respiratory Conditions)	Fluticasone propionate/Formoterol fumarate dihydrate	Fluticasone 50micrograms/dose / Formoterol 5micrograms/dose inhaler CFC free
51394	Selective Beta 2 Agonists/Corticosteroids (for Respiratory Conditions)	Salmeterol xinafoate/Fluticasone propionate	Seretide 500 Accuhaler (Waymade Healthcare Plc)
51593	Selective Beta 2 Agonists/Corticosteroids (for Respiratory Conditions)	Salmeterol xinafoate/Fluticasone propionate	Seretide 500 Accuhaler (DE Pharmaceuticals)
51861	Selective Beta 2 Agonists/Corticosteroids (for Respiratory Conditions)	Salmeterol xinafoate/Fluticasone propionate	Seretide 500 Accuhaler (Mawdsley-Brooks & Company Ltd)
51909	Selective Beta 2 Agonists/Corticosteroids (for Respiratory Conditions)	Fluticasone propionate/Salmeterol xinafoate	Seretide 250 Evohaler (Necessity Supplies Ltd)
53230	Selective Beta 2 Agonists/Corticosteroids (for Respiratory Conditions)	Fluticasone propionate/Salmeterol xinafoate	Seretide 250 Accuhaler (DE Pharmaceuticals)
53283	Selective Beta 2 Agonists/Corticosteroids (for Respiratory Conditions)	Salmeterol xinafoate/Fluticasone propionate	Seretide 100 Accuhaler (Waymade Healthcare Plc)
55411	Corticosteroids Used In Nasal Allergy/Antihistamines In Nasal Allergy	Fluticasone propionate/Azelastine hydrochloride	Fluticasone propionate 50micrograms/dose / Azelastine 137micrograms/dose nasal spray
55435	Corticosteroids Used In Nasal Allergy/Antihistamines In Nasal Allergy	Fluticasone propionate/Azelastine hydrochloride	Dymista 137micrograms/dose / 50micrograms/dose nasal spray (Meda Pharmaceuticals Ltd)

For peer review only

Product code	BNF header	Drug substance	Drug product
55677	Selective Beta 2 Agonists/Corticosteroids (for Respiratory Conditions)	Salmeterol xinafoate/Fluticasone propionate	Seretide 500 Accuhaler (Lexon (UK) Ltd)
61280	Selective Beta 2 Agonists/Corticosteroids (for Respiratory Conditions)	Fluticasone propionate/Salmeterol xinafoate	Seretide 250 Accuhaler (Waymade Healthcare Plc)
62126	Selective Beta 2 Agonists/Corticosteroids (for Respiratory Conditions)	Salmeterol xinafoate/Fluticasone propionate	Seretide 100 Accuhaler (DE Pharmaceuticals)
63252	Selective Beta 2 Agonists/Corticosteroids (for Respiratory Conditions)	Fluticasone propionate/Salmeterol xinafoate	Seretide 250 Evohaler (Lexon (UK) Ltd)
63945	Selective Beta 2 Agonists/Corticosteroids (for Respiratory Conditions)	Fluticasone propionate/Salmeterol xinafoate	Seretide 250 Accuhaler (Lexon (UK) Ltd)
64372	Selective Beta 2 Agonists/Corticosteroids (for Respiratory Conditions)	Fluticasone propionate/Salmeterol xinafoate	Sirdupla 25micrograms/dose / 125micrograms/dose inhaler (Mylan Ltd)
64373	Selective Beta 2 Agonists/Corticosteroids (for Respiratory Conditions)	Fluticasone propionate/Salmeterol xinafoate	Sirdupla 25micrograms/dose / 250micrograms/dose inhaler (Mylan Ltd)
65117	Selective Beta 2 Agonists/Corticosteroids (for Respiratory Conditions)	Fluticasone propionate/Salmeterol xinafoate	Seretide 125 Evohaler (Mawdsley-Brooks & Company Ltd)

For peer review only

STROBE 2007 (v4) Statement—Checklist of items that should be included in reports of *cohort studies*

Section/Topic	Item #	Recommendation	Reported on page #
**Title and abstract**	1	(*a*) Indicate the study's design with a commonly used term in the title or the abstract	1
		(*b*) Provide in the abstract an informative and balanced summary of what was done and what was found	3 (note: is a protocol for an observational study so here and subsequently is reported *what is planned to be done* only)
**Introduction**			
Background/rationale	2	Explain the scientific background and rationale for the investigation being reported	5
Objectives	3	State specific objectives, including any prespecified hypotheses	6
**Methods**			
Study design	4	Present key elements of study design early in the paper	6
Setting	5	Describe the setting, locations, and relevant dates, including periods of recruitment, exposure, follow-up, and data collection	6
Participants	6	(*a*) Give the eligibility criteria, and the sources and methods of selection of participants. Describe methods of follow-up	7 - 10
		(*b*) For matched studies, give matching criteria and number of exposed and unexposed	7 - 10
Variables	7	Clearly define all outcomes, exposures, predictors, potential confounders, and effect modifiers. Give diagnostic criteria, if applicable	13 - 14
Data sources/ measurement	8*	For each variable of interest, give sources of data and details of methods of assessment (measurement). Describe comparability of assessment methods if there is more than one group	6
Bias	9	Describe any efforts to address potential sources of bias	14 - 16
Study size	10	Explain how the study size was arrived at	14
Quantitative variables	11	Explain how quantitative variables were handled in the analyses. If applicable, describe which groupings were chosen and why	11

For peer review only

Statistical methods	12	(*a*) Describe all statistical methods, including those used to control for confounding	14 - 16
		(*b*) Describe any methods used to examine subgroups and interactions	14 - 16
		(*c*) Explain how missing data were addressed	16
		(*d*) If applicable, explain how loss to follow-up was addressed	NA
		(*e*) Describe any sensitivity analyses	14 - 16
**Results**			***NA (is a protocol for an observational study)***
Participants	13*	(a) Report numbers of individuals at each stage of study—eg numbers potentially eligible, examined for eligibility, confirmed eligible, included in the study, completing follow-up, and analysed	
		(b) Give reasons for non-participation at each stage	
		(c) Consider use of a flow diagram	
Descriptive data	14*	(a) Give characteristics of study participants (eg demographic, clinical, social) and information on exposures and potential confounders	
		(b) Indicate number of participants with missing data for each variable of interest	
		(c) Summarise follow-up time (eg, average and total amount)	
Outcome data	15*	Report numbers of outcome events or summary measures over time	
Main results	16	(*a*) Give unadjusted estimates and, if applicable, confounder-adjusted estimates and their precision (eg, 95% confidence interval). Make clear which confounders were adjusted for and why they were included	
		(*b*) Report category boundaries when continuous variables were categorized	
		(*c*) If relevant, consider translating estimates of relative risk into absolute risk for a meaningful time period	
Other analyses	17	Report other analyses done—eg analyses of subgroups and interactions, and sensitivity analyses	
**Discussion**			***NA (is a protocol for an observational study)***
Key results	18	Summarise key results with reference to study objectives	
**Limitations**			***NA (is a protocol for an observational study)***
Interpretation	20	Give a cautious overall interpretation of results considering objectives, limitations, multiplicity of analyses, results from similar studies, and other relevant evidence	

For peer review only

Generalisability	21	Discuss the generalisability (external validity) of the study results	
**Other information**			
Funding	22	Give the source of funding and the role of the funders for the present study and, if applicable, for the original study on which the present article is based	2

*Give information separately for cases and controls in case-control studies and, if applicable, for exposed and unexposed groups in cohort and cross-sectional studies.

Note: An Explanation and Elaboration article discusses each checklist item and gives methodological background and published examples of transparent reporting. The STROBE checklist is best used in conjunction with this article (freely available on the Web sites of PLoS Medicine at http://www.plosmedicine.org/, Annals of Internal Medicine at http://www.annals.org/, and Epidemiology at http://www.epidem.com/). Information on the STROBE Initiative is available at www.strobe-statement.org.

For peer review only
